# Multimodal S(VI) Exchange Click Reactions Derived from SF_2_ Moieties: Comparative Kinetics and Stereochemistry of SuFEx and SuPhenEx Reactions

**DOI:** 10.1021/acs.joc.5c00615

**Published:** 2025-07-11

**Authors:** Yumei Zhu, Akash Krishna, Yang Chao, Xixi Li, Sidharam P. Pujari, Guanna Li, Hong Huang, Hongxia Zhao, Jiajia Dong, Han Zuilhof

**Affiliations:** † School of Pharmaceutical Science & Technology, 12605 Tianjin University, 92 Weijin Road, Nankai District, Tianjin 300072, China; ‡ College of Biological and Chemical Engineering, 66559 Jiaxing University, Jiaxing 314001, China; § Laboratory of Organic Chemistry, Wageningen University, Stippeneng 4, Wageningen 6708 WE, The Netherlands; ∥ Key Laboratory of Fluorine and Nitrogen Chemistry and Advanced Materials, Shanghai Institute of Organic Chemistry, University of Chinese Academy of Science, Chinese Academy of Sciences, 345 Lingling Lu, Shanghai 200032, China; ⊥ Biobased Chemistry and Technology, 4508 Wageningen University, Stippeneng 4, Wageningen 6708 WE, The Netherlands; # Institute of Translational Medicine, National Facility for Translational Medicine (Shanghai) and School of Chemistry and Chemical Engineering, Zhangjiang Institute for Advanced Study, 12474 Shanghai Jiao Tong University, Shanghai 200240, China

## Abstract

The reaction mechanism of multimodal SuFEx and SuPhenEx click chemistries was investigated in detail for substitution of both the first and second fluoride or *p*-nitrophenolate leaving groups in ∼RN = SOF_2_ and RN = SOF­(*p*-NO_2_-phenol) substrates by temperature-dependent kinetics and density functional theory (DFT) calculations. Both the DFT calculations and the experimentally derived Δ*H* ^‡^ indicate relatively small values for the activation enthalpies (4–15 kcal/mol), but almost constant and significant values of −*T*Δ*S*_25_^‡^ (10–14 kcal/mol). This indicates that depending on the precise nucleophile and S­(VI) core, the reaction is either controlled by a mixture of enthalpy and entropy or basically is dominated by entropy alone. Finally, we show using chiral HPLC and X-ray crystallography that the SuFEx reaction on chiral RN = SOF­(*p*-substituted-phenol) substrates also proceeds enantiospecifically, opening up also multimodal click chemistry to chiral applications in chemical biology and materials science.

## Graphical abstract

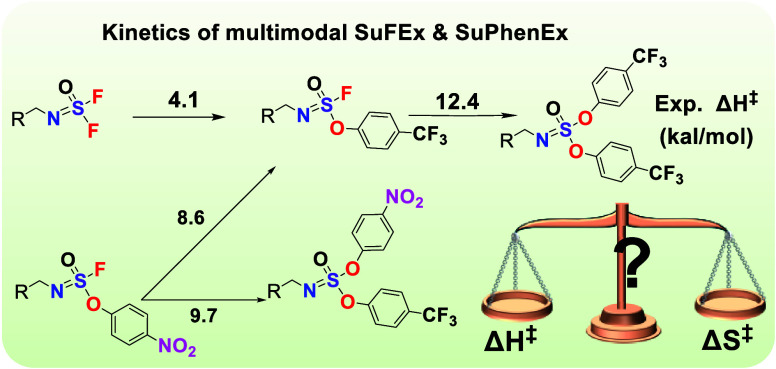

## Introduction

Click chemistry has significantly transformed materials science by providing an effective approach to structures with a wide range of molecular diversity.^1–3^ Its repertoire continues to expand, highlighted by the Cu­(I)-catalyzed azide–alkyne cycloaddition (CuAAC),^4^ and various metal-free alternatives such as thiol–ene and thio-Michael additions,^3,5^ oxime ligations,^6^ inverse electron-demand Diels–Alder reactions,^7^ and strain-promoted cycloadditions like SPOCQ^2^ or SPAAC.^8^ Recent expansions of that toolbox include the sulfur­(VI) fluoride exchange (SuFEx)^9^ and sulfur phenolate exchange (SuPhenEx)^10–12^ reactions. These two click reactions have emerged as powerful tools in organic synthesis, enabling the efficient formation of diverse sulfur-containing compounds.

SuFEx reactions are characterized by the use of sulfur­(VI) fluoride reagents, which readily undergo substitution reactions with a variety of nucleophiles. This class of click reactions is particularly valuable due to its high functional group tolerance and the ability to generate complex molecular architectures.^9,13–15^ Both in the field of chemical biology,^16,17^ and material science,^18–23^ SuFEx has been proven to be extremely powerful, especially since the use of chiral sulfonimidoyl fluorides has shown the substitution reaction to take place enantiospecifically.^24^

If the reacting nucleophile in a SuFEx reaction is weak but still quantitatively effective, like *p*-nitrophenolate, this can also act as a good leaving group, potentially initiating a SuPhenEx reaction.^10^ Such reactions have been shown to be synthetically valuable in the construction of polymers, sequence-defined oligomers,^23^ and chiral macrocycles,^25^ and introduced dynamic covalent chemistry characteristics to S­(VI) exchange chemistries.^10^ Since this substitution reaction is also enantiospecific, it allows easy access to both enantiomer products (via single and double substitution, respectively) starting from a single chiral S–F compound.^25^ The reaction rates of SuFEx and SuPhenEx reactions are significantly influenced by factors such as solvent choice, nucleophilic strength, and the electronic properties of substituents on the substrate.^10,24,26–29^ Recent studies have quantitatively analyzed the activation enthalpies associated with SuFEx and SuPhenEx reactions, revealing that both SuFEx and SuPhenEx reactions are bimolecular, fast, and slightly exothermic click reactions, making them amenable to a wide range of substrates.^10,24^

In this field, multimodal SuFEx chemistry has attracted specific attention as it turns ∼SF_2_ moieties of, e.g., iminosulfur oxydifluorides (RN = SOF_2_) into doubly substitutable groups. This has, for example, been used in a wide variety of chemical biology studies,^17,30,31^ and in the efficient formation of polymers using the first F in di-iminosulfur oxydifluorides (F_2_OS= N–N =SOF_2_), while substitution of the second F allowed for postpolymerization modification.^19,32^ The success of polymer formation followed by postpolymerization modification—typically using other nucleophiles—over the uncontrolled formation of a network, indicates that when one S–F bond is exchanged for an O/N-based nucleophile, the reactivity of the resulting compound (e.g., R–N = SOF–O–Ar if that nucleophile was a phenol) is significantly weakened,^17,19,32,33^ but a quantification thereof has hitherto been missing.

Recent studies have highlighted the significance of such rate differences in various fields, including SF_2_ groups acting as initiator for the first reported chain-growth SuFEx polycondensation and AB-type aryl silyl ether-fluorosulfates with electron-withdrawing groups as monomers, exploiting S–F bond reactivity and selectivity under SuFEx catalysis.^32^ In the field of medicinal chemistry, e.g., the iminosulfur oxydifluoride cores and amines were used in SuFEx reactions to create a library of sulfamide or sulfur-amidimidoyl fluoride compounds, enabling direct in vitro enzyme assays.^17^ Finally, an asymmetric three-dimensional (3D)-SuFEx reaction using thionyl tetrafluoride gas and chiral ligand-induced enantioselective defluorinative substitution of iminosulfur oxydifluorides with organolithium reagents was reported, which enables modular synthesis of optically active S­(VI) functional molecules.^34^

Despite these successes, the development of multimodal S­(VI) chemistries was, however, strongly hampered, since the reagents originated from SOF_4_, a highly corrosive gas. Yet, three things have changed this. First, efficient handling procedures for this gas have been developed, allowing its more ready small-scale use.^35^ Second, Ismalaj, De Borggraeve and Demaerel have described the formation of [Ag···SOF_4_],^29^ which displays efficient SuFEx reactivity, while third, Miloserdov and Zuilhof introduced the efficient synthesis and reactivity of a bench-stable imidazole-fluoride-substituted S­(VI) agent, which also allows multimodal S­(VI) exchange, and introduced enantiospecificity also to multimodal S­(VI) exchange reactions.^30^

This evident growth in relevance and ease of use thus contrasts sharply with the paucity of data that characterize these multimodal substitution reactions in detail, hampering their use and further optimization. This prompted us to study in the current paper the respective reactivities of the first and second leaving groups in (*p*-biphenyl)–CH_2_–N = SOXY compounds (X, Y = F, *p*-NO_2_-phenolate) by both in-depth temperature-dependent kinetics in acetonitrile, and by density functional theory (DFT) calculations. DFT calculations were carried out to determine the activation barriers and reaction energies using the Gaussian16 program^36^ by employing the long-range-corrected ωB97XD functional in combination with the triple-ζ 6–311+G­(d,p) basis set, and an SMD model mimicking acetonirile. Specifically, we target the kinetics and activation enthalpies of the SuFEx and SuPhenEx reactivity arising from iminosulfur oxydifluoride (RN = SOF_2_) **1** and its S­(VI) exchange products (see Scheme 1). In doing so, we obtain insight into the role of the activation enthalpy Δ*H* ^‡^ and the activation entropy Δ*S* ^‡^, and in the competition between SuFEx and SuPhenEx reactivity in the second substitution reaction. Finally, combining DFT calculations, chiral HPLC, and crystallography, we demonstrate that the second SuFEx reaction is also enantiospecific and takes place with inversion.

**Scheme 1:**
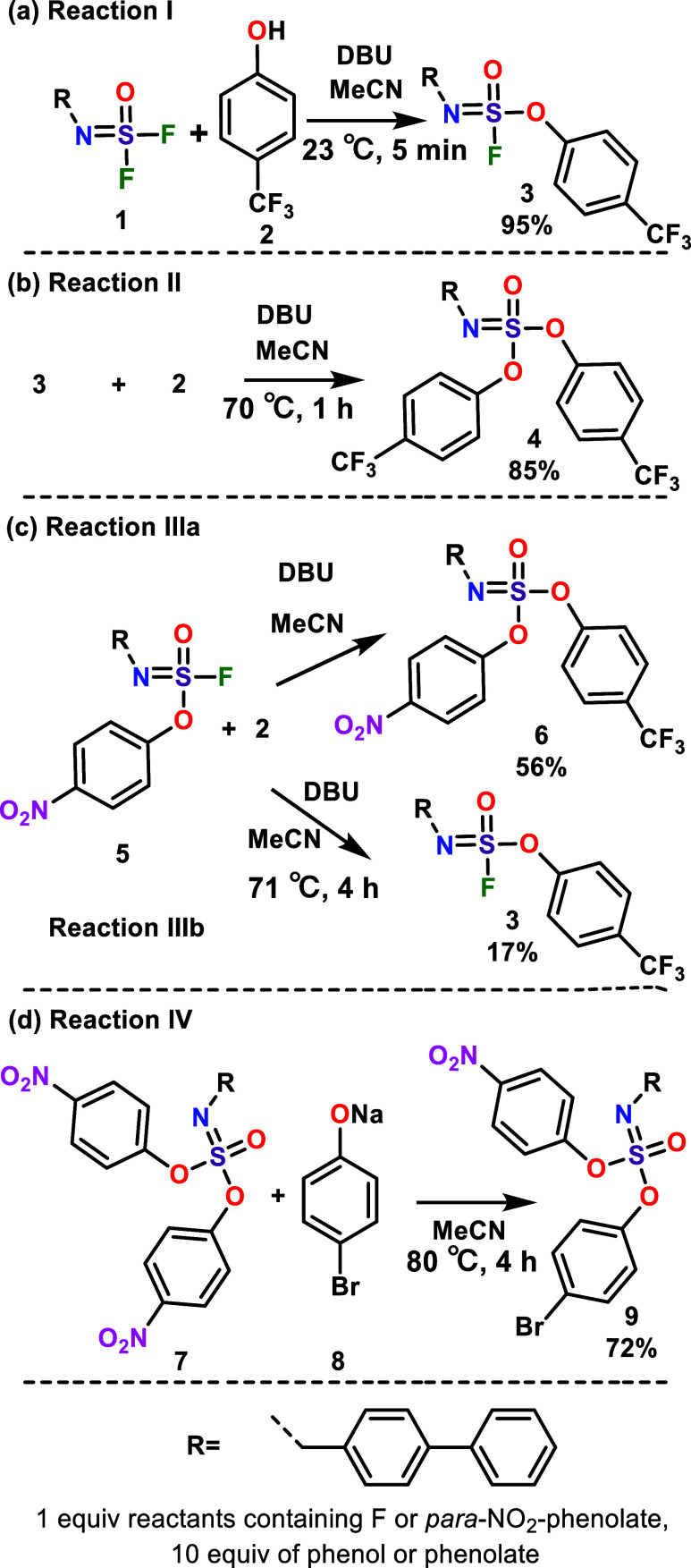
Model Reactions Employed for Kinetic Studies of (a) First (Reaction **I**) and (b) Second F (Reaction **II**) SuFEx Reactions in Iminosulfur Oxydifluoride **1**, (c) Second Substitution Reactions in Compound **5**; SuFEx Leading to **6** (Reaction **IIIa**), and SuPhenEx Leading to **3** (Reaction **IIIb**), (d) First SuPhenEx Reaction in Compound **7** (Reaction **IV**)­^a^

a All reactions were performed in acetonitrile.

## Results and Discussion

As a first step, we aimed to provide a detailed comparison to the various reactions. These display rates that are highly sensitive to the nucleophile, so that some tuning was required to get robustly measurable rates by one approach and from one nucleophile for all reactions **I**, **II**, and **III**. Reaction **I** is complete within 15 min at 20 °C even with a relatively poor nucleophile such as *p*-nitrophenol. In contrast, reaction **II** would proceed slowly at such a temperature, making proper temperature control at the same temperature range required to study reaction **I** in detail harder. As a result, *p*-CF_3_-phenol **2** in the presence of DBU was found to be a well-behaved system for all of these reactions, while different temperature ranges were used for the construction of Eyring plots (obtained via the pseudo-first-order rate constants measured using a 10-fold excess of the relevant phenol) for the various reactions: from 16 to 47 °C for **I**, from 40 to 70 °C for reaction **II**, from 41 to 71 °C for reactions **IIIa** and **IIIb**, and from 50 to 80 °C for reaction **IV**. The plots from the Eyring equation are given in Figure 1. For all reactions under study, the Eyring plots are linear. Therefore, the slope of these plots (−Δ*H* ^‡^/*R*) provides the activation enthalpy Δ*H* ^‡^, while Δ*S* ^‡^ can be determined from the y-intercept. Measurement of or extrapolation to a rate at 25 °C then provides −*T*Δ*S*_25_^‡^, and thereby also Δ*G*_25_^‡^. The resulting experimental data are given in Table 1, together with the DFT-computed theoretical values for Δ*H* ^‡^. The Eyring equation is given by eq 1 ^35^

1 $$\ln\frac{k}{T}=-\frac{{\Delta H}^{‡}}{R}\frac{1}{T}+\ln\frac{k_{B}}{h}+\frac{{\Delta S}^{‡}}{R}$$

and describes how the temperature dependence of reaction rates is related to the activation parameters Δ*H* ^‡^ and Δ*S^‡^*, and therefore Δ*G* ^‡^. As a result, from the slope and the intercept of plots of the rate versus 1/*T* all activation parameters can be obtained (see also Supporting Information, Section 3.4).

**Figure 1:**
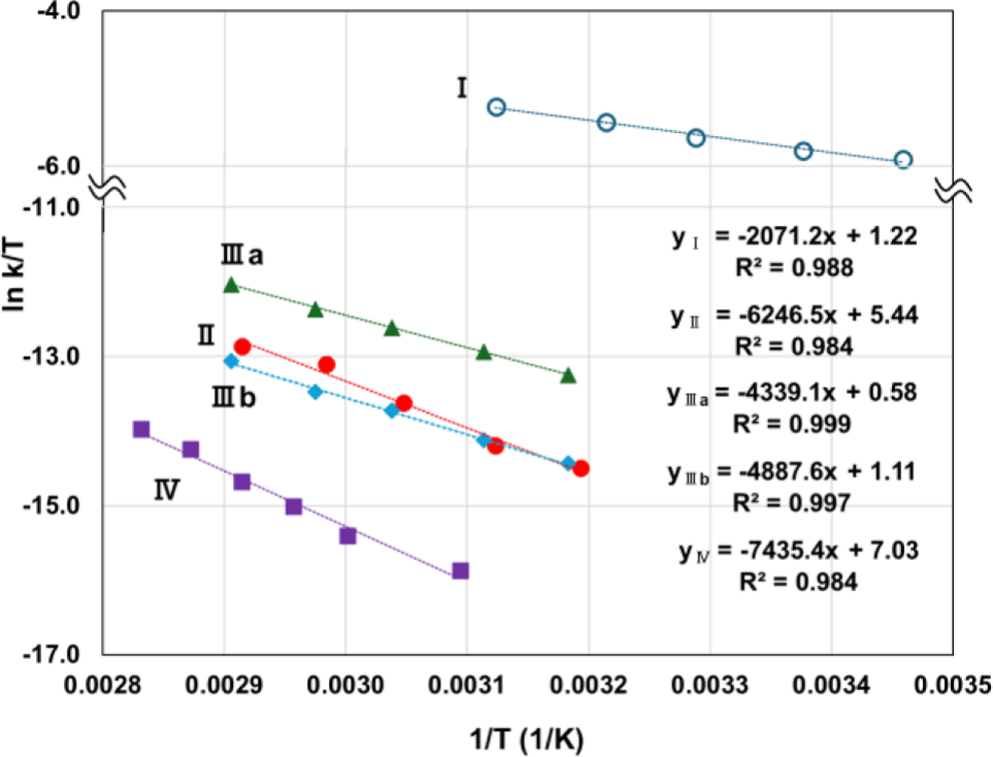
Eyring plots using the kinetic data of the reactions under study (all in acetonitrile).

**Table 1:** Experimental Activation Parameters and Calculated Δ*H* ^‡^ for Reactions I to IV (All in kcal/mol)

	experimental			DFT
Rxn	Δ*H* ^‡^	–*T*Δ*S*_25_^‡^	Δ*G*_25_^‡^	Δ*H*_calc_^‡^
**I**	4.1	13.4	17.5	2.3
**II**	12.4	10.8	23.2	12.5
**IIIa**	8.6	13.7	22.3	8.4
**IIIb**	9.7	13.4	23.1	11.6
**IV**	14.8	10.0	24.8	14.2

The data in Table 1 allow us to draw several conclusions. First, utilizing both experimental and theoretically calculated activation enthalpy values presented in the table, we deduce a relative ranking of the reaction rates for these five reactions: **I** > **IIIa** ≈ **IIIb** > **II** > **IV**. Reaction **I** is fastest, like the presence of two highly electronegative F atoms on the S­(VI) atom, thus increasing nucleophilic attack. Since SuFEx reaction **IIIa** substitutes a F atom on a S­(VI) atom that carries a *p*-nitrophenolate substituent, whereas reaction **II** does the same thing but now with a slightly less electron-withdrawing *p*-CF_3_ substituent, the experimentally observed data are in line with the Hammett σ-constants (*p*-NO_2_ = 0.78; *p*-CF_3_ = 0.54).^32^ Interestingly, the reaction is about only slightly slower than the SuFEx reaction on **5**, displaying the power of such SuPhenEx reactions, which actually take place with even a lower Δ*H* ^‡^ than the SuFEx reaction on **3**. Finally, the SuPhenEx reaction on di-*p*-NO_2_-phenolate compound **7** is the slowest among those reactions studied, but only by about 2–6 kcal/mol compared to reactions **II**, **IIIa**, and **IIIb**.

Second, the energy values corresponding to the entropy of activation -*T*Δ*S* ^‡^ for these five reactions are all positive, signifying that the entropy of all TSs under study decreases as to be expected from a bimolecular substitution reaction with two interacting species in the TS. The values are all somewhat similar to values of 10–14 kcal/mol. Second, the values of Δ*H* ^‡^ for the corresponding reactions increase from 4.1 kcal/mol for reaction **I** to 14.8 kcal/mol for reaction **IV**. These ranges imply that several of these reactions are really entropy-controlled (e.g., **I**), while others are in fact dominated by enthalpic contributions (e.g., **II** and **IV**). This is an important finding, as entropy control is typically not envisaged when discussing rate differences between various click reactions, although evidently it should.

Based on this observation, it is not trivial to kinetically analyze even such highly analogous S­(VI) exchange reactions as the ones under current investigation within a simple conceptual frame.

To better understand these trends, we approximated the TSs of these reactions using quantum chemical calculations and density functional ωB97XD/6–311+G­(d,p) calculations. [Further increase in basis set size up to def2-QZVPP is computationally more costly, but does not lead to significantly different results (Table S17)]. This provided values that accurately approached the experimental values (average deviation of 0.88 kcal/mol), suggesting that the depiction provides accuracy.

Compound **1** facilitates a swift SuFEx reaction with **2**. Using the Eyring equation based on reaction rates at temperatures ranging from 16 to 45 °C, the activation enthalpy (Δ*H* ^‡^) for this first SuFEx reaction was determined to be only 4.1 kcal/mol, in good agreement with the calculated value of 2.3 kcal/mol, and similar to previous SuFEx reaction data on sulfonimidoyl fluorides.^24^ Depictions of the latter as being generally slower than many other S–F compounds^33^ are thus difficult to reconcile with the current data. Further analogies can be observed when tracking the reaction profile quantum chemically. In reaction **I**, phenolate **2** attacks -SF_2_ compound **1** following an addition–elimination pathway via **TS1** with a rate-limiting activation enthalpy of only 2.3 kcal mol^–1^ (Figure 2).^16^ This is then calculated to lead to an addition–elimination reaction, as the first intermediate **INT1** with a five-coordinated sulfur center is formed, which rapidly releases a fluoride ion through **TS2**, which has a low enthalpic activation barrier of only 2.0 kcal mol^–1^, ultimately leading to the final product, with all calculated barriers so low that experimentally an S_N_2 mechanism will likely be observed.

**Figure 2:**
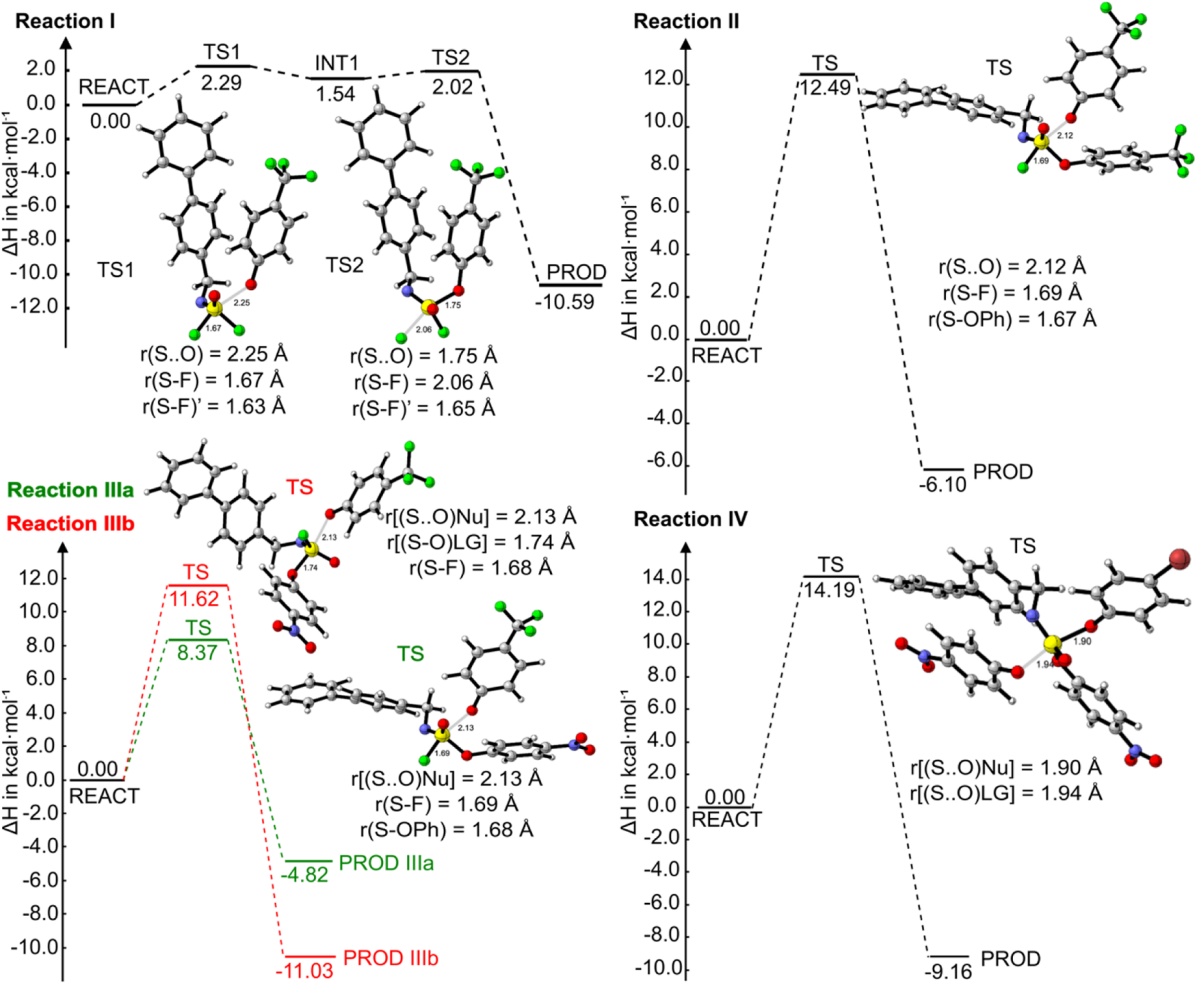
Energy profiles for reactions **I** and **II** (first and second SuFEx reactions in iminosulfur oxydifluoride **1**), reaction **IIIa** (SuFEx leading to compound **6**), reaction **IIIb** (SuPhenEx leading to compound **3**), and reaction **IV** (first SuPhenEx reaction in compound **7**), all computed at the SMD- ωB97XD/6–311+G­(d,p) level of theory using acetonitrile as the solvent.

The Δ*H* ^‡^ for the subsequent SuFEx reaction onto compound **3** (Reaction **II**) was determined to be 12.4 kcal/mol (calculated value: 12.5 kcal/mol), i.e., significantly higher than that of reaction **I**, This is likely both due to the diminished electrophilicity of the central S­(VI) atom due to substituting one electronegative F with a *p*-CF_3‑_phenolate moiety, but also to the increased steric effects that are highly relevant in both S_N_2 or addition–elimination mechanisms (see also Figure 2 - Reaction **II**).

Our DFT calculations elucidate the substrate-dependent dual role of DBU in the SuFEx reactions. In Reaction **I**, DBU acts principally as a Brønsted base, deprotonating phenol to form the reactive phenolate nucleophile, but without [DBU + H]^+^ playing a significant role in the nucleophilic substitution step, as, e.g., noted by near-identical activation barriers, and a very long *r*(H···F = 3.9 Å) in the TS. In contrast, Reaction **II** follows an alternative pathway where—in contrast to unprotonated DBU which is inactive in the substitution step—[DBU + H]^+^ stabilizes the transition state through hydrogen bonding to the departing fluoride (*r*(H···F) = 1.85 Å; *r*(S–F) = 1.85 Å), reducing Δ*H* ^‡^ by 6.2 kcal/mol (from 12.5 to 6.3 kcal/mol) relative to the uncatalyzed process. This strong reduction is, however, not observed experimentally, which we tentatively attribute to the specific H-bond stabilization of [DBU + H]^+^ by the cyano moieties of the acetonitrile solvent. As an implication of these findings, it is thus expected that the role of DBU in SuFEx reactions is thus both substrate-dependent and solvent-dependent.

For a comparative analysis, we examined the activation energies of the SuFEx and SuPhenEx reactions, where a fluorine group and *p*-NO_2_ are attached to the same sulfur atom in compound **5**, and either of them is substituted by a phenolate **2** (illustrated in Scheme 1, Reactions **IIIa** and **IIIb**). Interestingly, the activation enthalpies for both reactions are closely matched, with the SuFEx reaction at 8.6 kcal/mol and the SuPhenEx reaction at 9.7 kcal/mol; i.e., the SuFEx reaction is only slightly faster than the SuPhenEx reaction. This was also found by DFT studies, which indicated that both reactions occur via a single-step S_N_2 mechanism with Δ*H* ^‡^ values of 8.4 kcal/mol (SuFEx) and 11.6 kcal/mol (SuPhenEx), respectively (Figure 2). This slight variation in activation barriers for S–F and S–O bond dissociation is supported by, e.g., the difference in the Weiberg bond orders of the S–F and S–O bonds (0.589 versus 0.763, respectively).

We also noted that, in our practical experience, the SuPhenEx reaction might be slightly slower than the corresponding SuFEx reaction,^10^ but that in the case under current investigation, this effect is partially compensated by the highly activating electron-withdrawing effect of F. Therefore, we also wanted to study the rate of the di-*p*-NO_2_ phenolate compound **7**. This compound turned out to be unstable under alkaline conditions, so we transitioned to using sodium *p*-CF_3_-phenolate for the reaction kinetics studies. This reaction is clearly slower, and stirring the mixture of compound **7** and *p*-trifluoromethylphenolate at 70 °C for 2 h showed that no new compound formed. Turning to a good nucleophile, like *p*-methoxyphenolate, the species was actually very reactive, so that the second *p*-NO_2_ reacts rapidly with the phenolate, making it less suitable for obtaining kinetic data. We therefore decided to use *p*-bromophenolate to react with compound **7**, even though the reaction between the first *p*-NO_2_ functional group and *p*-bromophenolate takes several hours to complete (Scheme 1, Reaction **IV**). The experimental activation enthalpy of 14.8 kcal/mol closely matched the calculated enthalpic barrier of 14.2 kcal mol^–1^. The reaction was calculated to proceed via an S_N_2 mechanism, and in this, we note that there is indeed some steric hindrance that causes the enthalpic barrier to increase (Figure 2, Reaction **IV**).

We finally determined the stereochemistry of the second SuFEx chemistry, as this is relevant for, e.g., materials and chemical biology applications in which −SF_2_ compounds are involved in double, likely sequential, substitution reactions with two different nucleophiles. To this aim, the two enantiomers of compound **3** were isolated into two compounds **(*R*)-** **3** and **(*S*)-** **3** by preparative chiral HPLC, and subsequently reacted separately with a range of *p*-substituted (R = H, F, Cl, Br) phenolates. To our delight with all these phenolates, each of the enantiomers gave only one product (*ee* >99% by chiral HPLC), which, according to our calculations, would be the product obtained after inversion. To demonstrate this inversion, crystals were grown of **(*S*)-** **3** and **(*R*)-** **10**, the product resulting from the attack of *p*-Br-phenolate. These data (Figure 3) indeed showed an inversion of stereochemistry also in the second of these multimodal SuFEx reactions.

**Figure 3:**
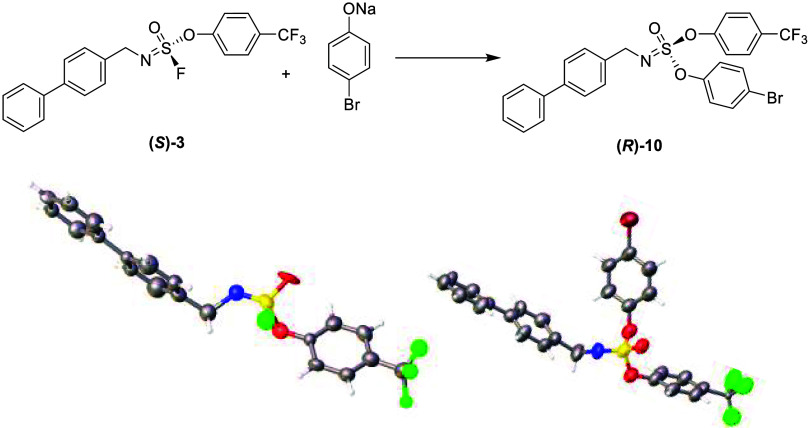
Chemical structure and X-ray crystal structures of **(*S*)-3** and (*R*)-**10** from the second SuFEx reaction. Color code: C (gray), O (red), S (yellow), N (blue), H (white), F (green), and Br (burgundy). Displacement ellipsoids are drawn at the 50% probability level.

## Conclusions

We have obtained detailed temperature-dependent kinetic data for multimodal SuFEx and SuPhenEx click chemistries, which clarify that depending on the precise nucleophile and S­(VI) core, the reaction is either controlled by a mixture of enthalpy and entropy or is basically dominated by entropy alone. The explicit inclusion of entropic effects in thinking about the efficiency of click reactions, as also noted for a series of strain-promoted click reactions,^36^ is an important step for the further development of such chemistries. Finally, we note that multimodal SuFEx click chemistry is enantiospecific, further adding to its potential use in advanced materials and chemical biology.

## Supplementary Material



## Data Availability

The data underlying this study are available in the published article and its Supporting Information.

## Notes

Experimental details, NMR spectra and data, single-crystal X-ray data, computational details, and Cartesian coordinates of all computed stationary points (PDF)
