# Supplementary material for: Multimodal S(VI) Exchange Click Reactions Derived from SF2 Moieties: Comparative Kinetics and Stereochemistry of SuFEx and SuPhenEx Reactions
Source: J Org Chem. 2025 Jul 11;90(29):10218–24. doi: 10.1021/acs.joc.5c00615 (PMC12305642; doi:10.1021/acs.joc.5c00615)
Supplement: Supplementary file 1 [file jo5c00615_si_001.pdf]

Supporting Information

Multimodal S(VI) Exchange Click Reactions derived from SF_2 moieties: Comparative Kinetics and Stereochemistry of SuFEx and SuPhenEx Reactions

Yumei Zhu,[a] Akash Krishna,[b,c] Yang Chao,[a] Xixi Li,[d] Sidharam P. Pujari,[b] Guanna Li,[e] Hong Huang,[b] Hongxia Zhao,[a] Jiajia Dong,*[f] and Han Zuilhof*[a,b,c]

[a]School of Pharmaceutical Science & Technology, Tianjin University, 92 Weijin Road, Nankai District, Tianjin, 300072, P. R. China.
[b]College of Biological and Chemical Engineering, Jiaxing University, Jiaxing, 314001, China.
[c]Laboratory of Organic Chemistry, Wageningen University, Stippeneng 4, 6708 WE, Wageningen, The Netherlands.
[d]Key Laboratory of Fluorine and Nitrogen Chemistry and Advanced Materials, Shanghai Institute of Organic Chemistry, University of Chinese Academy of Science, Chinese Academy of Sciences, 345 Lingling Lu, Shanghai 200032, China.
[e]Biobased Chemistry and Technology, Wageningen University, Stippeneng 4, 6708 WE Wageningen, The Netherlands.
[f]Institute of Translational Medicine, National Facility for Translational Medicine (Shanghai) and School of Chemistry and Chemical Engineering, Zhangjiang Institute for Advanced Study, Shanghai Jiao Tong University, Shanghai 200240, China.
E-mail: Jiajiadong@sjtu.edu.cn, han.zuilhof@wur.nl.

Table of contents

SI. No	Title	Page
1	Materials and general methods	S2
2	Synthesis of starting materials	S3-S7
3	Kinetic studies of first & second group in S(VI) for SuFEx and SuPhenEx reactions	S8-S22
4	Competition between SuFEx and SuPhenEx reaction	S22-S32
5	Reaction kinetics of first group in S(VI) for SuPhenEx reactions at different temperatures	S33-S38
6	Inversion of absolute configuration for SuPhenEx reaction	S39
7	NMR Spectra	S37-S51
8	Chiral HPLC data	S52-S54
9	X-Ray crystallography	S55-S58
10	DFT-computed theoretical values of activation enthalpies for all reactions	S59-S100
11	References	S101

1. Materials and General Methods

Starting materials, reagents, and solvents were purchased from commercial vendors and used as received, unless otherwise noted. All reactions were performed under an argon atmosphere and in dry solvents, unless otherwise stated. Analytical thin-layer chromatography (TLC) was performed on aluminum sheets, precoated with silica gel GF254. Preparative thin-layer chromatography (PTLC) separations were carried out on 200 × 200 mm, 1.0 mm XINNUO silica gel plates (GF-254). Flash column chromatography was performed over silica gel (200−300 mesh or 300−400 mesh). ^{1}H, $^{13}C\{^{1}H\}$ NMR and ^{19}F NMR spectra were recorded on Bruker Avance 400 MHz and 600 MHz spectrometers at 298 K, unless otherwise noted. The chemical shifts are listed in ppm on the δ scale and coupling constants were recorded in Hertz (Hz). Chemical shifts are calibrated relative to the signals corresponding of the non-deuterated solvents ($CHCl_3$: δ 7.26 ppm for ^{1}H and 77.16 ppm for $^{13}C\{^{1}H\}$ NMR, CH_3CN: δ 1.94 ppm for ^{1}H and 1.32 ppm and 118.26 ppm for $^{13}C\{^{1}H\}$ NMR). The following abbreviations were used for multiplicities: s, singlet; d, doublet; t, triplet; m, multiplet or overlapping peaks; b, broad peaks. High-resolution mass spectra (HRMS) were measured on a Q Exactive HF spectrometer using electrospray ionization (ESI) in positive and negative mode. UV-vis absorption spectra were recorded on an Agilent Cary 60 UV-Vis spectrophotometer using 1-cm quartz cell. High-performance liquid chromatography (HPLC) analyses were operated using an Agilent 1260 liquid chromatography system (Agilent Technologies, USA). HPLC grade isopropanol, acetonitrile and *n*-hexane were purchased from Concord Technology, Tianjin, P. R. China.

2. Synthesis of starting materials

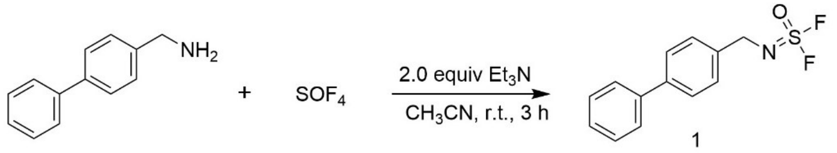

Scheme S1. Synthesis of compound **1**.

Compound 1: (1,1'-biphenyl]-4-ylmethyl) sulfurimidoyl difluoride (1): A 250 mL round-bottom flask was charged with [1,1′-Biphenyl]-4-methanamine (1.83 g, 10.0 mmol) and acetonitrile (60 mL). Et_3N (2.8 mL, 20.0 mmol) was added into the flask and the mixture was stirred vigorously for 10 minutes. The air in the flask was then evacuated under gentle vacuum until the solution began to degas. Sulfur tetrafluoride (SOF_4, approximately 500 mL, 35% purity)[1] was introduced to the system using a balloon, and the reaction mixture was stirred vigorously at room temperature (approx. 500 rpm) for 3 hours. During this period, the balloon deflated, and considerable white smoke was observed in the flask. Upon completion of the reaction (noting that due to the low purity, most of the amine was not fully converted), the balloon was removed, and the mixture underwent workup and purification. To quench the reaction, 3-4 g of 100-200 mesh silica gel was slowly added to quench the reaction while maintaining an ice bath. The mixture was stirred for 30 minutes. The solvent was then removed under reduced pressure using a rotary evaporator. The crude was further purified by column chromatography (*n*-hexane to *n*-hexane/DCM = 10:1 (gradient elution)) to provide 775 mg of white solid **1** (yield: 29%). R_f = 0.72 (EtOAc/n-hexane = 1:5). **^{1}H NMR** (400 MHz, $CDCl_3$) δ 7.66 – 7.56 (m, 4H), 7.50 – 7.40 (m, 4H), 7.37 (t, J = 7.3 Hz), 4.58 (t, J = 4.0 Hz, 2H). ^{19}F NMR (376 MHz, $CDCl_3$) δ 47.01 (t, J = 3.9 Hz). **^{13}C{^{1}H} NMR** (101 MHz, $CDCl_3$) δ 141.2, 140.7, 135.5, 129.0, 127.8, 127.6, 127.3, 49.2. **HRMS (EI+,** m/z): Calcd for $C_{13}H_{11}ONF_2S^+$: 267.0524 $[M]^+$, found: 267.0522. Melting point: 57.4-58.0 ℃.

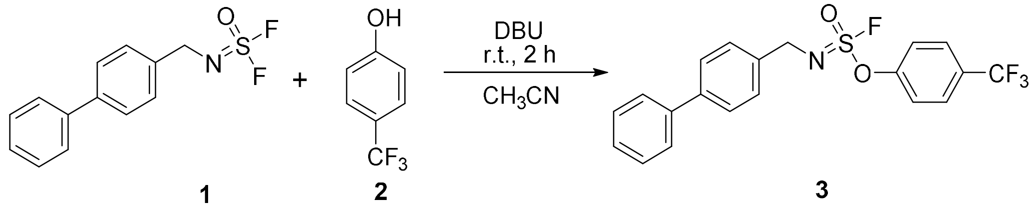

Scheme S2. Synthesis of compound **3**.

Compound 3: **1** (20.0 mg, 0.072 mmol, 1.0 equiv.) was dissolved in anhydrous CH_3CN (1 mL) under argon protection. Then 4-hydroxybenzotrifluoride (14.1 mg, 0.0864

mmol, 1.2 equiv.) and DBU (13.16 μL, 0.0864 mmol) were added to the solution. The reaction mixture was stirred for 2 h at room temperature. The reaction was quenched by adding water (1 mL), extracted with CH_2Cl_2 (3 × 1 mL), dried with anhydrous $MgSO_4$ and purified by column chromatography ((EtOAc/*n*-hexane = 1:6) to afford **3** as white solid (25.05 mg, 0.0612 mmol, 85%). R_f = 0.67 (EtOAc/n-hexane = 1:5). **^{1}H NMR** (400 MHz, $CDCl_3$) δ 7.73 (d, J = 8.8 Hz, 2H), 7.62 (dd, *J* = 7.6, 4.4 Hz 4H), 7.47 ((q, *J* = 8.2 Hz, 6H), 7.39 (t, *J* = 7.4 Hz, 1H), 4.64 (d, *J* = 4.6 Hz, 2H). **^{19}F NMR** (376 MHz, $CDCl_3$) δ 51.54 (t, *J* = 4.8 Hz,), 62.50(s). **^{13}C{^{1}H} NMR** (101 MHz, $CDCl_3$) δ 152.5, 140.8, 140.7, 136.9, 129.0, 127.8, 127.7, 127.5, 127.5, 127.2, 122.0(s), 49.2(s). **HRMS(ESI)**: [M-H]$^-$ m/z: Calcd for $C_{20}H_{14}F_4NO_2S^-$: 408.0687, Found: 408.0694. Melting point: 67.0-67.2 °C.

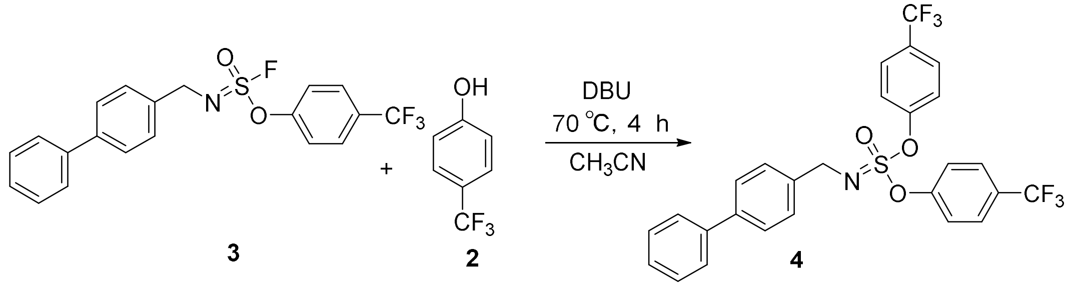

Scheme S3. Synthesis of compound **4**.

Compound 4: **3** (12.5 mg, 0.03 mmol, 1.0 equiv.) was dissolved in anhydrous CH_3CN (1 mL) under argon protection. Then 4-hydroxybenzotrifluoride (5.9 mg, 0.036 mmol, 1.2 equiv.) and DBU (5.48 μL, 0.036 mmol) were added to the solution. The reaction mixture was stirred for 4 h at 40 °C. The reaction was quenched by adding water (1 mL), extracted with CH_2Cl_2 (3 × 1 mL), dried with anhydrous $MgSO_4$ and purified by column chromatography ((EtOAc/*n*-hexane = 1:6) to afford **4** as white solid (11.6 mg, 0.021 mmol, 70%). R_f = 0.57 (EtOAc/n-hexane = 1:4). **^{1}H NMR** (400 MHz, CD_3CN) δ 7.75 (d, *J* = 8.4 Hz, 4H), 7.60 (d, *J* = 7.1 Hz, 2H), 7.51 (dd, *J* = 16.3, 8.3 Hz, 6H), 7.44 (t, *J* = 7.6 Hz, 2H), 7.35 (d, *J* = 7.5 Hz, 1H), 7.31 (d, *J* = 8.6 Hz, 2H), 4.55 (s, 2H). **^{19}F NMR** (376 MHz, CD_3CN) δ -62.85(s). **^{13}C{^{1}H} NMR** (101 MHz, CD_3CN) δ 153.9, 141.5, 140.8, 139.2, 129.9, 128.9(s), 128.6, 128.4, 127.9, 123.3, 49.7. **HRMS (ESI)** m/z: [M-H]$^-$ Calcd for $C_{27}H_{18}F_6NO_3S^-$: 550.0917, Found: 550.0926. Melting point: 61.8-62.4 °C.

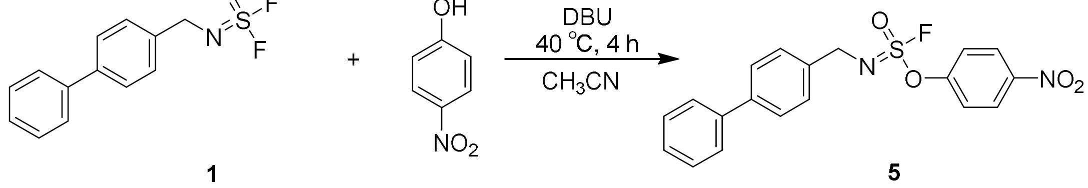

Scheme S4. Synthesis of compound **5**.

Compound 5: **1** (100 mg, 0.36 mmol, 1.0 equiv.) was dissolved in anhydrous CH_3CN (5 mL) under argon protection. Then 4-nitrophenol (56.8 mg, 0.4 mmol, 1.1 equiv.) and DBU (60.96 μL, 0.4 mmol) were added to the solution. The reaction mixture was stirred for 4 h at 40 °C. The reaction was quenched by adding water (5 mL), extracted with CH_2Cl_2 (3 × 5 mL), dried with anhydrous $MgSO_4$ and purified by column chromatography ((EtOAc/*n*-hexane = 1:9) to afford **5** as light yellow solid (108.2 mg, 0.28 mmol, 76%), R_f = 0.46 (EtOAc/n-hexane = 1:4). **^{1}H NMR** (400 MHz, $CDCl_3$) δ 8.32 (d, *J* = 9.2 Hz, 2H), 7.58 (d, *J* = 8.4 Hz, 4H), 7.51-7.39 (m, 6H), 7.36 (t, *J* = 7.3 Hz, 1H), 4.61 (d, *J* = 4.5 Hz, 2H). **^{19}F NMR** (376 MHz, $CDCl_3$) δ 52.66. **^{13}C{^{1}H} NMR** (101 MHz, $CDCl_3$) δ 154.2 (s), 146.8,140.9, 140.7,136.6, 129.0, 127.9, 127.6, 127.5, 127.2, 126.0, 126.0, 122.4, 49.3. **HRMS (ESI,** m/z**)** m/z: [M - H]$^-$ Calcd for $C_{19}H_{14}FN_2O_4S^-$: 385.0664, Found: 385.0671. Melting point: 61.6-62.0 °C.

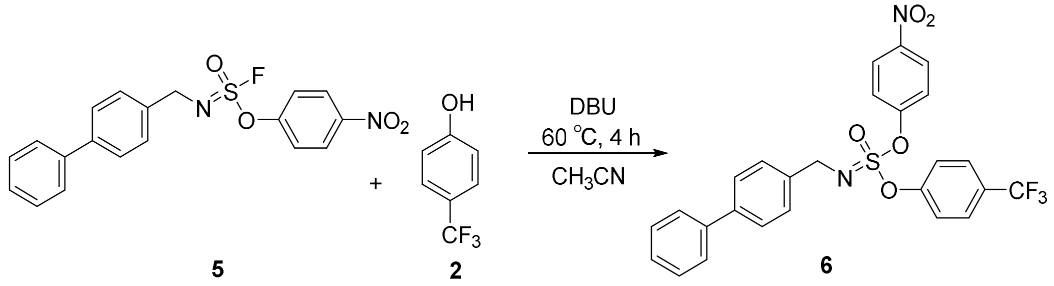

Scheme S5. Synthesis of compound **6**.

Compound 6: **5** (10.0 mg, 0.026 mmol, 1.0 equiv.) was dissolved in anhydrous CH_3CN (1 mL) under argon protection. Then 4-nitrophenol (8.48 mg, 0.052 mmol, 2.0 equiv.) and DBU (7.92 μL, 0.052 mmol) were added to the solution. The reaction mixture was stirred for 4 h at 60 °C. The reaction was quenched by adding water (1 mL), extracted with CH_2Cl_2 (3 × 1 mL), dried with anhydrous $MgSO_4$ and the crude was further purified by preparative TLC plate (n-hexane: EtOAc) to provide compound **6** as pale yellow solid (8.24 mg, 0.0156 mmol, 60%). Rf = 0.40 (EtOAc/n-hexane = 1:4). **^{1}H NMR** (400 MHz, CD_3CN) δ 8.26 (d, *J* = 9.3 Hz, 2H), 7.77 (d, *J* = 9.2 Hz, 2H), 7.61 (d, *J* = 7.1 Hz, 2H), 7.57 – 7.48 (m, 6H), 7.45 (t, *J* = 7.5 Hz, 2H), 7.40 – 7.33 (m, 1H), 7.32 (d, *J* = 8.1 Hz, 2H), 4.56 (s, 2H). **^{19}F NMR** (376 MHz, CD_3CN) δ -62.90. **^{13}C{^{1}H} NMR** (101 MHz, CD_3CN) δ 155.5, 141.5, 140.8, 139.1, 129.9, 128.9, 128.6, 128.4, 127.9, 127.8, 126.8, 123.4, 49.8. **HRMS (ESI,** m/z**)**: [M+H]$^+$ Calcd for $C_{26}H_{20}O_5N_2F_3S^+$: 529.1039 [M]+, Found: 529.1020.

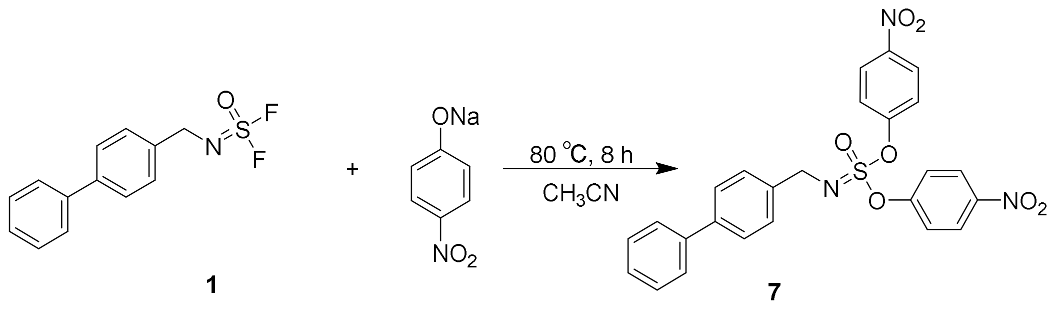

Scheme S6. Synthesis of compound **7**.

Compound 7: 1 (162.9 mg, 0.61 mmol, 1.0 equiv.) was dissolved in anhydrous CH_3CN (15 mL) under argon protection. Then sodium *para*-nitro phenolate (394.3 mg, 2.44 mmol, 4.0 equiv.) was added to the solution. The reaction mixture was stirred for 8 h at 80 °C. The reaction was quenched by adding water (15 mL), extracted with CH_2Cl_2 (3 × 15 mL), dried with anhydrous $MgSO_4$ and purified by Preparative thin-layer chromatography (PTLC) separations ((EtOAc/*n*-hexane = 1:8) to afford **7** as yellow solid (259 mg, 0.51 mmol, 84%). R_f = 0.28 (EtOAc/n-hexane = 1:4). **^{1}H NMR** (400 MHz, $CDCl_3$) δ 8.27 (d, *J* = 9.3 Hz, 4H), 7.56 (d, *J* = 7.1 Hz, 2H), 7.51 (d, *J* = 8.3 Hz, 2H), 7.51 (d, *J* = 8.3 Hz, 2H), 7.46 (d, *J* = 7.3 Hz, 2H), 7.41 (d, *J* = 9.3 Hz, 4H),7.36 (t, *J* = 7.3 Hz, 1H), 7.31 (d, *J* = 8.4 Hz, 2H), 4.55 (s, 2H). **^{13}C{^{1}H} NMR** (101 MHz, $CDCl_3$) δ 154.5 (s), 146.4, 140.6, 137.3, 129.0, 128.0, 127.6, 127.4, 127.1, 125.8, 122.4, 49.6. **HRMS (ESI,** m/z**):** $[M - H]^-$ Calcd for $C_{25}H_{18}N_3O_7S^-$: 504.0871, Found 504.0881. Melting point: 139.9-140.6 °C.

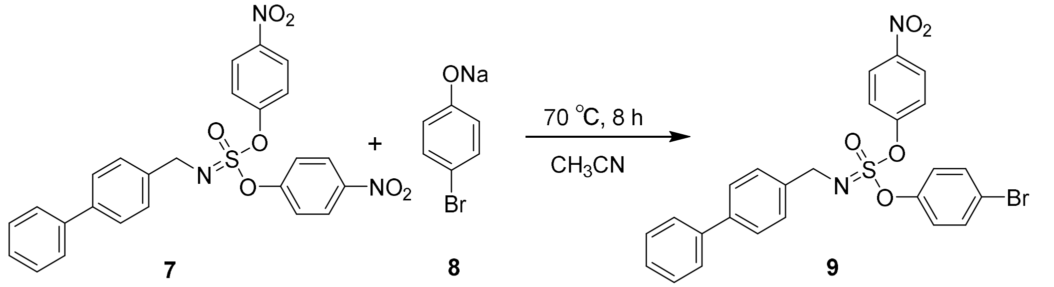

Scheme S7. Synthesis of compound **9**.

Compound 9: 7 (20.0 mg, 0.039 mmol, 1.0 equiv.) was dissolved in anhydrous CH_3CN (2 mL) under argon protection. Then sodium *para*-bromide phenolate (76.0 mg, 0.39 mmol, 10.0 equiv.) was added to the solution. The reaction mixture was stirred for 8 h at 70 °C. The reaction was quenched by adding water (2 mL), extracted with CH_2Cl_2 (2 × 15 mL), dried with anhydrous $MgSO_4$ and the crude compound was purified by preparative TLC plate (n-hex:CH_2Cl_2: MeOH = 70:30:2) to give compound **9** as white solid (17.0 mg, 0.032 mmol, 81%). R_f = 0.40 (EtOAc/n-hexane = 1:4).

^{1}H NMR (400 MHz, $CDCl_3$) δ 8.24 (d, *J* = 9.1 Hz, 2H), 7.61 – 7.55 (m, 2H), 7.52 (dd, *J* = 8.6, 3.0 Hz, 4H), 7.45 (t, *J* = 7.7 Hz, 2H), 7.41 – 7.33 (m, 3H), 7.32 (d, *J* = 8.0 Hz, 2H), 7.16(d, *J* = 8.9 Hz, 2H), 4.54 (s, 2H). **^{13}C{^{1}H} NMR** (101 MHz, $CDCl_3$) δ 154.8, 149.4, 146.2, 140.8, 140.5, 137.7, 133.2, 129.0, 128.0, 127.5, 127.3, 127.2, 125.7, 123.5, 122.4, 121.0, 49.5. **HRMS (ESI,** m/z**)**: $[M + H]^+$ Calcd for $C_{25}H_{20}BrO_5N_2S^+$: 539.0271 $[M]^+$, Found: 539.0257.

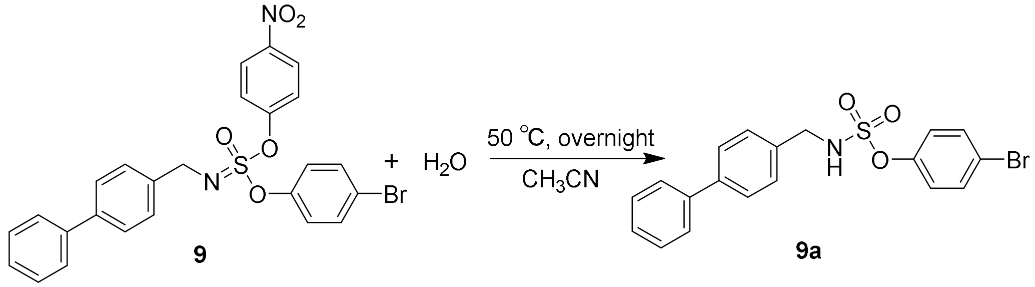

9 **9a**

Scheme S8. Hydrolysis of compound **9**.

Compound 9a: 9 (8.5 mg, 0.0157 mmol, 1.0 equiv.) was dissolved in CH_3CN (1 mL) under argon protection. Then water (0.5 mL) was added to the solution. The reaction mixture was stirred at 50 °C for overnight. The reaction was extracted with CH_2Cl_2 (3 × 1 mL), dried with anhydrous $MgSO_4$ and the crude compound was purified by preparative TLC plate (n-hex:CH_2Cl_2: MeOH = 70:30:2) to give compound **9a** as brown solid (6.04 mg, 0.014 mmol, 92%). R_f = 0.38 (EtOAc/n-hexane = 1:4). **^{1}H NMR** (400 MHz, $CDCl_3$) δ 7.56 (d, *J* = 8.4 Hz, 4H), 7.48 – 7.40 (m, 4H), 7.39 – 7.31 (m, 3H), 7.07 (d, *J* = 9.0 Hz, 2H), 4.39 (d, *J* = 6.0 Hz, 2H). **^{13}C{^{1}H} NMR** (101 MHz, $CDCl_3$) δ 149.2, 141.2, 140.4, 134.9, 132.8, 128.9, 128.6, 127.5, 127.0, 123.7, 120.2, 116.5, 47.8. **HRMS (ESI,** m/z**)**: $[M - H]^-$ Calcd: for $C_{19}H_{15}BrNO_3S^-$: 417.9930, Found: 417.9954.

3. Kinetic Studies of first & second group in S(VI) for SuFEx and SuPhenEx reactions

3.1 Determination of molar extinction coefficients of compounds

All compounds were made as a solution with concentration of ca. 0.25 ×10^{-4} mol/L in 10 mL solvent (compound **1**, **3**, **4**, **5**, and **6** were dissolved in *n*-hexane/isopropanol = 95:5, and compound **7**, **9**, and **9a** were dissolved in *n*-hexane/isopropanol = 90:10. The absorption spectra were obtained at from 200 - 800 nm, with a scan rate of 600 nm/min. From the spectra, λ_{max} was obtained, and ε_{max} and ε_{254} were calculated using Beer-Lambert Law (**equation 1** below) and listed in **Table S1**.

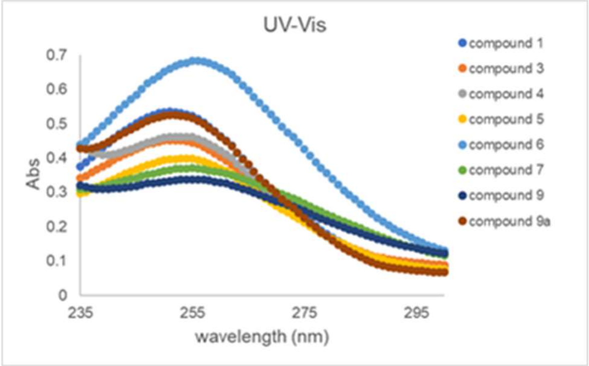

Figure S1. UV-Vis adsorption spectra of compound **1**, **3** ,**4** ,**5** ,**6** ,**7** ,**9** and **9a**.

$$A = \varepsilon c l \quad (1)$$

where A is the UV-Vis absorption, ε is the molar extinction coefficient, c is the concentration of the compound and l is the distance that the light travels through the material (1 cm).

Table S1. Maximum molar extinction coefficients and molar extinction coefficients at 254 nm of compound **1**, **3**, **4**, **5**, **6**, **7**, **9** and **9a**.

Compound	ε_{max} L(mol×cm)$^{-1}$	λ_{max} (nm)	ε_{254} L(mol×cm)$^{-1}$	Concentration (mol/L)
**1**	2.15 ×10^4	251	2.12 ×10^4	0.249 ×10^{-4}
**3**	1.82 ×10^4	252	1.80 ×10^4	0.249 ×10^{-4}
**4**	1.87 ×10^4	253	1.86 ×10^4	0.248 ×10^{-4}
**5**	1.64 ×10^4	255	1.61 ×10^4	0.247 ×10^{-4}
**6**	2.84 ×10^4	256	2.81 ×10^4	0.241 ×10^{-4}
**7**	2.21 ×10^4	255	2.20 ×10^4	0.168 ×10^{-4}
**9**	1.54 ×10^4	255	1.53 ×10^4	0.220 ×10^{-4}
**9a**	2.11 ×10^4	251	2.10 ×10^4	0.248 ×10^{-4}

The maximum UV absorption was for all compounds under study to be in the range 251-256 nm, suggesting that their UV absorptions were all dominated by the substituted

biphenyl moiety. The HPLC detection used for all kinetics was at 254 nm, i.e. close to the maximum for all compounds under study.

3.2 Determination of reaction constants of first & second group in S(VI) for SuFEx and SuPhenEx reactions at different temperatures

The reaction rates of SuFEx and SuPhenEx reactions were studied at different temperatures. For SuFEx reaction, as a representative reaction, the starting materials iminosulfur oxydifluorides (R^1NSOF_2) **1** and **3** were reacted with 4-trifluoromethylphenol (compound **2**) separately. Reaction rates were determined under pseudo-first-order conditions, whereby 4-trifluoromethylphenol was added in 10-fold excess. Reactions were monitored using chiral HPLC. The kinetic measurements of the replacement of the first F atom in compound **1** with the concentration of 0.5 mM were performed at 16 °C, 23 °C, 31 °C, 38 °C and 47 °C. The reaction of the second F moiety is much slower, so kinetic measurements were performed at 40 °C, 47 °C, 55 °C, 62 °C and 70 °C with higher concentration (30.0 mM) of compound **3**.

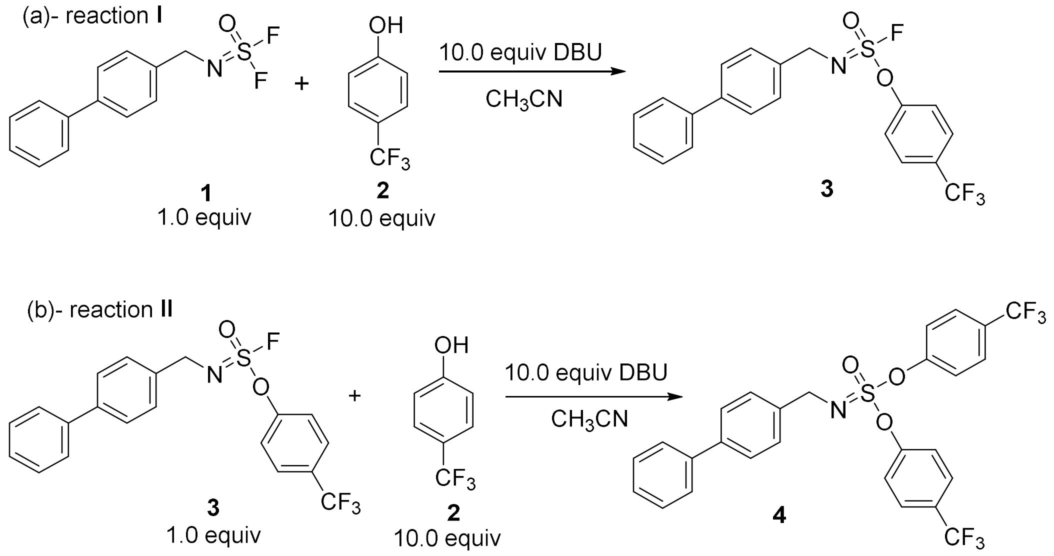

Scheme S9. Model SuFEx reactions employed for kinetic studies, (a) reaction **I**-first F SuFEx reaction and (b) reaction **II**-second F SuFEx reaction.

3.2.1 Equipment and procedures

Mother liquor: Compound 1 （41.4 mg, 0.15 mmol） was dissolved in CH_3CN (30.98 ml); 4-trifluoromethylphenol (158.8 mg, 0.98 mmol) was dissolved in CH_3CN (19.59 ml).

In a 2-mL EP tube, the starting material **1** (100 μL, 0.0005 mmol, 1.0 equiv.) and phenol **2** (10.0 equiv., 100 μL, 0.005 mmol) were dissolved in 0.8 ml of CH_3CN and cooled to 16 °C for 30 min in thermostatic metal bath. In an EP tube, DBU was cooled to 16 °C for 30 min at the same time. DBU (10.0 equiv., 0.75 μL) was transferred to the starting material vial using micropipette to start the reaction. Samples (each time 20 μl) were collected at particular time intervals. Each sample was quenched by the addition of *iso*-

propanol (0.15 mL). Obtained samples were analyzed by chiral HPLC (*n*-hexane/isopropanol = 95:5, flow rate 0.5 mL/min, detector wavelength 254 nm in CHIRALPAK®IA column).

For the reaction rate of the first SuFEx reaction (**Scheme S9**, reaction **I**), the peaks of the starting material **1** (8.0 min), the corresponding phenol **2** (15.6 min) and the product [compound **3** (10.7 & 12.2 min)], were used; pseudo– first order kinetics were used – see section **3.3**.

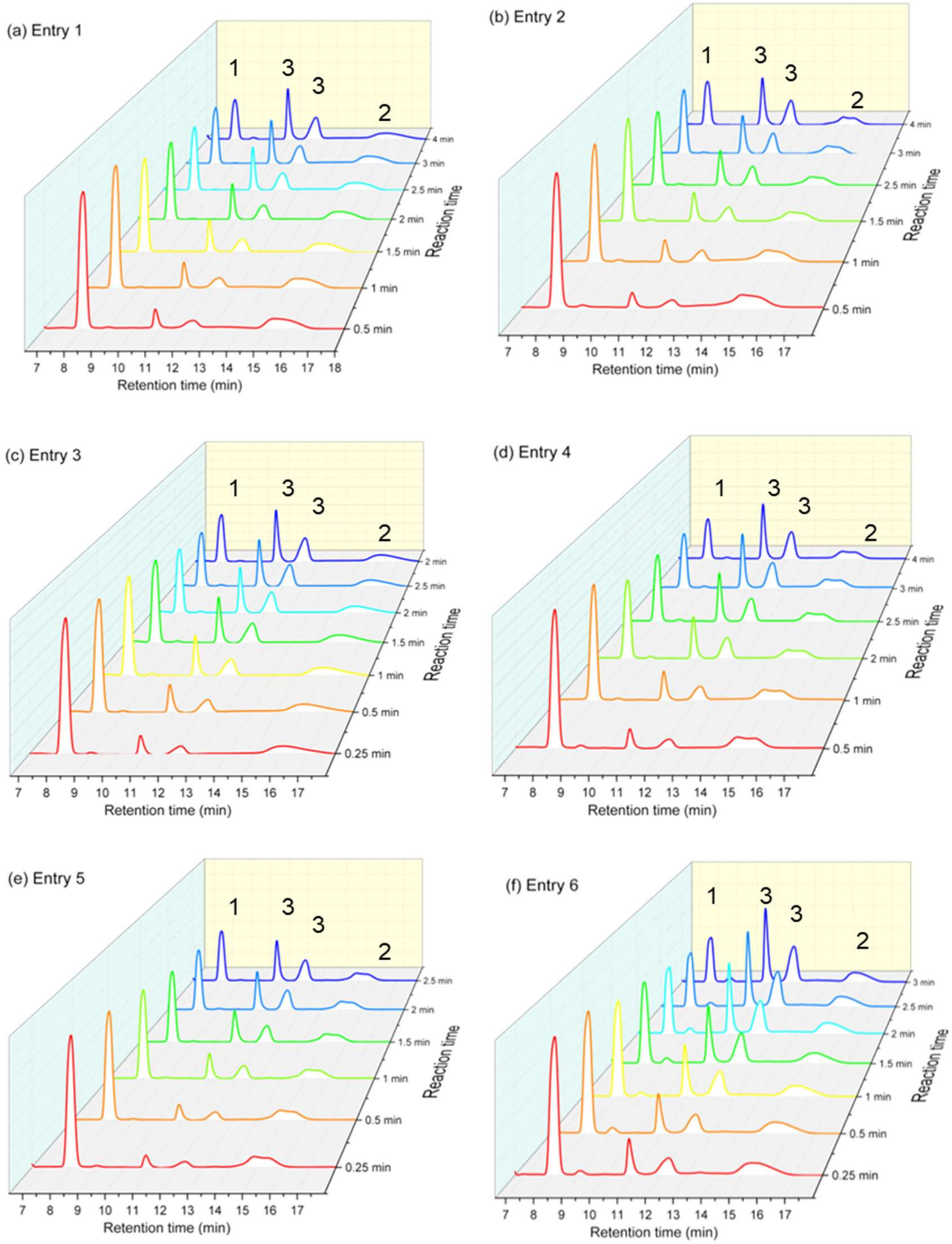

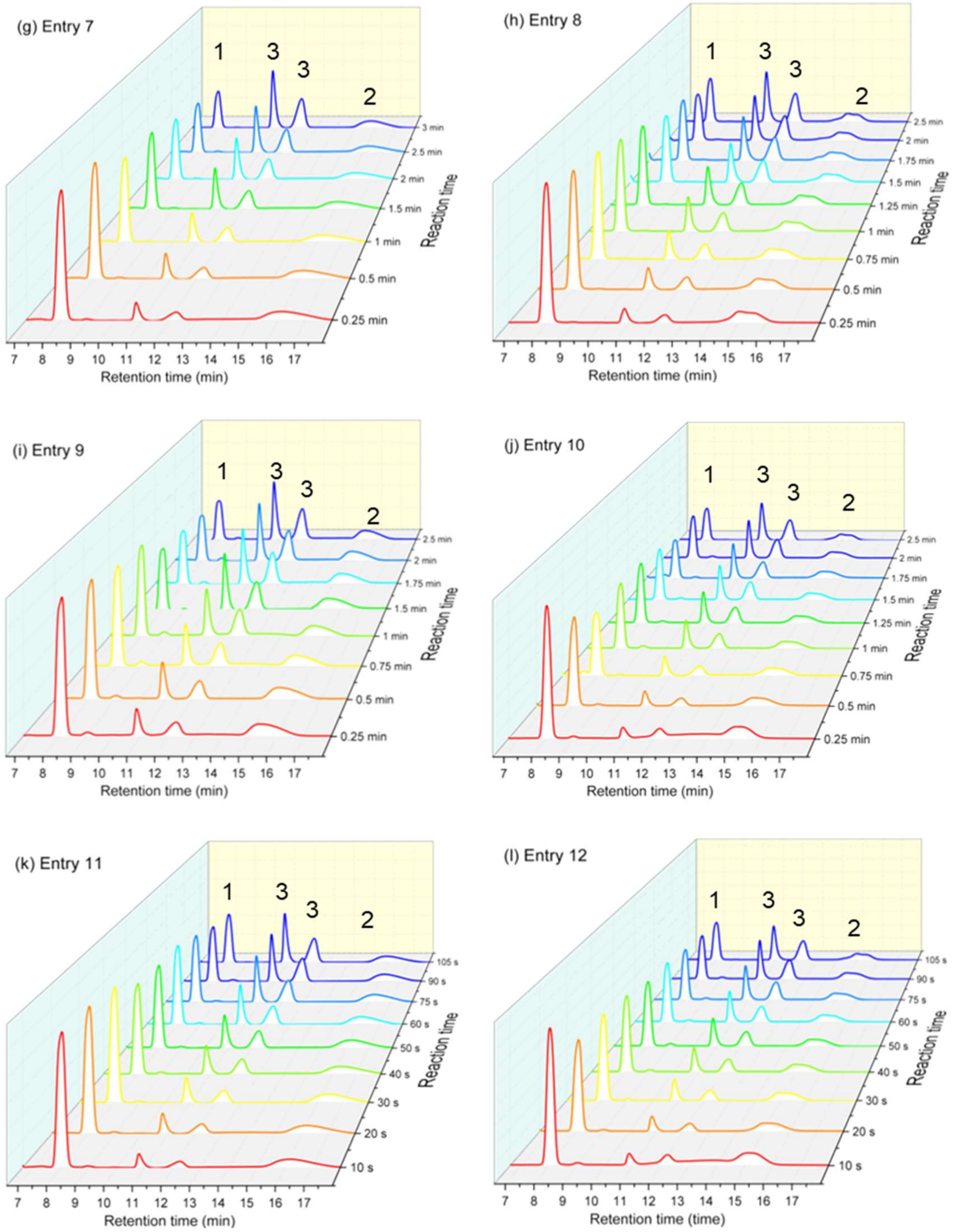
(g) Entry 7
1
3
3
2
3 min
2.5 min
2 min
1.5 min
1 min
0.5 min
0.25 min
Reaction time
7 8 9 10 11 12 13 14 15 16 17
Retention time (min)
(h) Entry 8
1
3
3
2
2.5 min
2 min
1.75 min
1.5 min
1.25 min
1 min
0.75 min
0.5 min
0.25 min
Reaction time
7 8 9 10 11 12 13 14 15 16 17
Retention time (min)
(i) Entry 9
1
3
3
2
2.5 min
2 min
1.75 min
1.5 min
1 min
0.75 min
0.5 min
0.25 min
Reaction time
7 8 9 10 11 12 13 14 15 16 17
Retention time (min)
(j) Entry 10
1
3
3
2
2.5 min
2 min
1.75 min
1.5 min
1.25 min
1 min
0.75 min
0.5 min
0.25 min
Reaction time
7 8 9 10 11 12 13 14 15 16 17
Retention time (min)
(k) Entry 11
1
3
3
2
105 s
90 s
75 s
60 s
50 s
40 s
30 s
20 s
10 s
Reaction time
7 8 9 10 11 12 13 14 15 16 17
Retention time (min)
(l) Entry 12
1
3
3
2
105 s
90 s
75 s
60 s
50 s
40 s
30 s
20 s
10 s
Reaction time
7 8 9 10 11 12 13 14 15 16 17
Retention time (time)

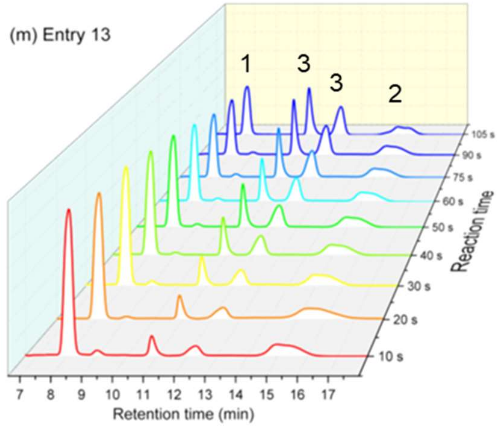

Figure S2. HPLC data used for the rate determination of the SuFEx reaction of the first F group in compound **1** with 4-trifluoromethylphenol at (a-b) 16 °C, (c-d) 23 °C (e-g) 31 °C, (h-j) 38 °C, (k-m) 47 °C (see **Scheme S9**, reaction **I**).

Mother liquor: Compound 3（153.5 mg 0.37 mmol）was dissolved in CH_3CN (5 ml); 4-trifluoromethylphenol (608 mg, 3.75 mmol) was dissolved in CH_3CN (5 ml). In a 5-mL round-bottom flask equipped with an oil bath, the starting material **3** (400 µL, 0.003 mmol, 1.0 equiv) and phenol-compound **2** (400 µL, 0.03 mmol, 10.0 equiv) were dissolved in 0.2 ml of CH_3CN and heated to 40 °C for 30 min. In a 5-mL round-bottom flask, DBU was heated to 40 °C for 30 min at the same time. DBU (10.0 equiv,44.8 µL) was transferred to the starting material flask using micropipette to start the reaction. Samples (each time 10 µl) were collected at particular time intervals. Each sample was quenched by the addition of *iso*-propanol (1 mL). Obtained samples were analyzed by chiral HPLC (*n*-hexane/*iso*-propanol = 95:5, flow rate 0.5 mL/min, detector wavelength 254 nm in CHIRALPAK®IA column). For the reaction rate of the second SuFEx reaction (**Scheme S9**, reaction **II**), the peaks for the starting material **3** (11.5 min & 12.8 min) and corresponding phenol (15.4 min) and product **4** (17.1 min) were used. To determine the rate constant, the method used for pseudo– first order kinetics was shown in section **3.3**.

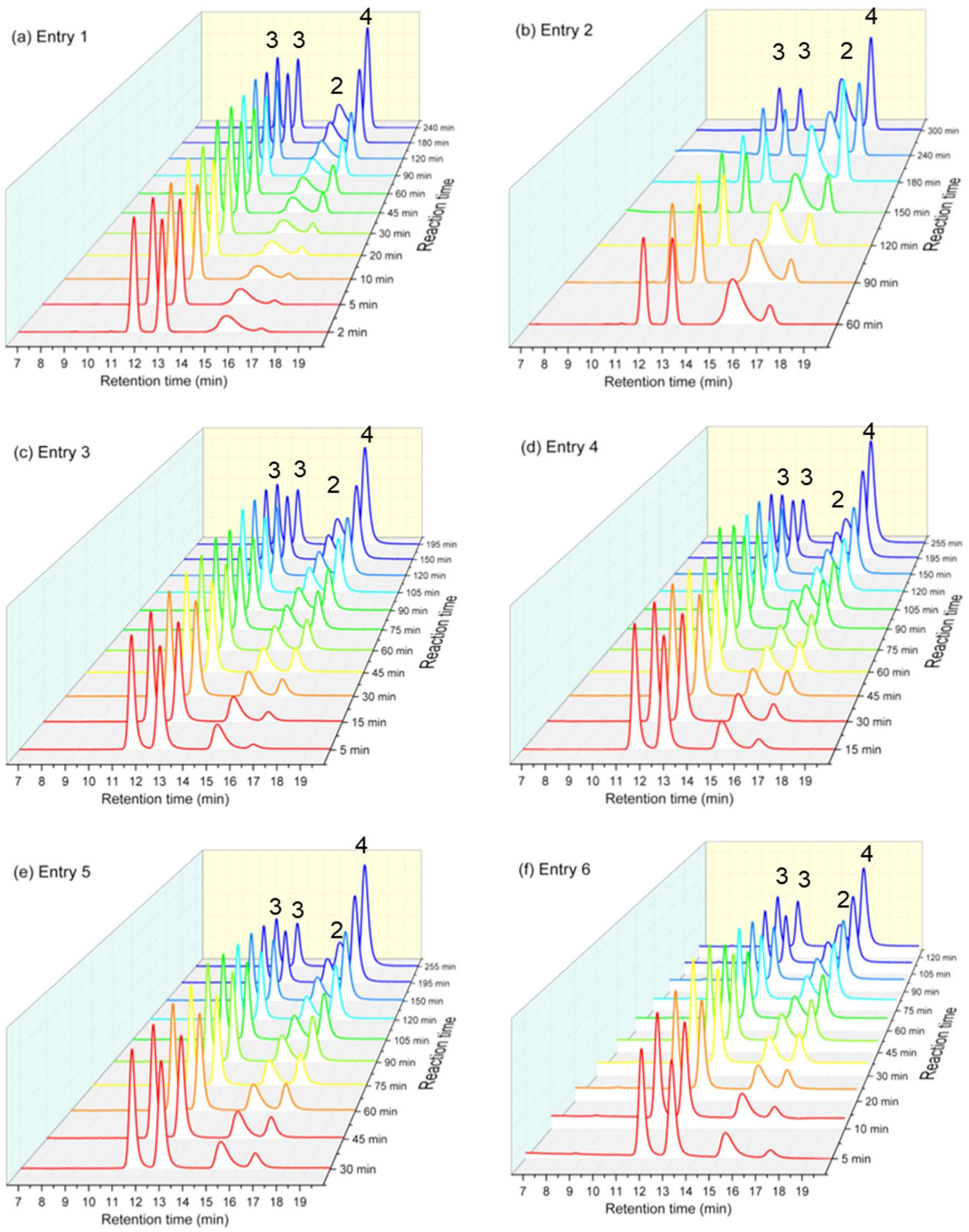
(a) Entry 1
3 3 2 4
240 min 180 min 120 min 90 min 60 min 45 min 30 min 20 min 10 min 5 min 2 min
Reaction time
7 8 9 10 11 12 13 14 15 16 17 18 19
Retention time (min)
(b) Entry 2
3 3 2 4
300 min 240 min 180 min 150 min 120 min 90 min 60 min
Reaction time
7 8 9 10 11 12 13 14 15 16 17 18 19
Retention time (min)
(c) Entry 3
3 3 2 4
195 min 150 min 120 min 105 min 90 min 75 min 60 min 45 min 30 min 15 min 5 min
Reaction time
7 8 9 10 11 12 13 14 15 16 17 18 19
Retention time (min)
(d) Entry 4
3 3 2 4
255 min 195 min 150 min 120 min 105 min 90 min 75 min 60 min 45 min 30 min 15 min
Reaction time
7 8 9 10 11 12 13 14 15 16 17 18 19
Retention time (min)
(e) Entry 5
3 3 2 4
255 min 195 min 150 min 120 min 105 min 90 min 75 min 60 min 45 min 30 min
Reaction time
7 8 9 10 11 12 13 14 15 16 17 18 19
Retention time (min)
(f) Entry 6
3 3 2 4
120 min 105 min 90 min 75 min 60 min 45 min 30 min 20 min 10 min 5 min
Reaction time
7 8 9 10 11 12 13 14 15 16 17 18 19
Retention time (min)

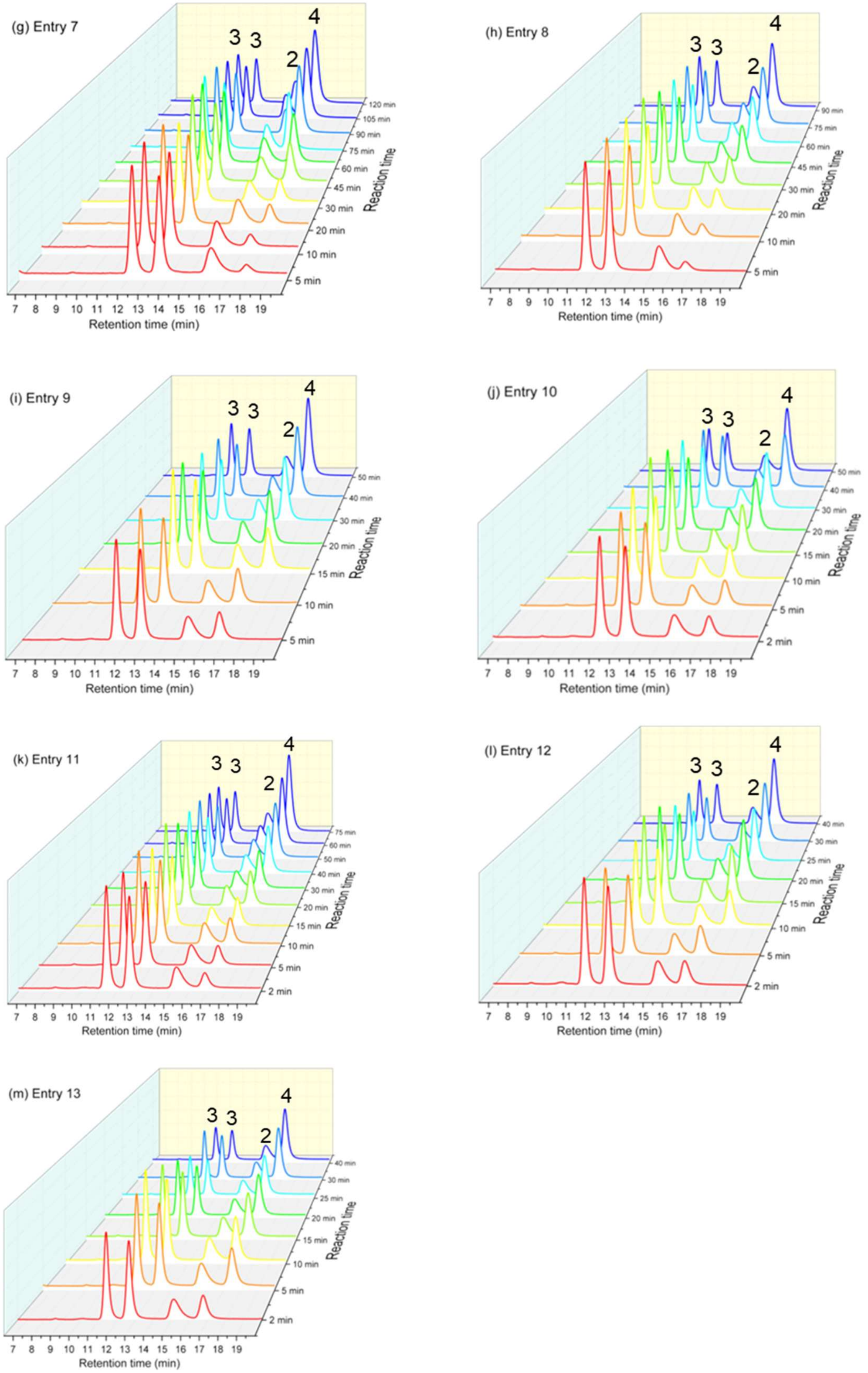

Figure S3. HPLC data used for the rate determination of the SuFEx reaction of the second F (fluride) moiety in compound **3** with 4-trifluoromethylphenol at (a-b) 40 ℃, (c-e) 47 ℃, (f-h) 55 ℃, (i-k) 62 ℃, (l-m) 70 ℃ (see scheme **S9**, reaction **II**).

3.3 Rate constant determination

Equation 2 describes the second-order rate of the reaction studied($mol^{-1}\cdot s^{-1}$), where [1] is the concentration of starting material **1** and [2] is the concentration of 4-trifluoromethylphenol, and k_2 is the second-order rate constant ($M^{-1}s^{-1}$). Since, the amount of phenol (10 equivalents) is in greatly surplus compared to the amount of **1**, it is assumed to be essentially constant throughout the entire kinetics experiment (**equation 3**). Thus, the SuFEx reaction **I** can be reduced to pseudo–first order kinetics, with rate constant, *k'*. Therefore, from the plot of ln $[(I_\infty - I_t)/(I_\infty - I_0)]$ versus time, the pseudo first–order rate constant (*k'*) can be obtained directly from the slope, as shown in **equation 4**.

$$v = k_2\,[1][2] = \frac{d[1]}{dt} \qquad (2)$$

$$v = k'[1]\ \text{(since [2] » [1])} \qquad (3)$$

$$\ln[\frac{I\infty - It}{I\infty - I0}] = k't \qquad (4)$$

So: $k' = k_2\,[2]$,

$$k_2 = \frac{k\prime}{[2]}$$

3.3.1 Rate determination of SuFEx reaction (Scheme S9, reaction I and reaction II) at different temperature

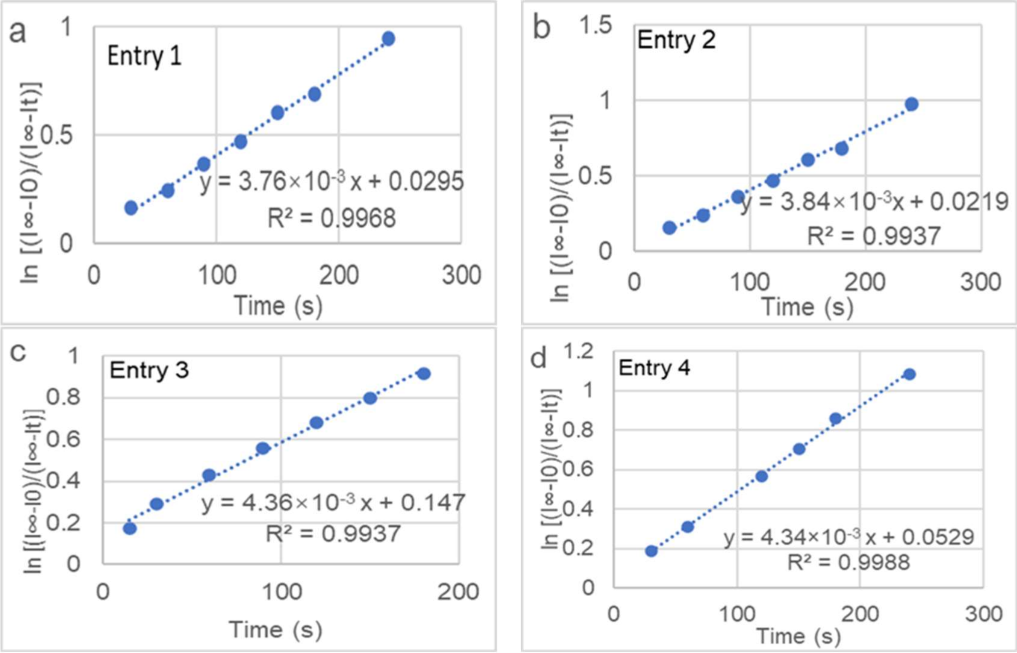

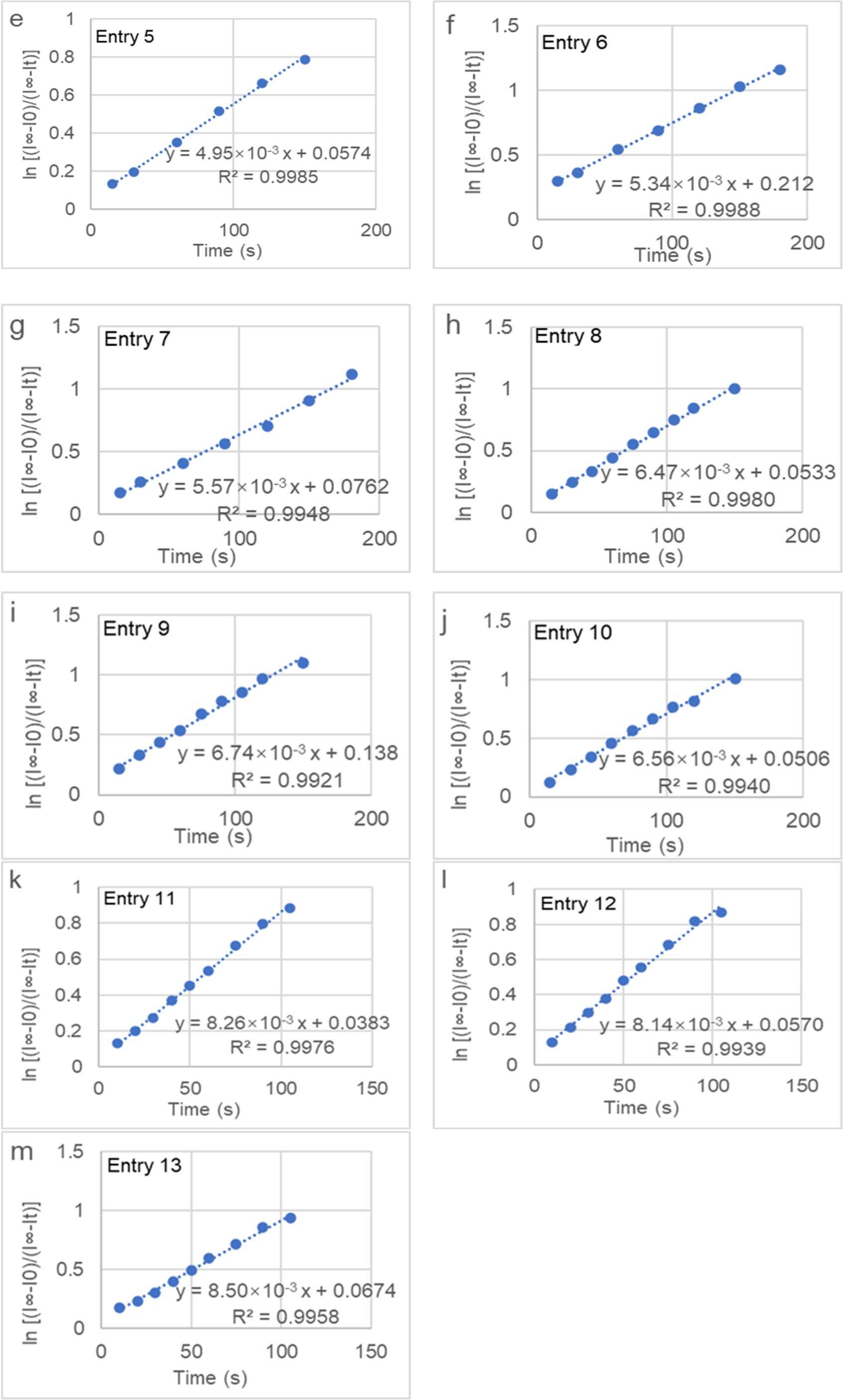
e
Entry 5
y = 4.95×10-3 x + 0.0574
R² = 0.9985
ln [(I∞-I0)/(I∞-It)]
Time (s)
f
Entry 6
y = 5.34×10-3 x + 0.212
R² = 0.9988
ln [(I∞-I0)/(I∞-It)]
Time (s)
g
Entry 7
y = 5.57×10-3 x + 0.0762
R² = 0.9948
ln [(I∞-I0)/(I∞-It)]
Time (s)
h
Entry 8
y = 6.47×10-3 x + 0.0533
R² = 0.9980
ln [(I∞-I0)/(I∞-It)]
Time (s)
i
Entry 9
y = 6.74×10-3 x + 0.138
R² = 0.9921
ln [(I∞-I0)/(I∞-It)]
Time (s)
j
Entry 10
y = 6.56×10-3 x + 0.0506
R² = 0.9940
ln [(I∞-I0)/(I∞-It)]
Time (s)
k
Entry 11
y = 8.26×10-3 x + 0.0383
R² = 0.9976
ln [(I∞-I0)/(I∞-It)]
Time (s)
l
Entry 12
y = 8.14×10-3 x + 0.0570
R² = 0.9939
ln [(I∞-I0)/(I∞-It)]
Time (s)
m
Entry 13
y = 8.50×10-3 x + 0.0674
R² = 0.9958
ln [(I∞-I0)/(I∞-It)]
Time (s)

Figure S4. Calculation of the rates of the replacement of the first F in compound **1** (**Scheme S9**, reaction **I**) at different temperatures in CH_3CN. Conversions are obtained from HPLC at (a-b) 16 °C, (c-d) 23 °C (e-g) 31 °C, (h-j) 38 °C, (k-m) 47 °C.

Table S2. Reaction rates of reaction **I** (**Scheme S9**) at different temperatures

Entry	Temperature	k'(s^{-1})	k_2($M^{-1}s^{-1}$)	Average rate with standard deviation/($M^{-1}s^{-1}$)
1	16 °C	0.00376	0.775	0.783 ± 0.008
2	16 °C	0.00384	0.791	
3	23 °C	0.00436	0.899	0.897 ± 0.002
4	23 °C	0.00434	0.895	
5	31 °C	0.00495	1.02	
6	31 °C	0.00534	1.10	1.09 ± 0.06
7	31 °C	0.00557	1.15	
8	38 °C	0.00647	1.33	
9	38 °C	0.00674	1.39	1.36 ± 0.02
10	38 °C	0.00656	1.35	
11	47 °C	0.00826	1.70	
12	47 °C	0.00814	1.68	1.71 ± 0.03
13	47 °C	0.00850	1.75	

(When the conversion rate of compound **1** reaches to 60%, the initial concentration of phenol is 0.005 M/L, and the final concentration is 0.0047 M/L. For calculation of the second-order rate constant, the average concentration, 0.00485 M/L, was used)

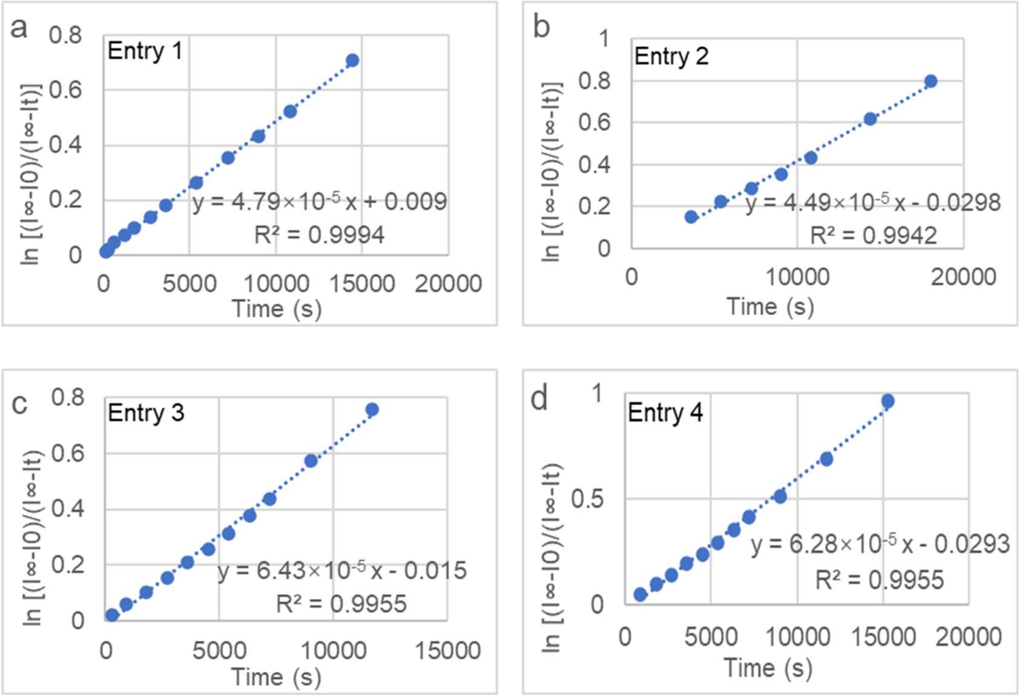

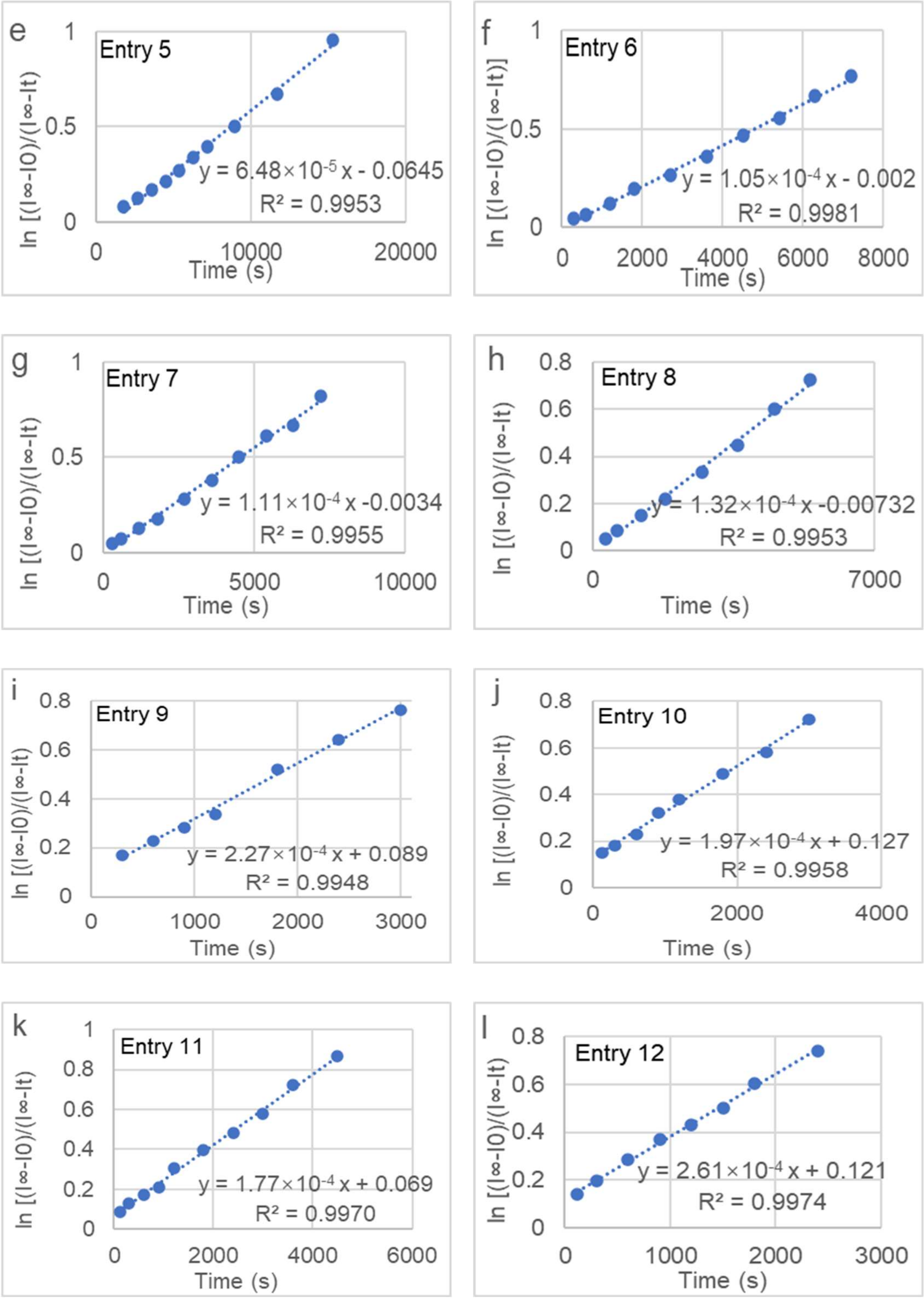
e
Entry 5
y = 6.48×10-5 x - 0.0645
R² = 0.9953
ln [(I∞-I0)/(I∞-It)]
Time (s)
f
Entry 6
y = 1.05×10-4 x - 0.002
R² = 0.9981
ln [(I∞-I0)/(I∞-It)]
Time (s)
g
Entry 7
y = 1.11×10-4 x -0.0034
R² = 0.9955
ln [(I∞-I0)/(I∞-It)
Time (s)
h
Entry 8
y = 1.32×10-4 x -0.00732
R² = 0.9953
ln [(I∞-I0)/(I∞-It)
Time (s)
i
Entry 9
y = 2.27×10-4 x + 0.089
R² = 0.9948
ln [(I∞-I0)/(I∞-It)
Time (s)
j
Entry 10
y = 1.97×10-4 x + 0.127
R² = 0.9958
ln [(I∞-I0)/(I∞-It)
Time (s)
k
Entry 11
y = 1.77×10-4 x + 0.069
R² = 0.9970
ln [(I∞-I0)/(I∞-It)
Time (s)
l
Entry 12
y = 2.61×10-4 x + 0.121
R² = 0.9974
ln [(I∞-I0)/(I∞-It)
Time (s)

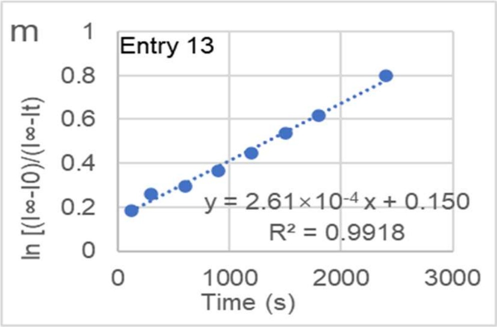

Figure S5. Calculation of the rates of replacement of the second F (fluride), *i.e.*, in compound **3** (**Scheme S9**, reaction **II**) at different temperatures in CH_3CN. Conversions are obtained from HPLC at (a-b) 40 °C, (c-e) 47 °C, (f-h) 55 °C, (i-k) 62 °C, (l-m) 70 °C.

Table S3. Parameters for reaction rates of reaction **II** (**Scheme S9**) at different temperatures

Entry	Temperature	$k'(s^{-1})$	$k_2(M^{-1}s^{-1})$	Average rate with standard deviation/$(M^{-1}s^{-1})$
1	40 °C	4.79×10^{-5}	1.64×10^{-4}	$(1.59 \pm 0.05)\times10^{-4}$
2	40 °C	4.49×10^{-5}	1.54×10^{-4}	
3	47 °C	6.43×10^{-5}	2.20×10^{-4}	$(2.19 \pm 0.03)\times10^{-4}$
4	47 °C	6.28×10^{-5}	2.15×10^{-4}	
5	47 °C	6.48×10^{-5}	2.21×10^{-4}	
6	55 °C	1.05×10^{-4}	3.60×10^{-4}	$(3.98 \pm 0.41)\times10^{-4}$
7	55 °C	1.11×10^{-4}	3.80×10^{-4}	
8	55 °C	1.32×10^{-4}	4.53×10^{-4}	
9	62 °C	2.27×10^{-4}	7.77×10^{-4}	$(6.85 \pm 0.70)\times10^{-4}$
10	62 °C	1.97×10^{-4}	6.73×10^{-4}	
11	62 °C	1.77×10^{-4}	6.05×10^{-4}	
12	70 °C	2.61×10^{-4}	8.92×10^{-4}	$(8.92\pm0.01)\times10^{-4}$
13	70 °C	2.61×10^{-4}	8.91×10^{-4}	

(When the conversion rate of compound **4** reaches to 50%, the initial concentration of phenol is 0.3 M/L, and the final concentration is 0.285 M/L. For calculation of the second-order rate constant, the average concentration, 0.2925 M/L was used.)

3.4 Determination of activation enthalpy from the reaction rates (reaction I & II, Scheme S9) using Eyring Equation.

The reaction rates measured at different temperature were used to calculate the activation

enthalpy of the SuFEx reaction using Eyring equation. From the plot of ln (*k*/T) verses 1/T (1/K),

-ΔH‡/R could be obtained from the slope value as shown in the below equation.

$$Eyring\ \ equation = \ln\frac{k}{T} = -\frac{\Delta H^{\ddagger}}{R}\frac{1}{T} + \ln\frac{k_B}{h} + \frac{\Delta S^{\ddagger}}{R} \qquad (5)$$

The equation also could be given as: $k = \frac{k_B T}{h} e^{-(\frac{\Delta H\ddagger}{RT})} e^{(\frac{\Delta S\ddagger}{R})}$ (6)

Table S4. Parameters of reaction **I** (**Scheme S9**) for use in Eyring equation.

Temperature K	1/Temperature (1/K)	*k*	*k*/T	ln *k*/T
320.15	3.12×10^{-3}	1.71	5.35×10^{-3}	-5.231
311.15	3.21×10^{-3}	1.36	4.37×10^{-3}	-5.434
304.15	3.29×10^{-3}	1.09	3.58×10^{-3}	-5.631
296.15	3.38×10^{-3}	0.897	3.03×10^{-3}	-5.799
289.15	3.46×10^{-3}	0.783	2.71×10^{-3}	-5.911

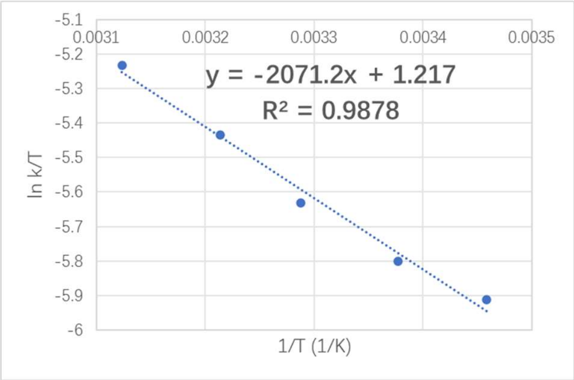

Figure S6. Eyring plot for the first SuFEx reaction (**Scheme S9**, reaction **I**) to determine the activation enthalpy.

Slope = (-ΔH‡)/R

-2071.2 = -ΔH‡/8.314 J M^{-1} K^{-1}

ΔH‡ = 2071.2 K × 8.314 J M^{-1} K^{-1} = 17.22 kJ M^{-1} = 4.11 kcal M^{-1}

Therefore, an activation enthalpy was calculated to be 4.11 kcal M^{-1} for the first F SuFEx reaction (**Scheme S9**, reaction **I**). Further, the Eyring equation was used to determine activation entropy change for first F SuFEx reaction. k_B is the Boltzmann's constant (1.381 × 10^{-23} J/K); h is Planck's constant (6.626 × 10^{-34} Js).

$\ln(k_B/h) = 23.76$
$1.217 = 23.76 + \Delta S^‡/R$
$\Delta S^‡/ 8.314\ J\ M^{-1}\ K^{-1} = -22.54$
$\Delta S^‡ = -22.54 \times 8.314\ J\ M^{-1}\ K^{-1} = -0.187\ kJ\ M^{-1}\ K^{-1} = -0.0448\ kcal\ M^{-1}\ K^{-1}$
$\Delta G^‡ = \Delta H^‡ - T\Delta S^‡$
Here $\Delta G_{25}^‡$ ($\Delta G^‡$ at 25 °C) could be calculated as

$\Delta G_{25}^‡ = 4.11\ kcal\ M^{-1} – 298.15\ K \times -0.0448\ kcal\ M^{-1}\ K^{-1} = 17.47\ kcal\ M^{-1}$

Therefore $\Delta G_{25}^‡$ was found to be 17.47 kcal M^{-1} and $\Delta S^‡$ was found to be -0.0448 kcal $M^{-1}\ K^{-1}$.

Table S5. Parameters of reaction **II** (**Scheme S9**) for use in the Eyring equation.

Temperature K	1/Temperature (1/K)	*k*	*k*/T	ln *k*/T
313.15	3.19×10^{-3}	1.59×10^{-4}	5.06×10^{-7}	-14.49
320.15	3.12×10^{-3}	2.19×10^{-4}	6.83×10^{-7}	-14.20
328.15	3.05×10^{-3}	3.98×10^{-4}	1.21×10^{-6}	-13.62
335.15	2.98×10^{-3}	6.85×10^{-4}	2.04×10^{-6}	-13.10
343.15	2.91×10^{-3}	8.92×10^{-4}	2.60×10^{-6}	-12.86

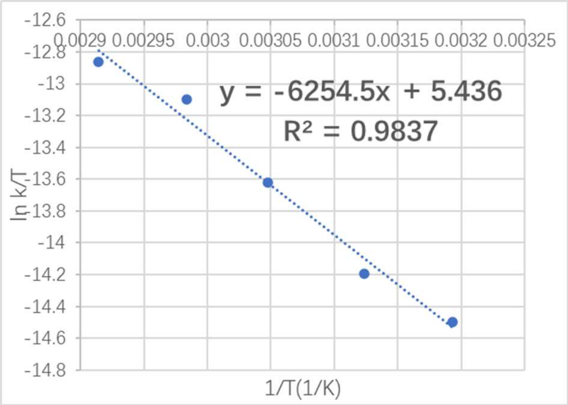

Figure S7. Eyring plot for the second SuFEx reaction (Scheme **S9**, reaction **II**) to determine the activation enthalpy.

Slope = $(-\Delta H^‡)/R$
$-6254.5 = -\Delta H^‡/8.314\ J\ M^{-1}\ K^{-1}$
$\Delta H^‡ = 6254.5\ K \times 8.314\ J\ M^{-1}\ K^{-1} = 52.00\ kJ\ M^{-1} = 12.42\ kcal\ M^{-1}$
Therefore, an activation enthalpy was calculated to be 12.42 kcal M^{-1} for the second F SuFEx reaction (**Scheme S9**, reaction **II**). Further, the Eyring equation was used to determine activation entropy change for first F SuFEx reaction. k_B is the Boltzmann's constant (1.381×10^{-23} J/K); h is Planck's constant (6.626×10^{-34} J/s).

$\ln(k_B/h) = 23.76$

$5.436 = 23.76 + \Delta S^{\ddagger}/R$

$\Delta S^{\ddagger}/ 8.314\ J\ M^{-1}\ K^{-1} = -18.32$

$\Delta S^{\ddagger} = -18.32 \times 8.314\ J\ M^{-1}\ K^{-1} = -0.152\ kJ\ M^{-1}\ K^{-1} = -0.0363\ kcal\ M^{-1}\ K^{-1}$

$\Delta G^{\ddagger} = \Delta H^{\ddagger} - T\Delta S^{\ddagger}$

Here $\Delta G_{25}^{\ddagger}$ ($\Delta G^{\ddagger}$ at 25 °C) could be calculated as

$\Delta G_{25}^{\ddagger} = 12.42\ kcal\ M^{-1} – 298.15\ K \times -0.0363\ kcal\ M^{-1}\ K^{-1} = 23.24\ kcal\ M^{-1}$

Therefore $\Delta G_{25}^{\ddagger}$ was found to be 23.24 kcal M^{-1} and $\Delta S^{\ddagger}$ was found to be -0.0363 kcal $M^{-1}\ K^{-1}$.

4. Competition between SuFEx and SuPhenEx reaction

The competition between SuFEx and SuPhenEx reaction was studied using the starting material **5** (1.0 equiv) reacted with 4-trifluoromethylphenol (10.0 equiv). Reactions were monitored using chiral HPLC. Kinetic measurements were performed at 41 °C, 48 °C, 56 °C, 63 °C and 71 °C.

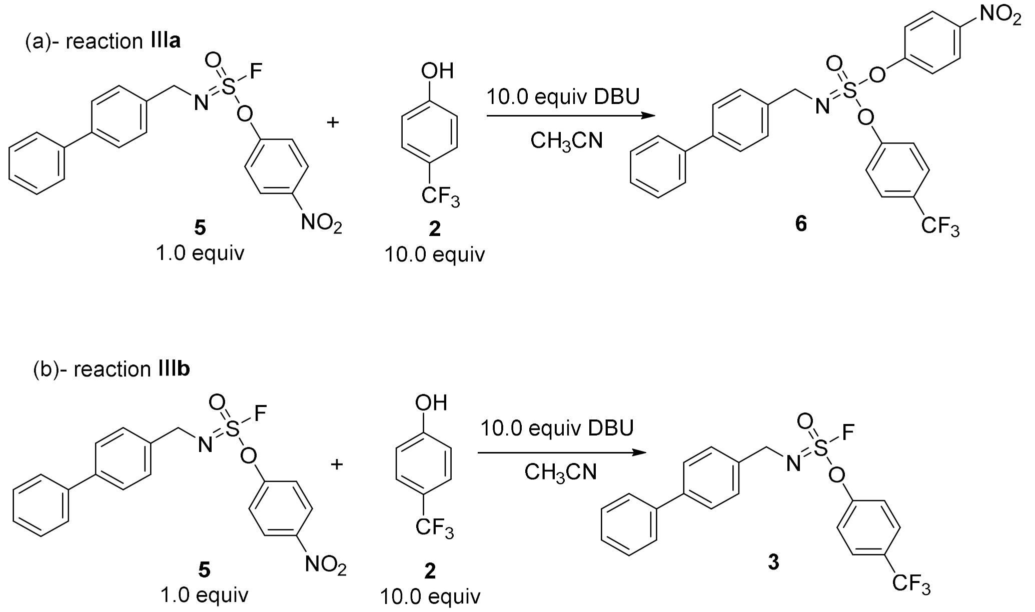

Scheme S8. Model reactions employed for kinetic studies for competitive SuFEx and SuPhenEx reactions, (a) reaction **IIIa-** SuFEx reaction and (b) reaction **IIIb-** SuPhenEx reaction.

4.1 Equipment and procedures

Mother liquor: Compound 5 （77.36 mg, 0.20 mmol）was dissolved in CH_3CN (4 ml); 4-trifluoromethylphenol (324.2 mg, 2.00 mmol) was dissolved in CH_3CN (8 ml).

In a 5-mL round-bottom flask equipped with an oil bath, the starting material **5** (1.0 equiv, 140 µL, 0.007 mmol) and phenol (10.0 equiv, 280 µL, 0.07 mmol) were dissolved in 0.58 ml of CH_3CN and heated to 41 ℃ for 30 min. In a 5-mL round-bottom flask, DBU was heated to 41 ℃ for 30 min at the same time. DBU (10.0 equiv,10.44 µL) was transferred to the starting materials vial using micropipette to start the reaction. Samples (each time 10 µl) were collected at particular time intervals. Each sample was quenched by the addition of isopropanol (0.60 mL). Obtained samples were analyzed by chiral HPLC (*n*-hexane/isopropanol = 95:5, flow rate 0.5 mL/min, detector wavelength 254 nm in CHIRALPAK®IA column). In the competition between SuFEx and SuPhenEx reactions (**Scheme S10**), the starting material **5** (28.5 min & 34.8 min) and the corresponding phenol (15.0 min) were used. In chiral HPLC, the peak area for peak at 41.6 min (compound **6**) was obtained to determine the conversion of compound **5** in SuFEx reaction (reaction **IIIa)** while the peak area at 11.6 min and 12.8 min (compound **3**) were integrated to determine the conversion of compound **5** in SuPhenEx reaction (reaction **IIIb)**. To determine the rate constant, pseudo–first order kinetics was applied; see section **3.3**.

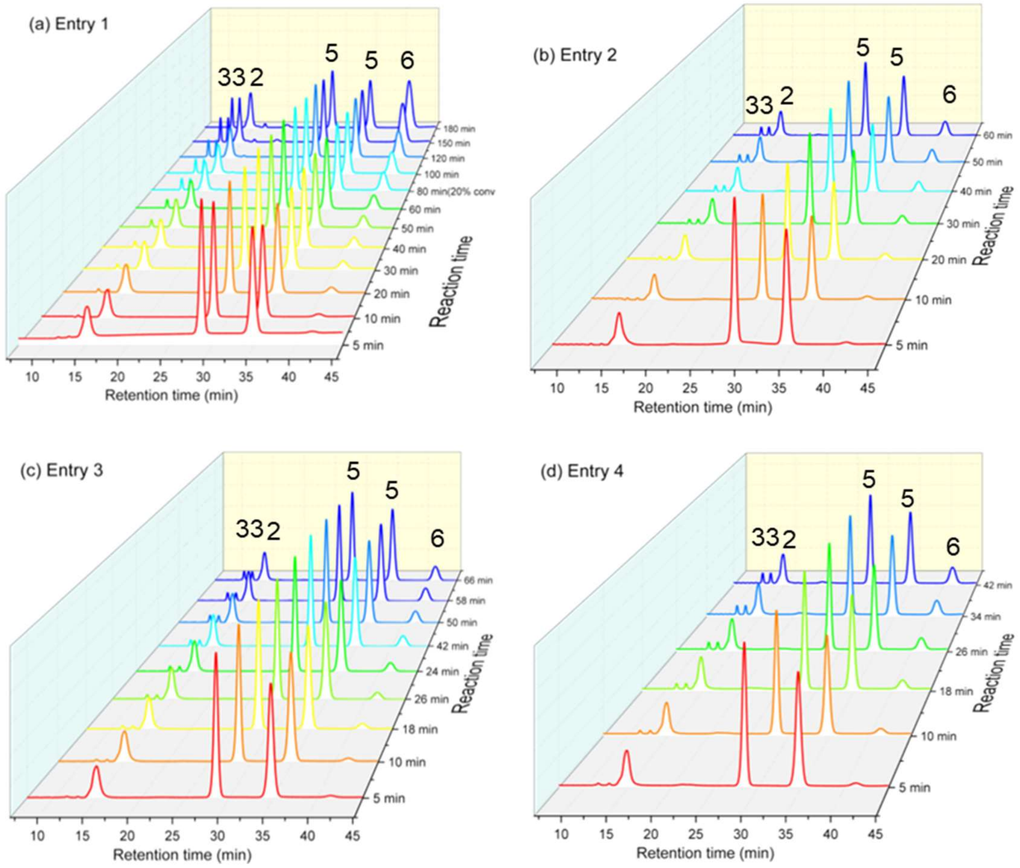

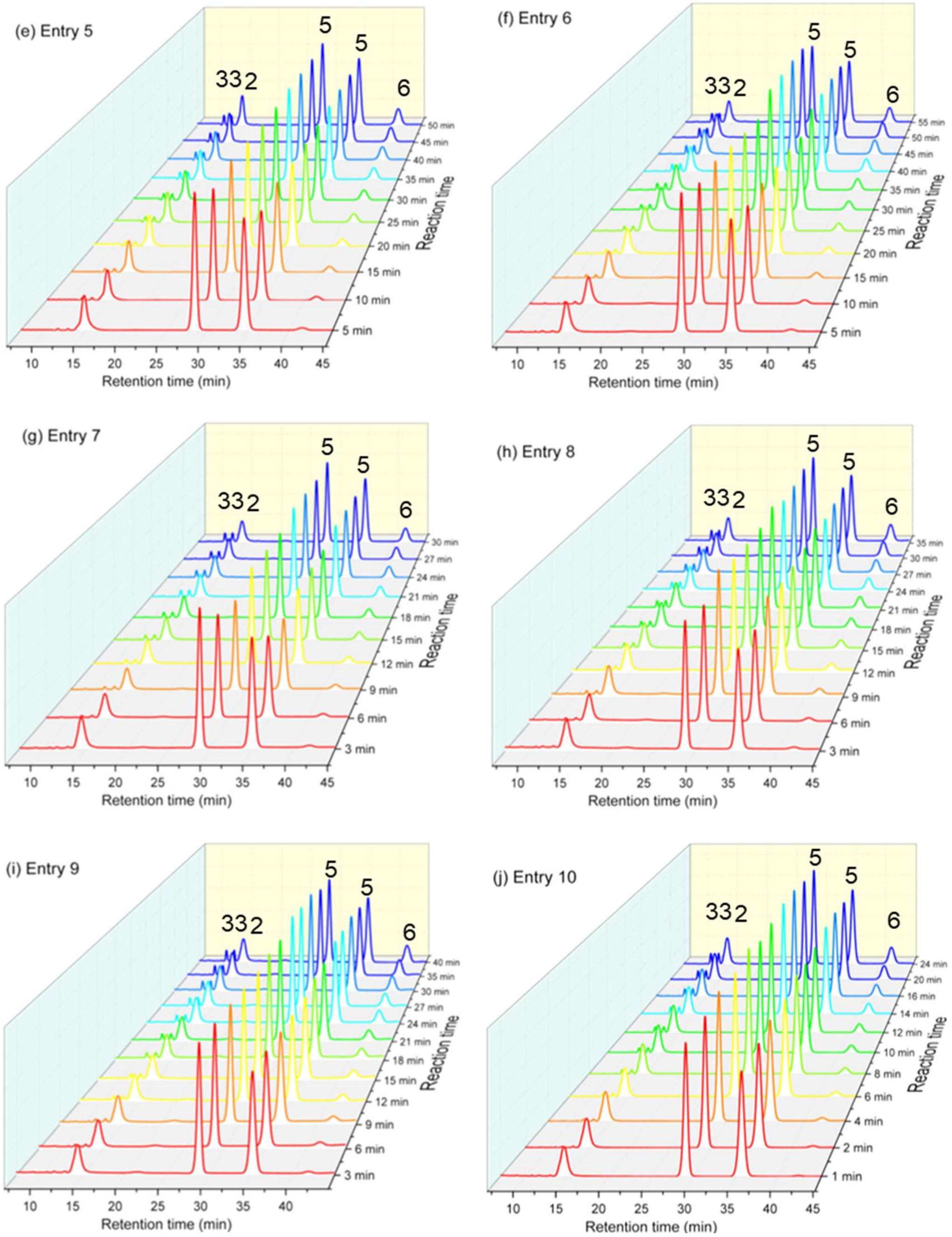
(e) Entry 5
(f) Entry 6
(g) Entry 7
(h) Entry 8
(i) Entry 9
(j) Entry 10
3
3
2
5
5
6
Retention time (min)
Reaction time

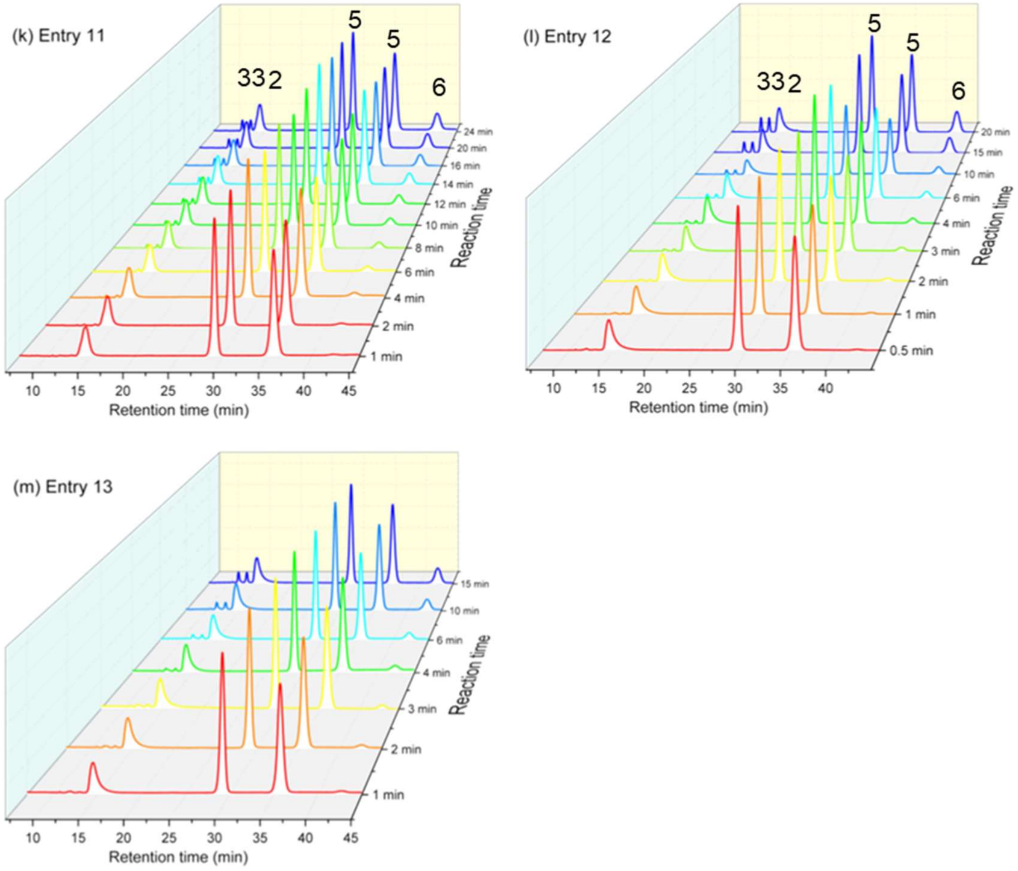

Figure S9. HPLC data used for the rate determination of the competitive SuFEx and SuPhenEx reactions of the second substituent, *i.e.*, in compound **5** with 4-trifluoromethylphenol at (a-c) 41 °C, (d-f) 48 °C, (g-i) 56 °C, (j-k) 63 °C, (l-m) 71 °C (see **Scheme S10**).

4.2 Rate determination of SuFEx and SuPhenEx reactions at different temperature

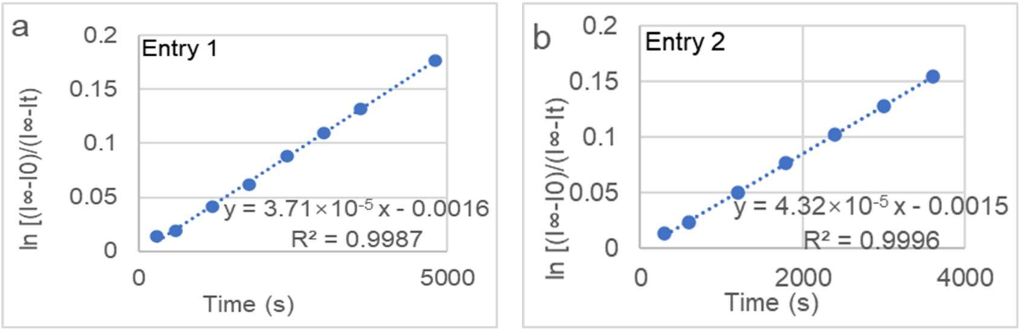

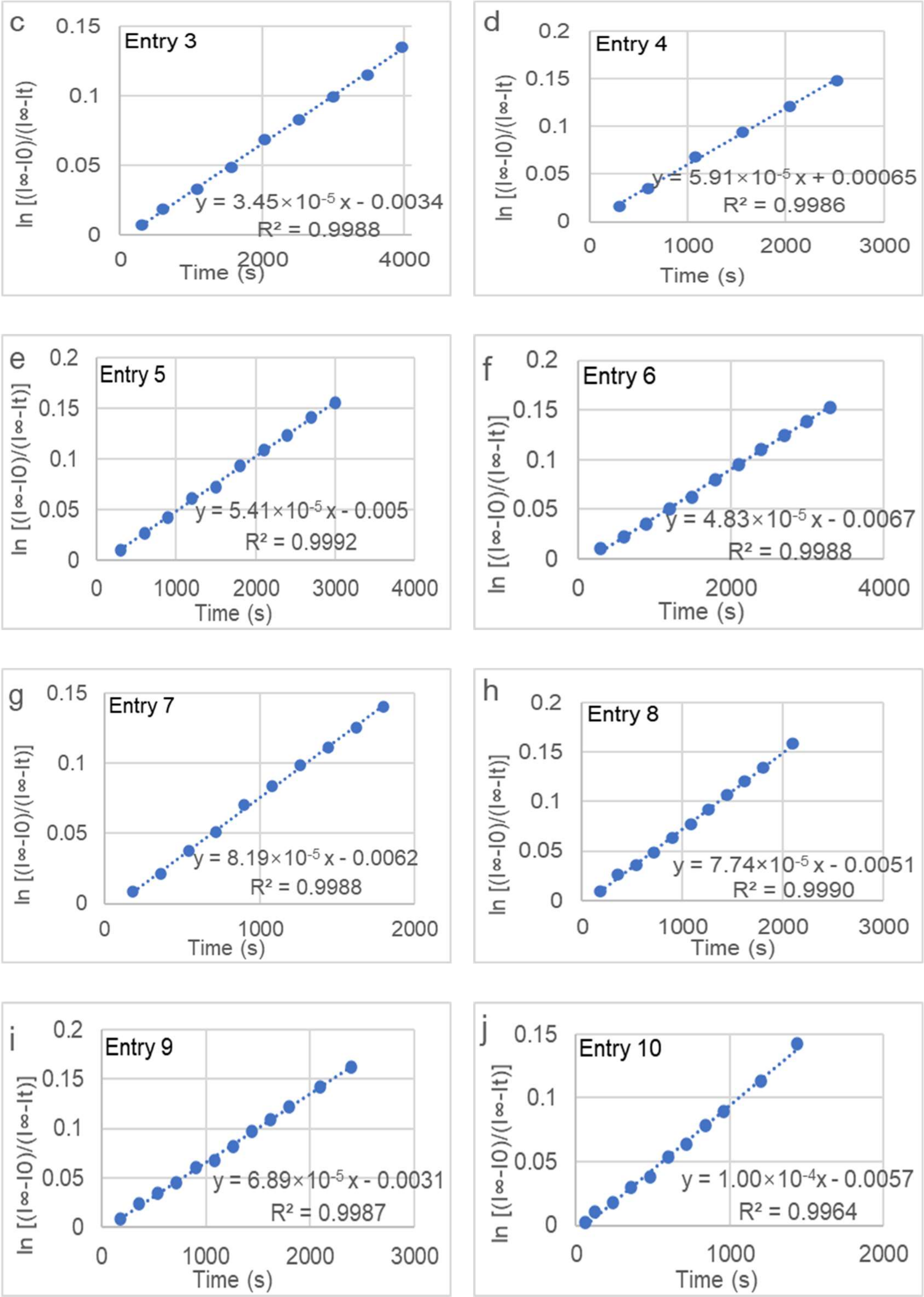
c
Entry 3
y = 3.45×10-5 x - 0.0034
R² = 0.9988
ln [(I∞-I0)/(I∞-It)
Time (s)
d
Entry 4
y = 5.91×10-5 x + 0.00065
R² = 0.9986
ln [(I∞-I0)/(I∞-It)]
Time (s)
e
Entry 5
y = 5.41×10-5 x - 0.005
R² = 0.9992
ln [(I∞-I0)/(I∞-It)]
Time (s)
f
Entry 6
y = 4.83×10-5 x - 0.0067
R² = 0.9988
ln [(I∞-I0)/(I∞-It)]
Time (s)
g
Entry 7
y = 8.19×10-5 x - 0.0062
R² = 0.9988
ln [(I∞-I0)/(I∞-It)]
Time (s)
h
Entry 8
y = 7.74×10-5 x - 0.0051
R² = 0.9990
ln [(I∞-I0)/(I∞-It)]
Time (s)
i
Entry 9
y = 6.89×10-5 x - 0.0031
R² = 0.9987
ln [(I∞-I0)/(I∞-It)]
Time (s)
j
Entry 10
y = 1.00×10-4x - 0.0057
R² = 0.9964
ln [(I∞-I0)/(I∞-It)]
Time (s)

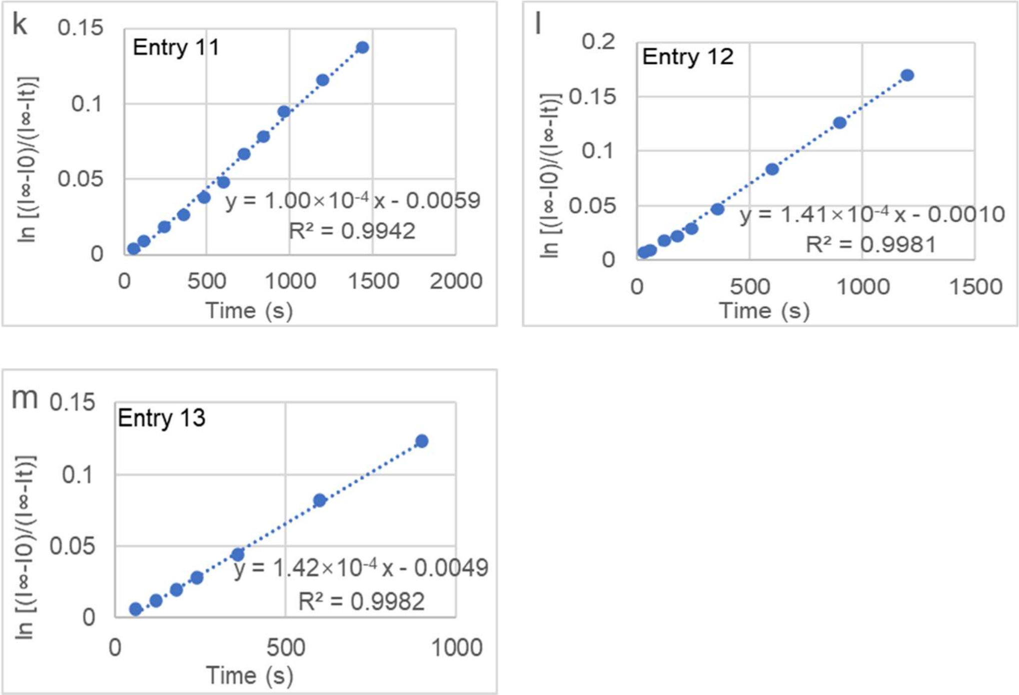

Figure S10. Calculation of the rates of the SuFEx reaction (reaction **IIIa**, **Scheme S10**) at different temperatures in CH_3CN. Conversions are obtained from HPLC at (a-c) 41 °C, (d-f) 48 °C, (g-i) 56 °C, (j-k) 63 °C, (l-m) 71 °C.

Table S6. Reaction rates for SuFEx reaction **IIIa** (**Scheme S10**) at different temperatures

Entry	Temperature	$k'(s^{-1})$	$k_2(M^{-1}s^{-1})$	Average rate with standard deviation/$(M^{-1}s^{-1})$
1	41 °C	3.71×10^{-5}	5.35×10^{-4}	
2	41 °C	4.32×10^{-5}	6.23×10^{-4}	$(5.52 \pm 0.52) \times 10^{-4}$
3	41 °C	3.45×10^{-5}	4.98×10^{-4}	
4	48 °C	5.91×10^{-5}	8.53×10^{-4}	
5	48 °C	5.41×10^{-5}	7.80×10^{-4}	$(7.77 \pm 0.64) \times 10^{-4}$
6	48 °C	4.83×10^{-5}	6.97×10^{-4}	
7	56 °C	8.19×10^{-5}	1.18×10^{-3}	
8	56 °C	7.74×10^{-5}	1.12×10^{-3}	$(1.10 \pm 0.08) \times 10^{-3}$
9	56 °C	6.89×10^{-5}	9.95×10^{-4}	
10	63 °C	1.00×10^{-4}	1.45×10^{-3}	
11	63 °C	1.00×10^{-4}	1.44×10^{-3}	$(1.44 \pm 0.01) \times 10^{-3}$
12	71 °C	1.41×10^{-4}	2.04×10^{-3}	
13	71 °C	1.42×10^{-4}	2.05×10^{-3}	$(2.04 \pm 0.01) \times 10^{-3}$

(When the conversion rate of compound **2** reaches to 20%, the initial concentration of phenol is 0.07 M/L, and the final concentration is 0.0686 M/L. For calculation of the second-order rate constant, the average concentration, 0.0693 M/L was used.)

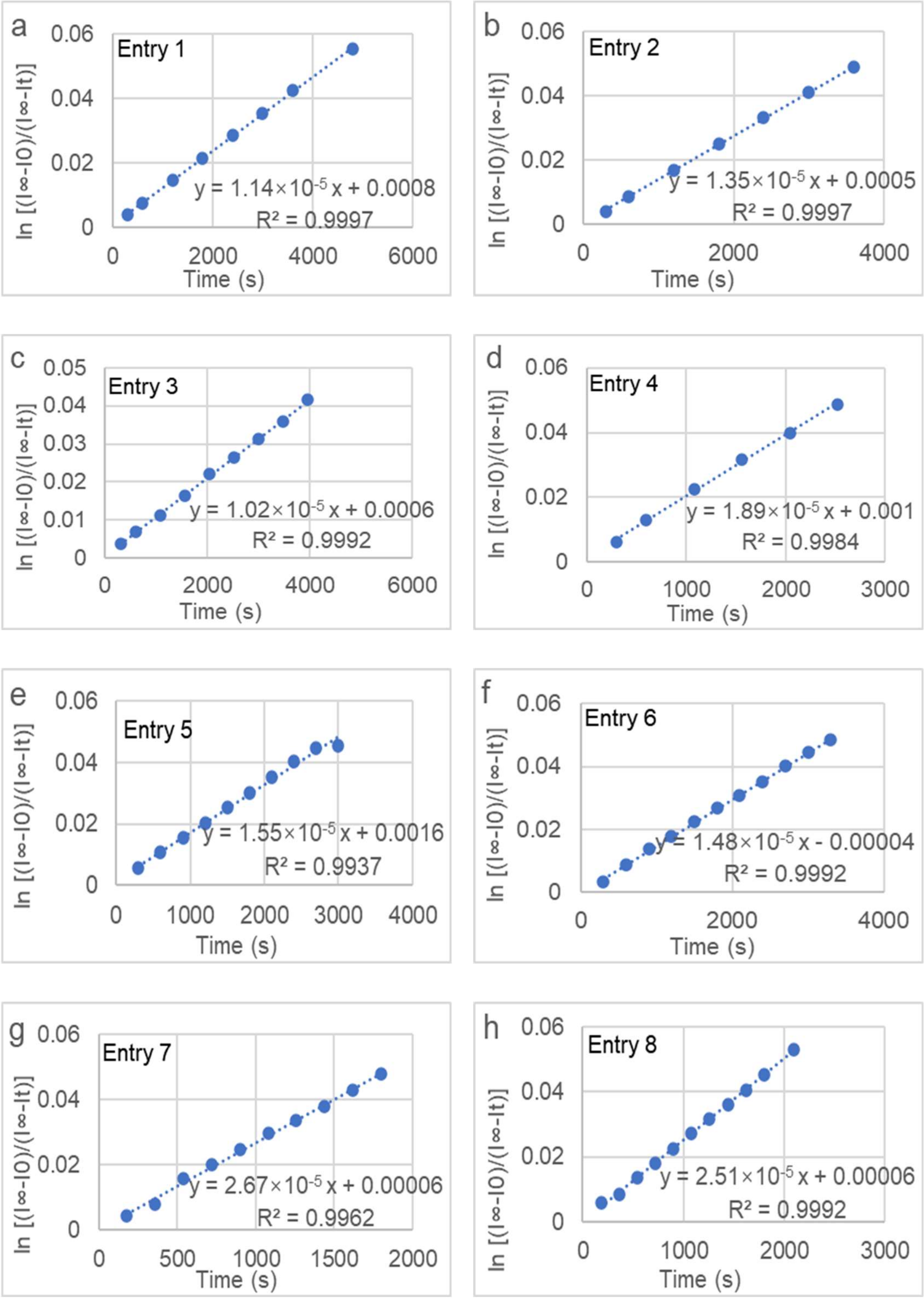
a
Entry 1
y = 1.14×10-5 x + 0.0008
R² = 0.9997
b
Entry 2
y = 1.35×10-5 x + 0.0005
R² = 0.9997
c
Entry 3
y = 1.02×10-5 x + 0.0006
R² = 0.9992
d
Entry 4
y = 1.89×10-5 x + 0.001
R² = 0.9984
e
Entry 5
y = 1.55×10-5 x + 0.0016
R² = 0.9937
f
Entry 6
y = 1.48×10-5 x - 0.00004
R² = 0.9992
g
Entry 7
y = 2.67×10-5 x + 0.00006
R² = 0.9962
h
Entry 8
y = 2.51×10-5 x + 0.00006
R² = 0.9992
ln [(I∞-I0)/(I∞-It)]
Time (s)

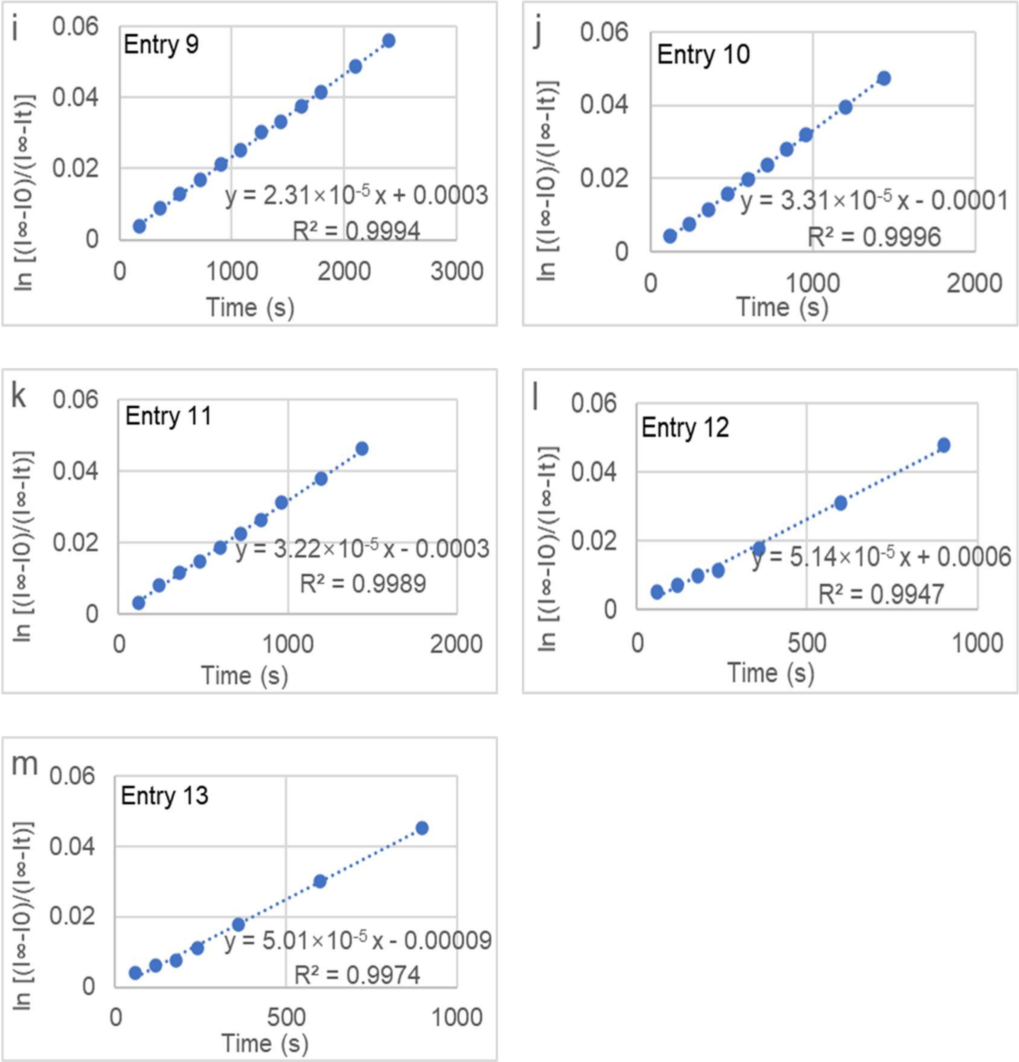

Figure S11. Calculation of the reaction rates of the SuPhenEx reaction (reaction **IIIb**, **Scheme S10**) at different temperatures in CH_3CN. Conversions are obtained from HPLC at (a-c) 41 °C, (d-f) 48 °C, (g-i) 56 °C, (j-k) 63 °C, (l-m) 71 °C.

Table S7. Reaction rates for SuPhenEx **IIIb** reaction rates (**Scheme S10**) at different temperatures

Entry	Temperature	k'(s^{-1})	k_2($M^{-1}s^{-1}$)	Average rate with standard deviation/($M^{-1}s^{-1}$)
1	41 °C	1.14 ×10^{-5}	1.65 ×10^{-4}	(1.69±0.19) ×10^{-4}
2	41 °C	1.35 ×10^{-5}	1.95 ×10^{-4}	
3	41 °C	1.02 ×10^{-5}	1.48 ×10^{-4}	
4	48 °C	1.89 ×10^{-5}	2.73 ×10^{-4}	(2.37±0.26) ×10^{-4}
5	48 °C	1.55 ×10^{-5}	2.24 ×10^{-4}	
6	48 °C	1.48 ×10^{-5}	2.14 ×10^{-4}	
7	56 °C	2.67 ×10^{-5}	3.85 ×10^{-4}	(3.60±0.21) ×10^{-4}
8	56 °C	2.51 ×10^{-5}	3.62 ×10^{-4}	
9	56 °C	2.31 ×10^{-5}	3.34 ×10^{-4}	
10	63 °C	3.31 ×10^{-5}	4.78 ×10^{-4}	(4.71±0.05) ×10^{-4}
11	63 °C	3.22 ×10^{-5}	4.65 ×10^{-4}	
12	71 °C	5.14 ×10^{-5}	7.42 ×10^{-4}	(7.32±0.09) ×10^{-4}
13	71 °C	5.01 ×10^{-5}	7.23 ×10^{-4}	

(When the conversion rate of compound **2** reaches to 20%, the initial concentration of phenol is 0.07 M/L, and the final concentration is 0.0686 M/L. When the second-order rate constant is calculated, the average concentration, 0.0693 M/L was used)

4.3 Determination of activation enthalpy from the reaction rates (reactions IIIa & IIIb, Scheme S10) using the Eyring equation.

Table S8. Parameters of reaction **IIIa** (**Scheme S10**) for Eyring equation.

Temperature K	1/Temperature (1/K)	k	k/T	ln k/T
314.15	3.18 ×10^{-3}	5.52 ×10^{-4}	1.76 ×10^{-6}	-13.25
321.15	3.11 ×10^{-3}	7.78 ×10^{-4}	2.42 ×10^{-6}	-12.93
329.15	3.04 ×10^{-3}	1.10 ×10^{-3}	3.34 ×10^{-6}	-12.61
336.15	2.97 ×10^{-3}	1.44 ×10^{-3}	4.30 ×10^{-6}	-12.36
344.15	2.91 ×10^{-3}	2.04 ×10^{-3}	5.94 ×10^{-6}	-12.03

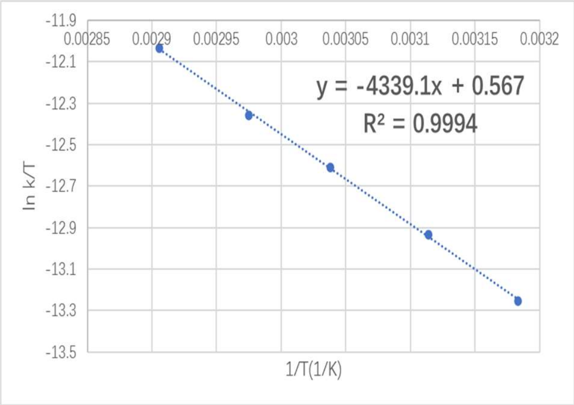

Figure S12. Eyring plot for the SuFEx reaction (reaction **IIIa**, **Scheme S10**) to determine the activation enthalpy.

Slope = $(-\Delta H^{\ddagger})/R$

$-4339.1 = -\Delta H^{\ddagger}/8.314$ J M^{-1} K^{-1}

$\Delta H^{\ddagger}$ = 4339.1 K × 8.314 J M^{-1} K^{-1} = 36.07 kJ M^{-1} = 8.62 kcal M^{-1}

Therefore, an activation enthalpy was determined to be 7.44 kcal M^{-1} for the SuFEx reaction. Further, the Eyring equation was used to determine the activation entropy for this SuFEx reaction. k_B is the Boltzmann's constant (1.381 × 10^{-23} J/K); h is Planck's constant (6.626 × 10^{-34} Js).

$\ln(k_B/h) = 23.76$

$0.564 = 23.76 + \Delta S^{\ddagger}/R$

$\Delta S^{\ddagger}$/ 8.314 J M^{-1} K^{-1}= -23.19

$\Delta S^{\ddagger}$ = -23.19 × 8.314 J M^{-1} K^{-1} = -0.193 kJ M^{-1} K^{-1} = -0.0461 kcal M^{-1} K^{-1}

$\Delta G^{\ddagger} = \Delta H^{\ddagger} - T\Delta S^{\ddagger}$

Here $\Delta G_{25}^{\ddagger}$ ($\Delta G^{\ddagger}$ at 25 °C) could be calculated as

$\Delta G_{25}^{\ddagger}$ = 8.62 kcal M^{-1} – 298.15 K × -0.0461 kcal M^{-1} K^{-1} = 22.36 kcal M^{-1}

Therefore $\Delta G_{25}^{\ddagger}$ was found to be 22.36 kcal M^{-1} and $\Delta S^{\ddagger}$ was found to be -0.0441 kcal M^{-1} K^{-1}

Table S9. Reaction rates of reaction **IIIb** (**Scheme S10**) for use in the Eyring equation.

Temperature K	1/Temperature (1/K)	*k*	*k*/T	ln *k*/T
314.15	3.18 ×10^{-3}	1.69 ×10^{-4}	5.38 ×10^{-7}	-14.43
321.15	3.11 ×10^{-3}	2.37 ×10^{-4}	7.38 ×10^{-7}	-14.12
329.15	3.04 ×10^{-3}	3.60 ×10^{-4}	1.09 ×10^{-6}	-13.73
336.15	2.97 ×10^{-3}	4.71 ×10^{-4}	1.40 ×10^{-6}	-13.48

344.15	2.91×10^{-3}	7.32×10^{-4}	2.13×10^{-6}	-13.06

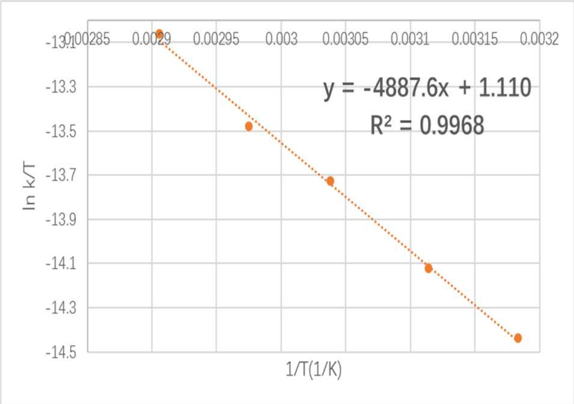

Figure S13: Eyring plot for the SuPhenEx reaction (reaction **IIIb**, **Scheme S10**) to determine the activation enthalpy.

Slope = $(-\Delta H^{\ddagger})/R$
$-4887.6 = -\Delta H^{\ddagger}/8.314$ J M^{-1} K^{-1}
$\Delta H^{\ddagger} = 4887.6$ K × 8.314 J M^{-1} K^{-1} = 40.64 kJ M^{-1} = 9.71 kcal M^{-1}

Therefore, an activation enthalpy was calculated to be 9.71 kcal M^{-1} for the SuPhenEx reaction. Further, the Eyring equation was used to determine activation entropy change for the SuPhenEx reaction. k_B is the Boltzmann's constant (1.381×10^{-23} J/K); h is Planck's constant (6.626×10^{-34} Js).

$\ln(k_B/h) = 23.76$
$1.10 = 23.76 + \Delta S^{\ddagger}/R$
$\Delta S^{\ddagger}/ 8.314$ J M^{-1} K^{-1}= -22.65
$\Delta S^{\ddagger} = -22.65 \times 8.314$ J M^{-1} K^{-1} = -0.188 kJ M^{-1} K^{-1} = -0.0449 kcal M^{-1} K^{-1}
$\Delta G^{\ddagger} = \Delta H^{\ddagger} - T\Delta S^{\ddagger}$

Here $\Delta G_{25}^{\ddagger}$ ($\Delta G^{\ddagger}$ at 25 ^{o}C) could be calculated as

$\Delta G_{25}^{\ddagger}$ = 9.71 kcal M^{-1} – 298.15 K × -0.0449 kcal M^{-1} K^{-1} = 23.10 kcal M^{-1}

Therefore $\Delta G_{25}^{\ddagger}$ was found to be 23.10 kcal M^{-1} and $\Delta S^{\ddagger}$ was found to be -0.0449 kcal M^{-1} K^{-1}.

5. Reaction kinetics of first group in S(VI) for SuPhenEx reactions at different temperatures

The reaction kinetics of the SuPhenEx reaction in **Scheme S9** was studied using the starting material **7** (1.0 equiv), which was reacted with sodium 4-bromophenolate. Reactions were monitored using chiral HPLC. Kinetic measurements were performed at 50, 60, 65, 70, 75 and 80 °C.

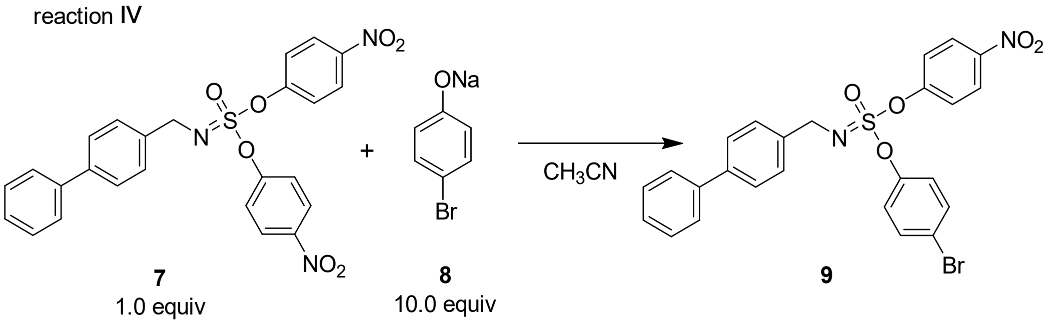

Scheme S11. Model SuPhenEx reaction employed for kinetic studies, reaction **IV**- first group in S(VI) for SuPhenEx reaction.

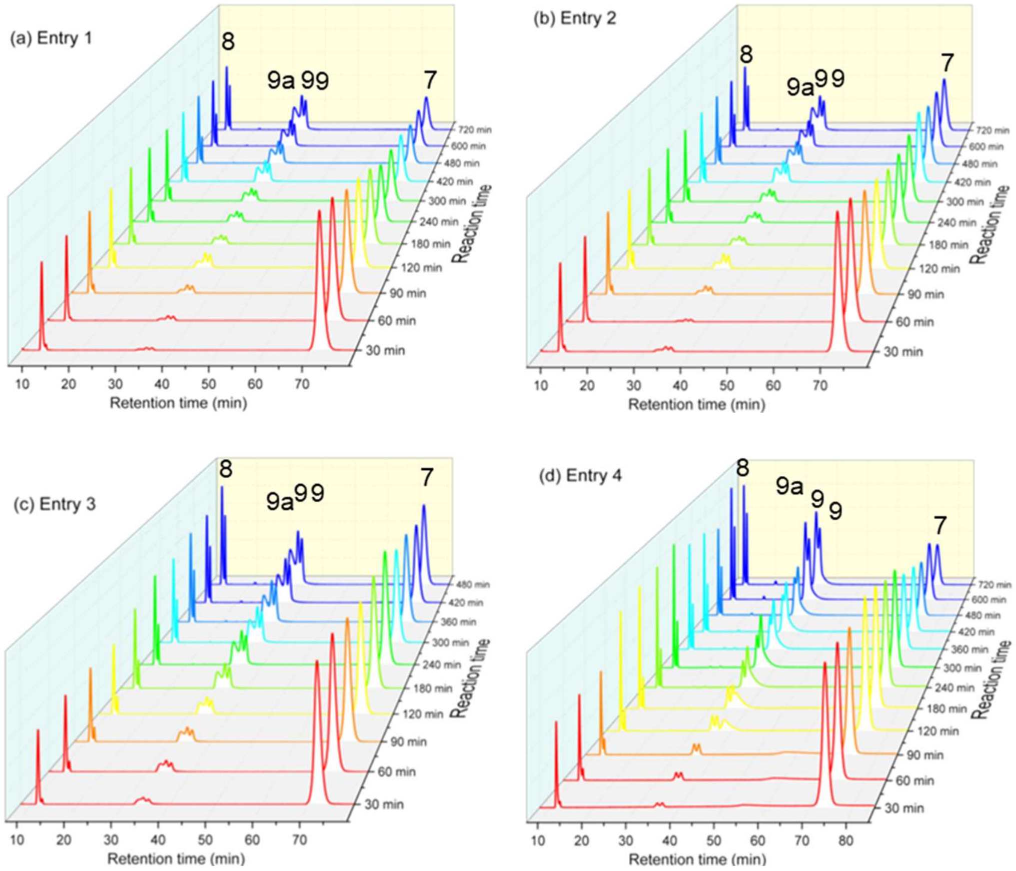

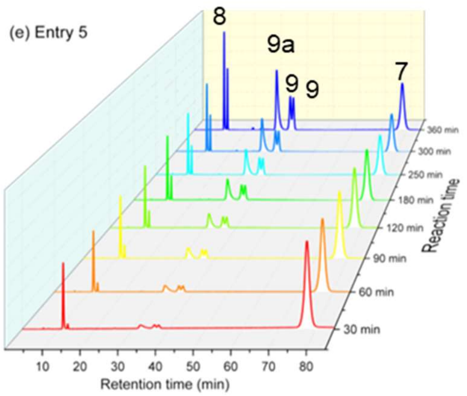
(e) Entry 5
8
9a
9 9
7
360 min
300 min
250 min
180 min
120 min
90 min
60 min
30 min
Reaction time
10 20 30 40 50 60 70 80
Retention time (min)

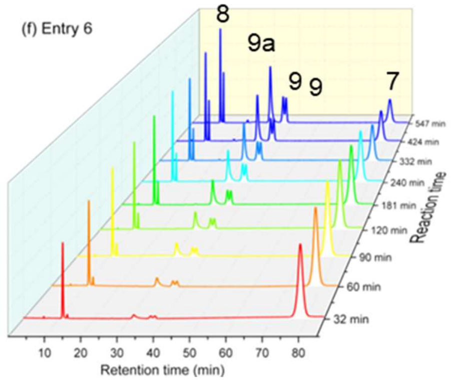
(f) Entry 6
8
9a
9 9
7
547 min
424 min
332 min
240 min
181 min
120 min
90 min
60 min
32 min
Reaction time
10 20 30 40 50 60 70 80
Retention time (min)

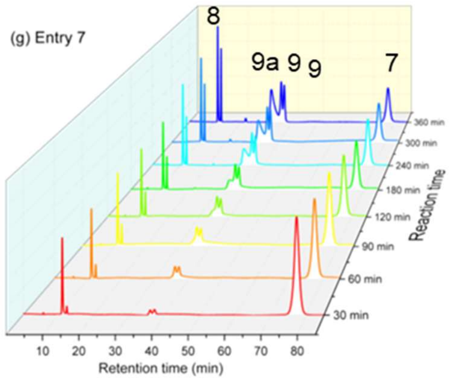
(g) Entry 7
8
9a 9 9
7
360 min
300 min
240 min
180 min
120 min
90 min
60 min
30 min
Reaction time
10 20 30 40 50 60 70 80
Retention time (min)

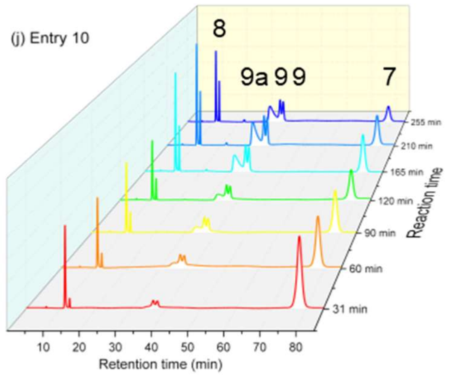
(j) Entry 10
8
9a 9 9
7
255 min
210 min
165 min
120 min
90 min
60 min
31 min
Reaction time
10 20 30 40 50 60 70 80
Retention time (min)

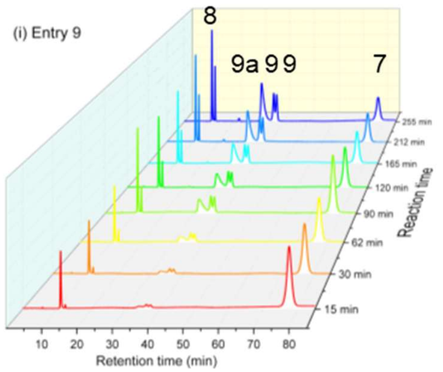
(i) Entry 9
8
9a 9 9
7
255 min
212 min
165 min
120 min
90 min
62 min
30 min
15 min
Reaction time
10 20 30 40 50 60 70 80
Retention time (min)

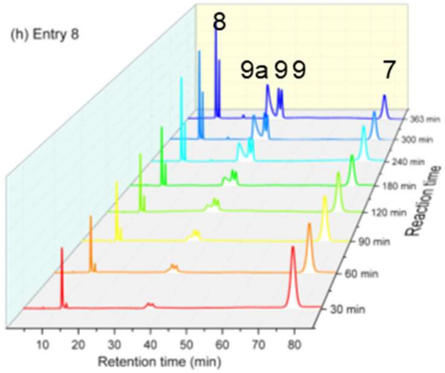
(h) Entry 8
8
9a 9 9
7
363 min
300 min
240 min
180 min
120 min
90 min
60 min
30 min
Reaction time
10 20 30 40 50 60 70 80
Retention time (min)

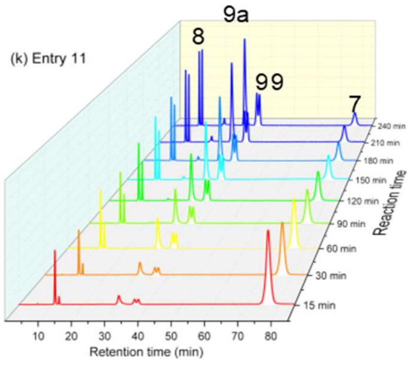
(k) Entry 11
9a
8
9 9
7
240 min
210 min
180 min
150 min
120 min
90 min
60 min
30 min
15 min
Reaction time
10 20 30 40 50 60 70 80
Retention time (min)

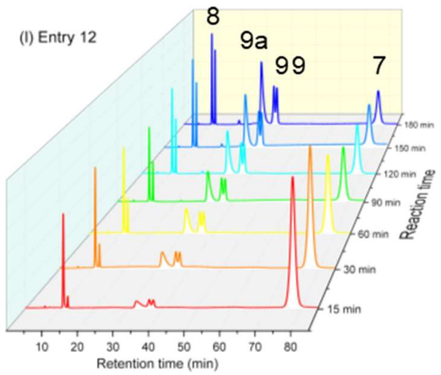
(l) Entry 12
8
9a
9 9
7
180 min
150 min
120 min
90 min
60 min
30 min
15 min
Reaction time
10 20 30 40 50 60 70 80
Retention time (min)

Figure S14. HPLC data used for the rate determination of the SuPhenEx reactions of first *p*-NO_2 in compound **7** with 4-bromophenolate at (a-b) 50 °C, (c-d) 60 °C, (e-f) 65 °C, (g-h) 70 °C, (i-j) 75 °C, (k-l) 80 °C (reaction **IV**, **Scheme S11**).

5.1 Equipment and procedures

Mother liquor: Compound **7** (239 mg, 0.47 mmol) was dissolved in CH_3CN (5 ml). In a 5-mL round-bottom flask equipped with an oil bath, 4-bromophenolate (78 mg, 0.4 mmol) was dissolved in 578 μL CH_3CN, then the solution was to heated to certain degree (50 °C to 80 °C) in 30 min, and the mother liquor of **7** in a vial was also heated to the same degree in 30 min. Adding compound **7** (422 μL, 0.04 mmol) into the flask to start the reaction. Samples (each time 10 μl) were collected at particular time intervals. Each sample was quenched by the addition of isopropanol (1 mL). Obtained samples were analyzed by chiral HPLC (*n*-hexane/isopropanol = 90:10, flow rate 0.5 mL/min, detector wavelength 254 nm in CHIRALPAK®IA column). In chiral HPLC, the starting material **7** (77.6 min) and the corresponding phenolate (11.2 min) were used. In chiral HPLC, the peak areas for the peaks at 35.9 min and 37.1 min (compound **9**) and 31.4 min (compound **9a-**hydrolysate of compound **9**) were integrated to determine the conversion of the first group in S(VI) in SuPhenEx reaction. To determine the rate constant, the method used for pseudo– first order kinetics was shown in section **3.3**.

Table S10. Reaction rates of reaction **IV**, **Scheme S11**, at different temperatures.

Entry	Temperature	*k*' (s^{-1})	k_2 ($M^{-1}s^{-1}$)	Average rate with standard deviation ($M^{-1}s^{-1}$)
1	50 °C	1.56×10^{-5}	3.99×10^{-5}	$(4.16 \pm 0.16) \times 10^{-5}$
2	50 °C	1.68×10^{-5}	4.32×10^{-5}	
3	60 °C	2.55×10^{-5}	6.53×10^{-5}	$(6.79 \pm 0.26) \times 10^{-5}$
4	60 °C	2.75×10^{-5}	7.05×10^{-5}	
5	65 °C	4.23×10^{-5}	1.08×10^{-4}	$(1.02 \pm 0.06) \times 10^{-4}$
6	65 °C	3.71×10^{-5}	9.51×10^{-5}	
7	70 °C	5.57×10^{-5}	1.43×10^{-4}	$(1.44 \pm 0.02) \times 10^{-4}$
8	70 °C	5.69×10^{-5}	1.46×10^{-4}	
9	75 °C	8.52×10^{-5}	2.18×10^{-4}	$(2.26 \pm 0.07) \times 10^{-4}$
10	75 °C	9.07×10^{-5}	2.33×10^{-4}	
11	80 °C	1.16×10^{-4}	2.98×10^{-4}	$(3.00 \pm 0.02) \times 10^{-4}$
12	80 °C	1.18×10^{-4}	3.02×10^{-4}	

(When the conversion rate of compound **3** reaches to 50%, the initial concentration of phenolate is 0.40 M/L, and the final concentration is 0.38 M/L. When the second-order rate constant is calculated, the average concentration, 0.39 M/L was used)

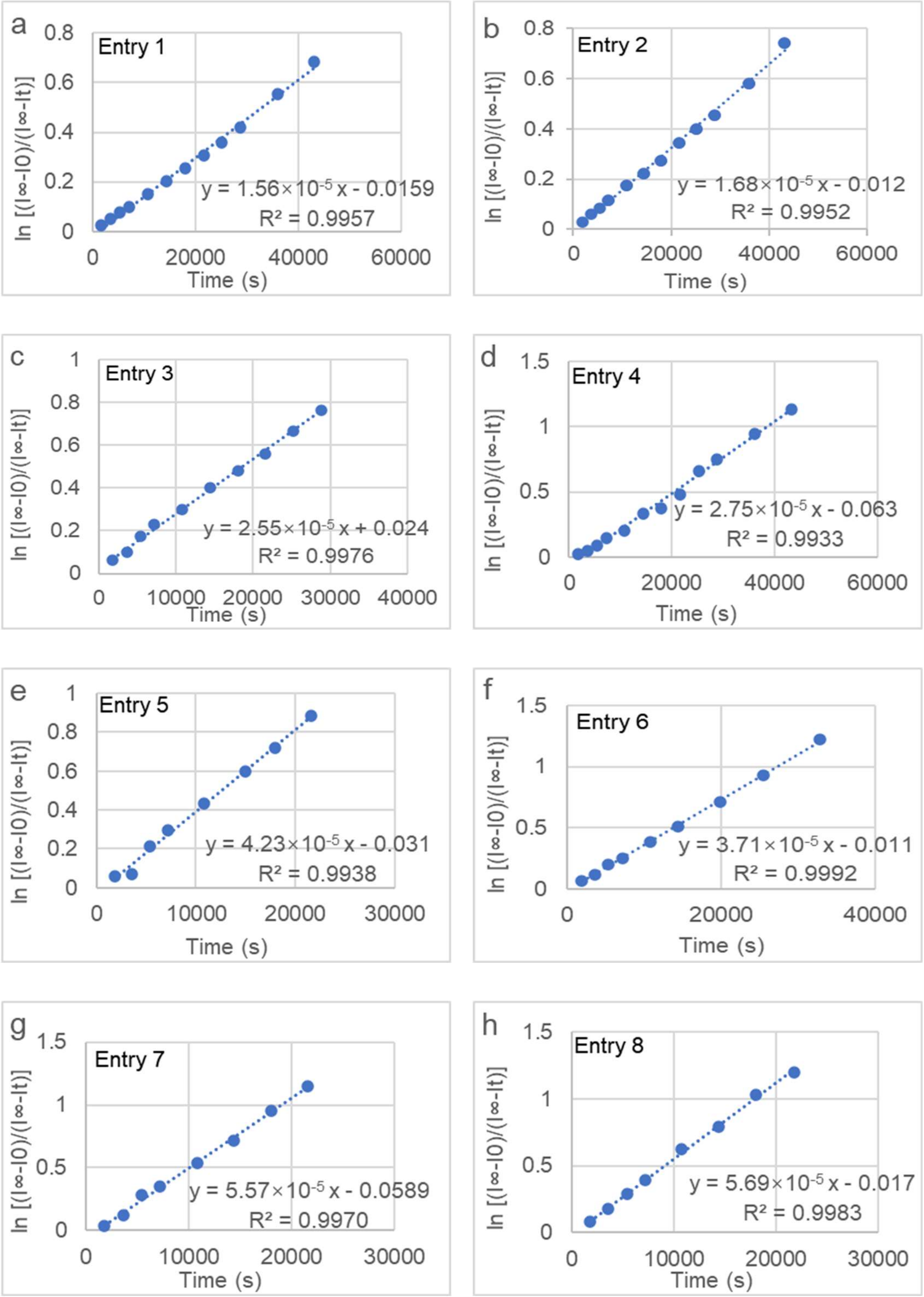
a
Entry 1
y = 1.56×10-5 x - 0.0159
R² = 0.9957
b
Entry 2
y = 1.68×10-5 x - 0.012
R² = 0.9952
c
Entry 3
y = 2.55×10-5 x + 0.024
R² = 0.9976
d
Entry 4
y = 2.75×10-5 x - 0.063
R² = 0.9933
e
Entry 5
y = 4.23×10-5 x - 0.031
R² = 0.9938
f
Entry 6
y = 3.71×10-5 x - 0.011
R² = 0.9992
g
Entry 7
y = 5.57×10-5 x - 0.0589
R² = 0.9970
h
Entry 8
y = 5.69×10-5 x - 0.017
R² = 0.9983
ln [(I∞-I0)/(I∞-It)]
Time (s)

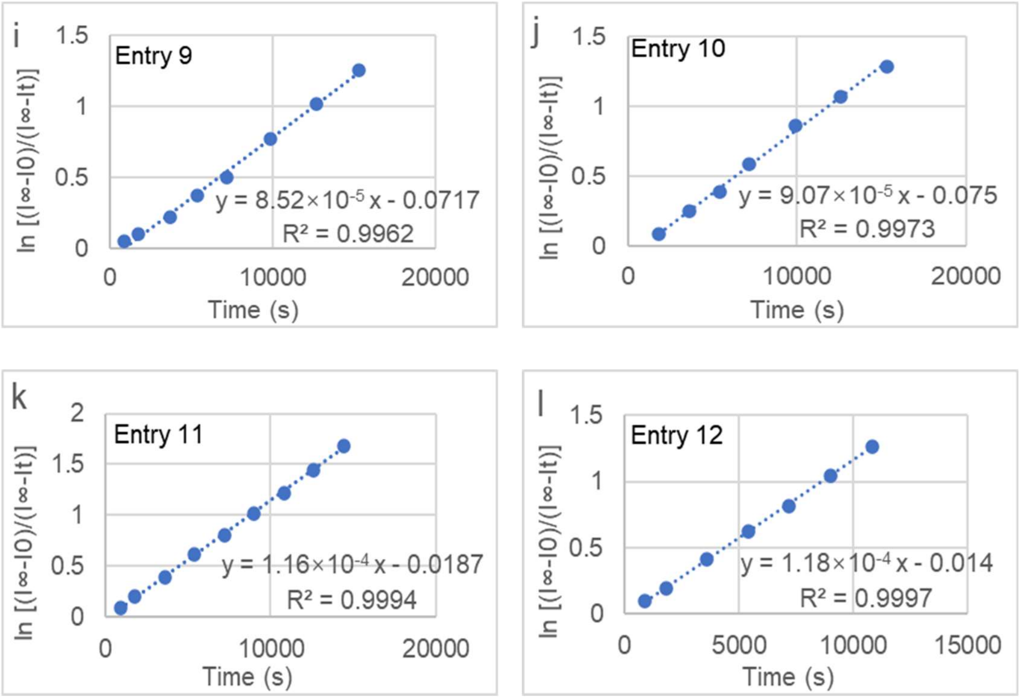

Figure S15. Calculation of the rates of the SuPhenEx reaction (reaction **IV**, **Scheme S11**) at different temperatures in CH_3CN. Conversions are obtained from HPLC at (a-b) 50 °C, (c-d) 60 °C, (e-f) 65 °C, (g-h) 70 °C, (i-j) 75 °C, (k-l) 80 °C.

5.2 Determination of activation enthalpy from the reaction rates (reaction IV, Scheme S11) using Eyring equation.

Table S11. Reaction rates of reaction **IV** (**Scheme S11**) for use in the Eyring equation.

Temperature K	1/Temperature (1/K)	*k*	*k*/T	ln *k*/T
333.15	3.00×10^{-3}	6.79×10^{-5}	2.04×10^{-7}	-15.40
338.15	2.96×10^{-3}	1.02×10^{-4}	3.01×10^{-7}	-15.02
343.15	2.91×10^{-3}	1.44×10^{-4}	4.21×10^{-7}	-14.68
348.15	2.87×10^{-3}	2.26×10^{-4}	6.48×10^{-7}	-14.25
353.15	2.83×10^{-3}	3.00×10^{-4}	8.50×10^{-7}	-13.98
323.15	3.09×10^{-3}	4.16×10^{-5}	1.29×10^{-7}	-15.86

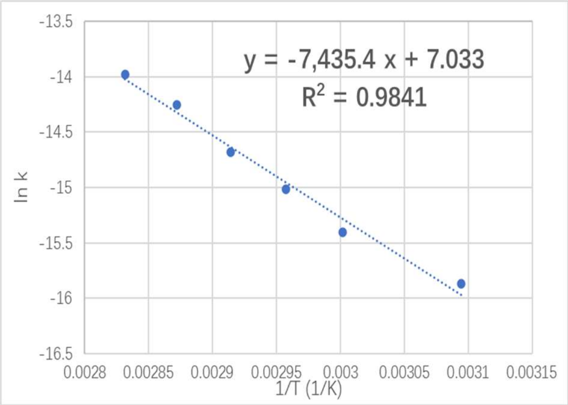

Figure S16. Eyring plot for the first group in S(VI) in SuPhenEx reaction (**Scheme S11**) to determine the activation enthalpy.

Slope = $(-\Delta H^{\ddagger})/R$
$-7435.4 = -\Delta H^{\ddagger}/8.314$ J M^{-1} K^{-1}
$\Delta H^{\ddagger} = 7435.4$ K × 8.314 J M^{-1} K^{-1} = 61.82 kJ M^{-1} = 14.77 kcal M^{-1}

Therefore, an activation enthalpy was calculated to be 14.77 Kcal M^{-1} for the first group in S(VI) in SuPhenEx reaction. Further, the Eyring equation was used to determine activation entropy change for first F SuFEx reaction. k_B is the Boltzmann's constant (1.381 × 10^{-23} J/K); h is Planck's constant (6.626 ×10^{-34} Js).

$\ln(k_B/h) = 23.76$
$7.033 = 23.76 + \Delta S^{\ddagger}/R$
$\Delta S^{\ddagger}$/ 8.314 J M^{-1} K^{-1}= -16.726
$\Delta S^{\ddagger}$ = -16.726 × 8.314 J M^{-1} K^{-1} = -0.139 kJ M^{-1} K^{-1} = -0.0334 kcal M^{-1} K^{-1}
$\Delta G^{\ddagger} = \Delta H^{\ddagger} - T\Delta S^{\ddagger}$

Here $\Delta G_{25}^{\ddagger}$ ($\Delta G^{\ddagger}$ at 25 ^{o}C) could be calculated as

$\Delta G_{25}^{\ddagger}$ = 14.77 kcal M^{-1} – 298.15 K × -0.0334 kcal M^{-1} K^{-1} = 24.73 kcal M^{-1}

Therefore $\Delta G_{25}^{\ddagger}$ was found to be 24.73 kcal M^{-1} and $\Delta S^{\ddagger}$ was found to be -0.0334 kcal M^{-1} K^{-1}.

6. Inversion of absolute configuration for SuPhenEx reaction

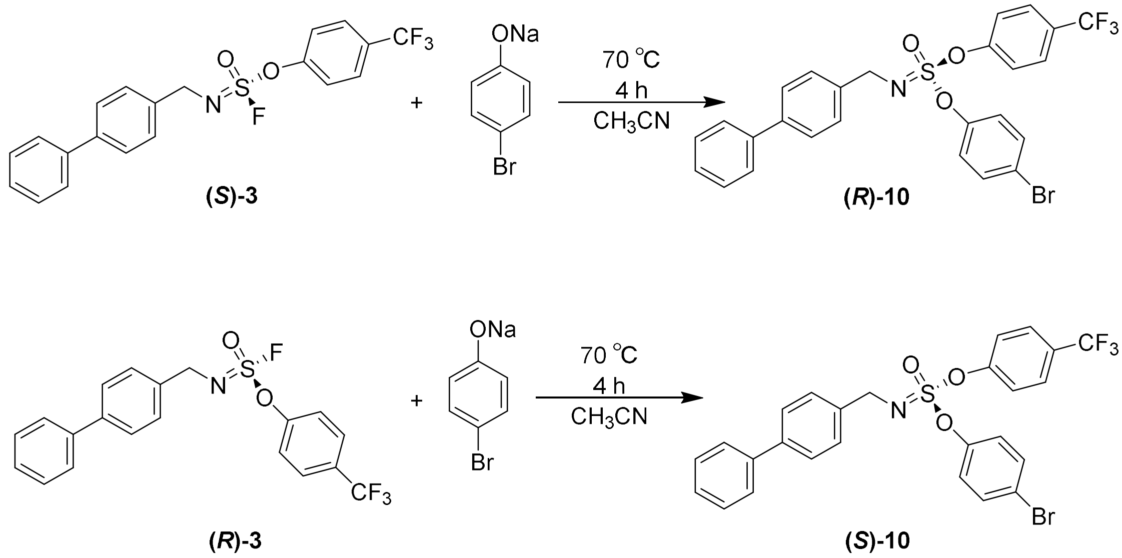

Scheme S13. Inversion of absolute configuration for SuFEx reaction of enantiomer **(*S*)-3** and **(*R*)-3**.

Compounds 10: (*S*)-3/(*R*)-3 (40 mg, 0.097 mmol, 98% *ee*, 1.0 equiv.) was dissolved in anhydrous CH_3CN (1 mL) under argon. Then 4-bromophenolate (38 mg, 0.19 mmol, 2.0 equiv.) was added to the solution. The reaction mixture was stirred for 3 h at 50 °C. The reaction was quenched by adding water (1 mL), extracted with CH_2Cl_2 (3 × 1 mL), dried with anhydrous $MgSO_4$ and purified by flash column chromatography ((EtOAc/*n*-hexane = 1:9) over silica gel (300−400 mesh) to afford **(*R*)-10** as solid (conv 79%, 41 mg, 0.07 mmol, 75%., >99% es) and **(*S*)-10** as solid (conv 74%, 39 mg, 0.07 mmol, 72%., >99% es). R_f = 0.57 (EtOAc/n-hexane = 1:4). Single crystals suitable for X-ray analysis were obtained by slow evaporation of a solution of the **compound (*R*)-10** in dry THF at room temperature overnight. **^{1}H NMR** (400 MHz, $CDCl_3$) δ 7.65 (d, *J* = 8.6 Hz, 2H), 7.59 (d, *J* = 8.1 Hz, 2H), 7.51 (t *J* = 8.9 Hz, 3H), 7.45 (t, *J* = 7.6 Hz, 2H), 7.34 (dd, *J* = 17.4, 8.6 Hz, 5H), 7.15 (d, *J* = 8.9 Hz, 2H), 4.54 (s, 2H). **^{19}F NMR** (376 MHz, $CDCl_3$) δ -62.35. **^{13}C {^{1}H} NMR** (101 MHz, $CDCl_3$) δ 152.8, 149.4, 140.9, 140.4, 138.0, 133.1, 128.9, 127.9, 127.4, 127.4, 127.4, 127.3, 127.3, 127.2, 123.6, 122.2, 120.8, 49.4. **HRMS (ESI)** m/z: $[M + H]^+$ Calcd for $C_{26}H_{20}BrO_3NF_3S^+$: 562.2394, Found: 562.0272. Melting point: 43.0-43.4 °C.

Single crystal of **compound (*S*)-3**, suitable for X-ray analysis, was obtained by dissolving (*S*)-3 in a volatile good solvent (acetonitrile), followed by careful layering with a less polar solvent (n-heptane) to maintain a biphasic system, allowing slow, spontaneous interfacial diffusion to proceed under ambient conditions.

7. NMR Spectra

All NMR spectra were taken at 298 K.

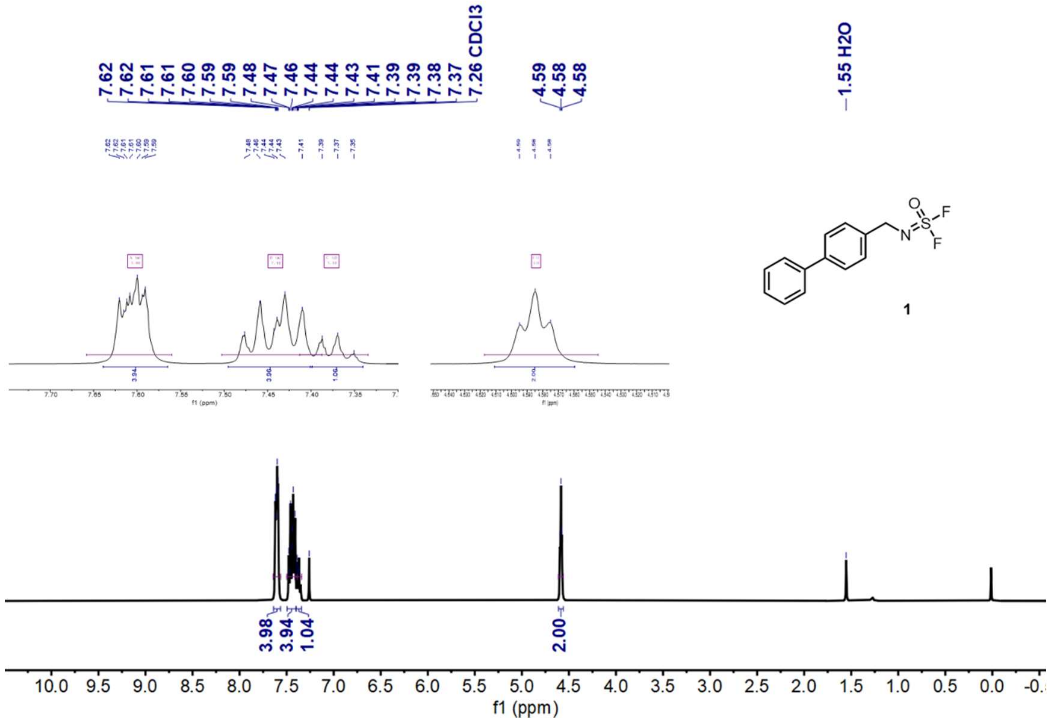

Figure S17. ^{1}H NMR (400 MHz) spectrum of compound **1** ($CDCl_3$).

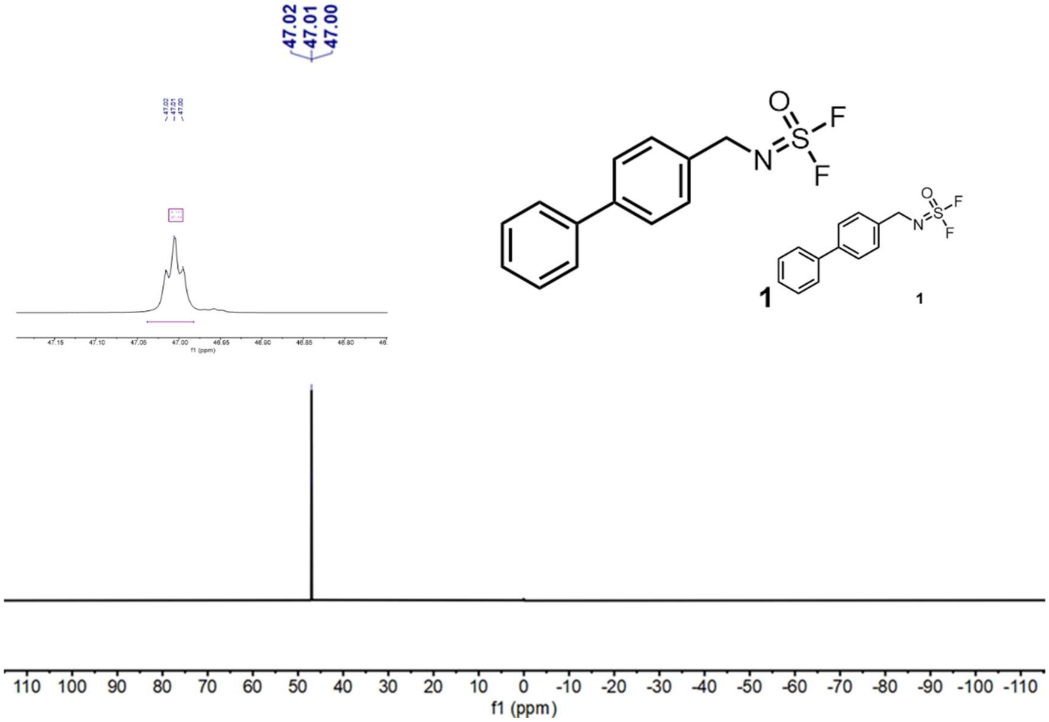

Figure S18. ^{19}F NMR (376 MHz) spectrum of compound **1** ($CDCl_3$).

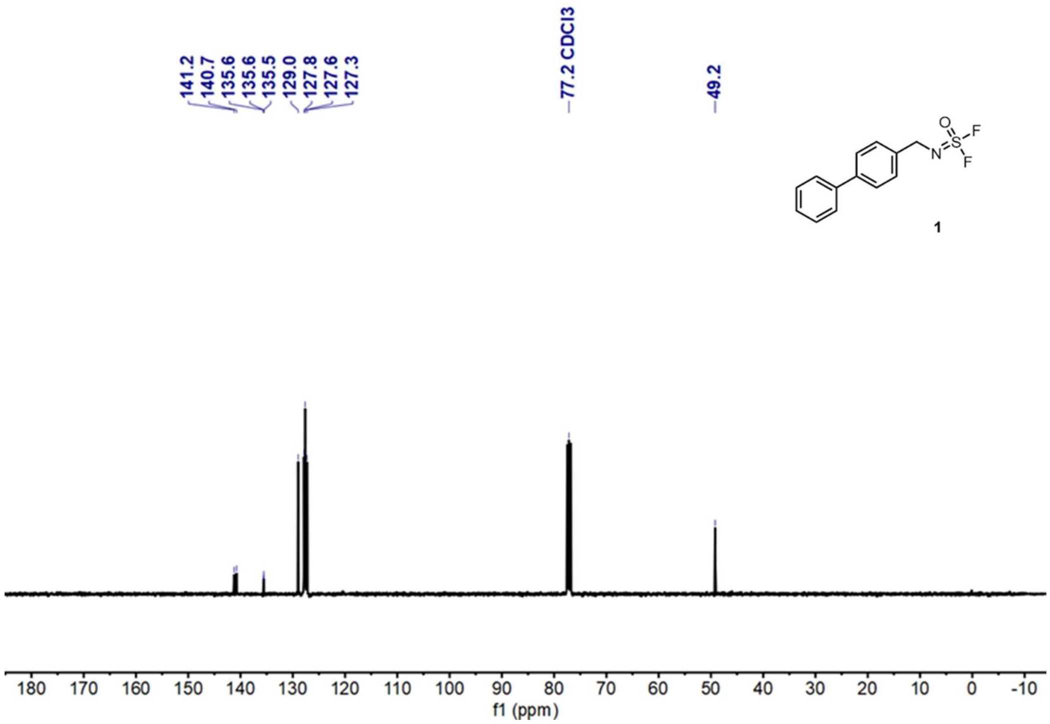

Figure S19. ^{13}C {^{1}H} NMR (101 MHz) spectrum of compound **1** ($CDCl_3$).

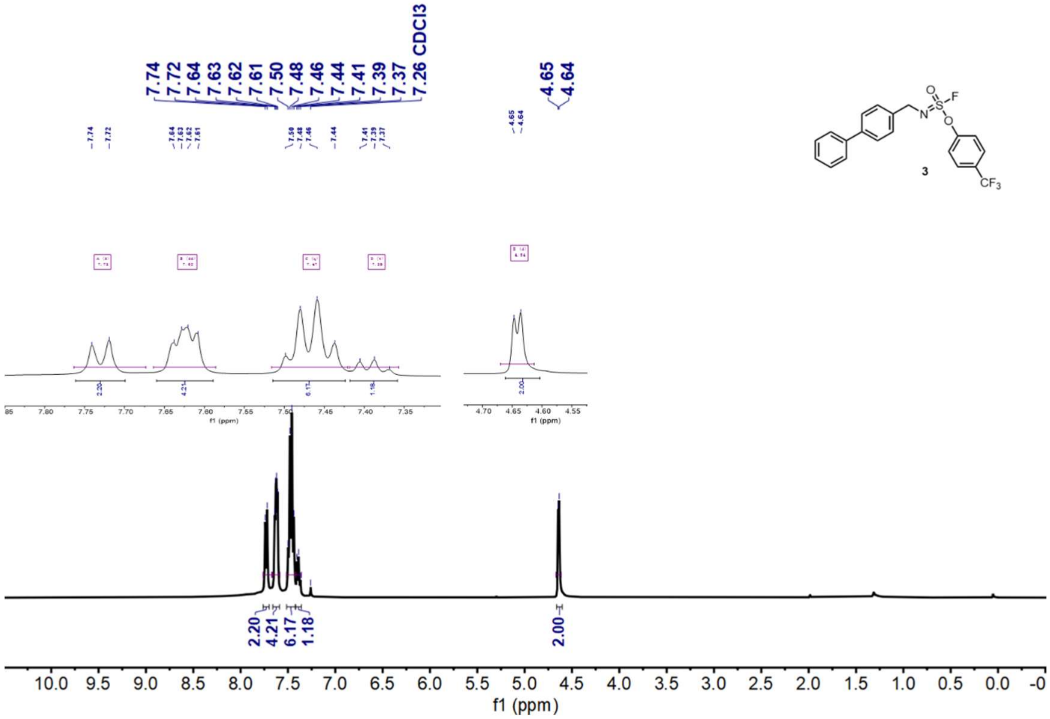

Figure S20. ^{1}H NMR (400 MHz) spectrum of compound **3** ($CDCl_3$).

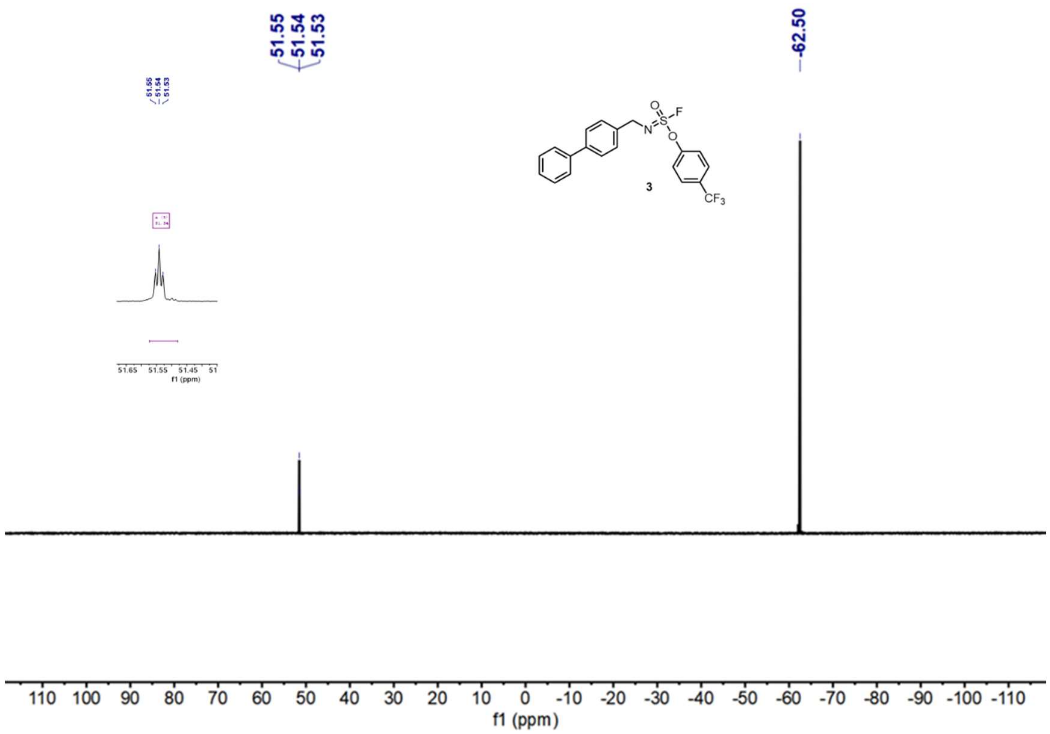

Figure S21. ^{19}F NMR (376 MHz) spectrum of compound **3** ($CDCl_3$).

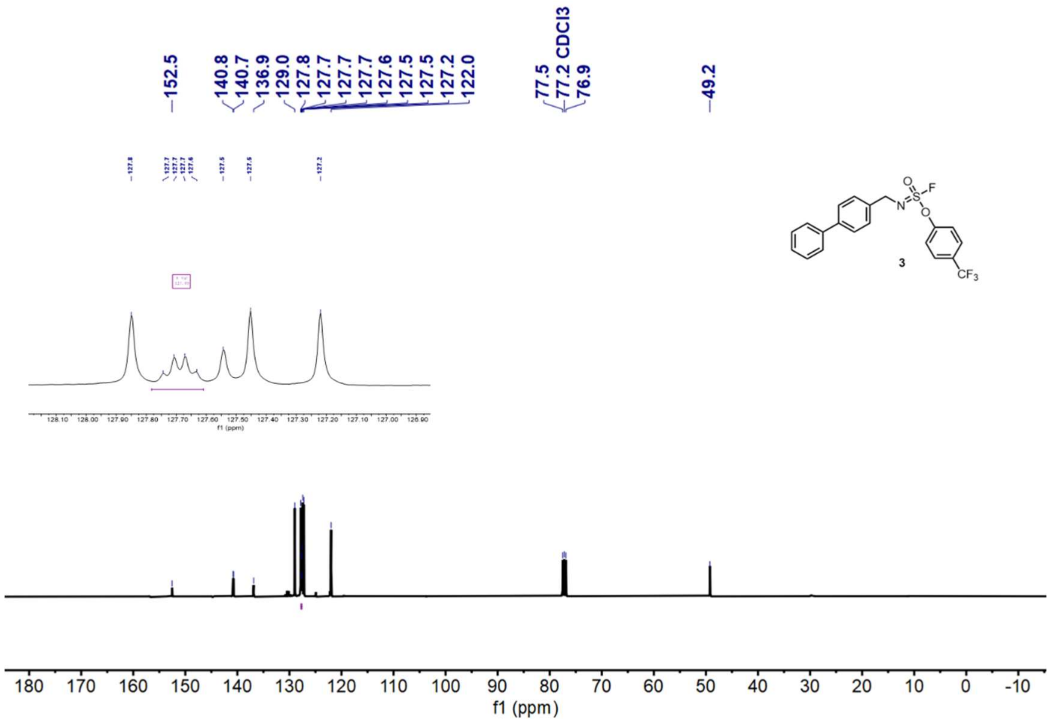

Figure S22. ^{13}C {^{1}H} NMR (101 MHz) spectrum of compound **3** ($CDCl_3$).

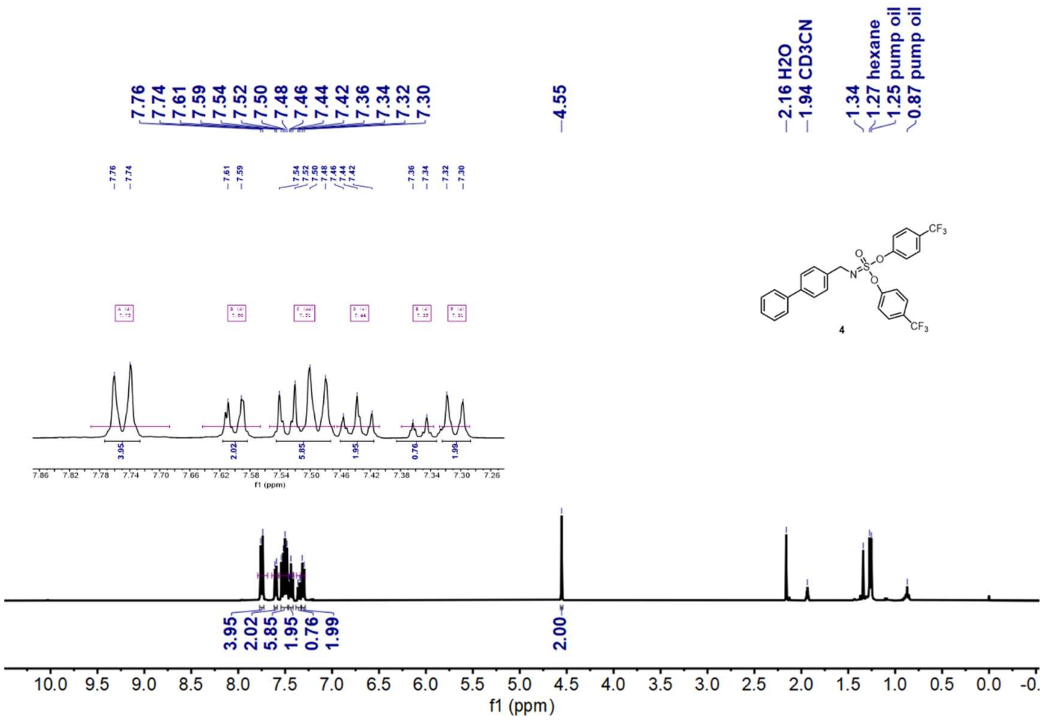

Figure S23. ^{1}H NMR (400 MHz) spectrum of compound **4** (CD_3CN).

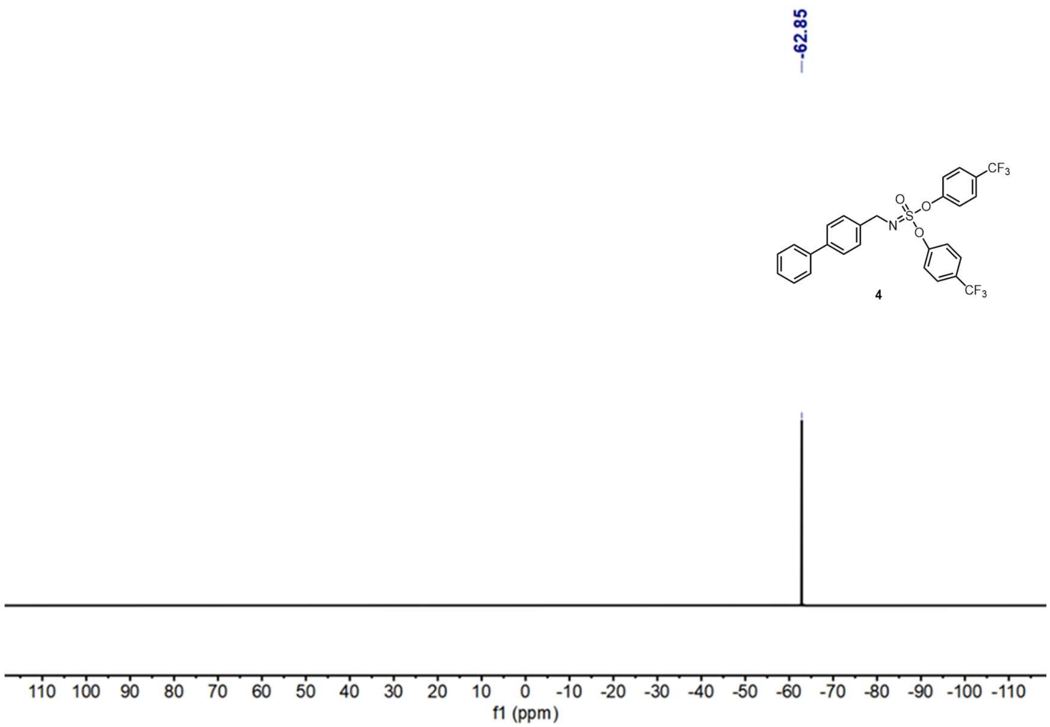

Figure S24. ^{19}F NMR (376 MHz) spectrum of compound **4** (CD_3CN).

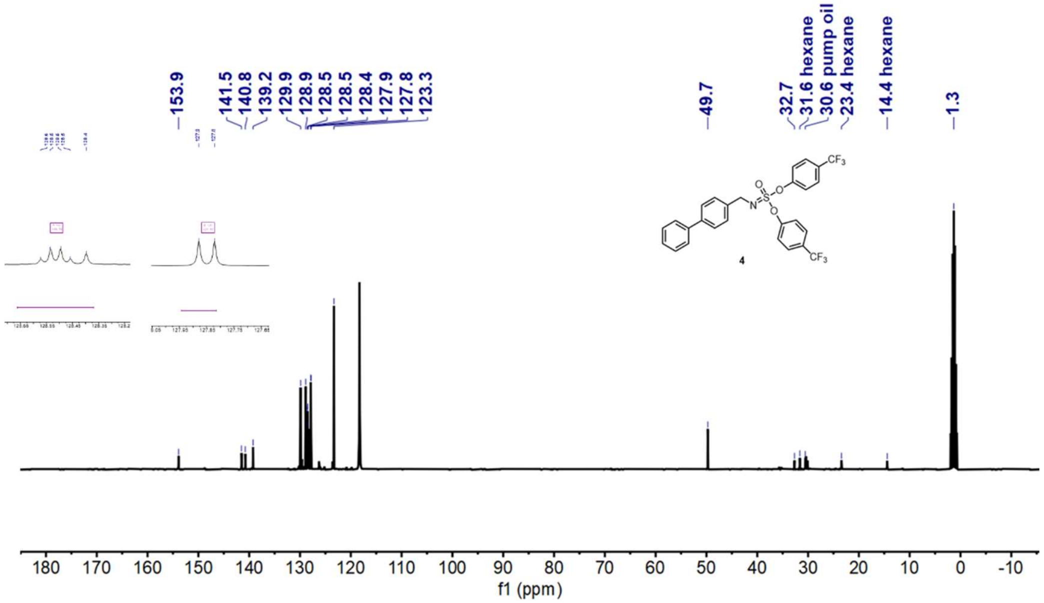

Figure S25. $^{13}C\ \{^{1}H\}$ NMR (101 MHz) spectrum of compound **4** (CD_3CN).

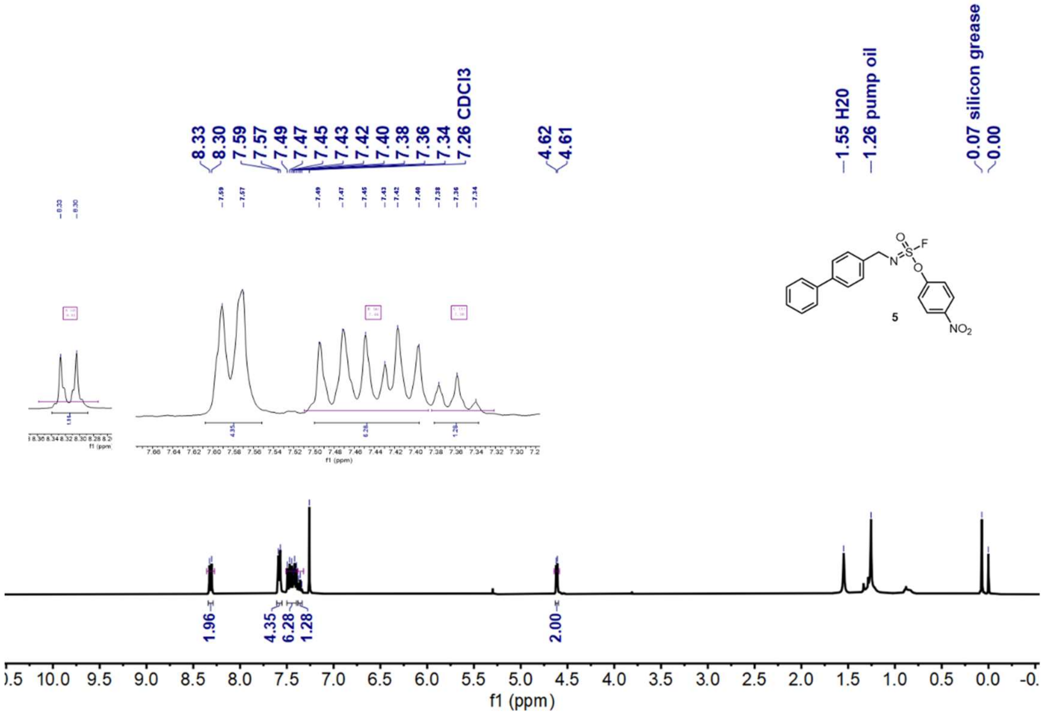

Figure S26. ^{1}H NMR (400 MHz) spectrum of compound **5** ($CDCl_3$).

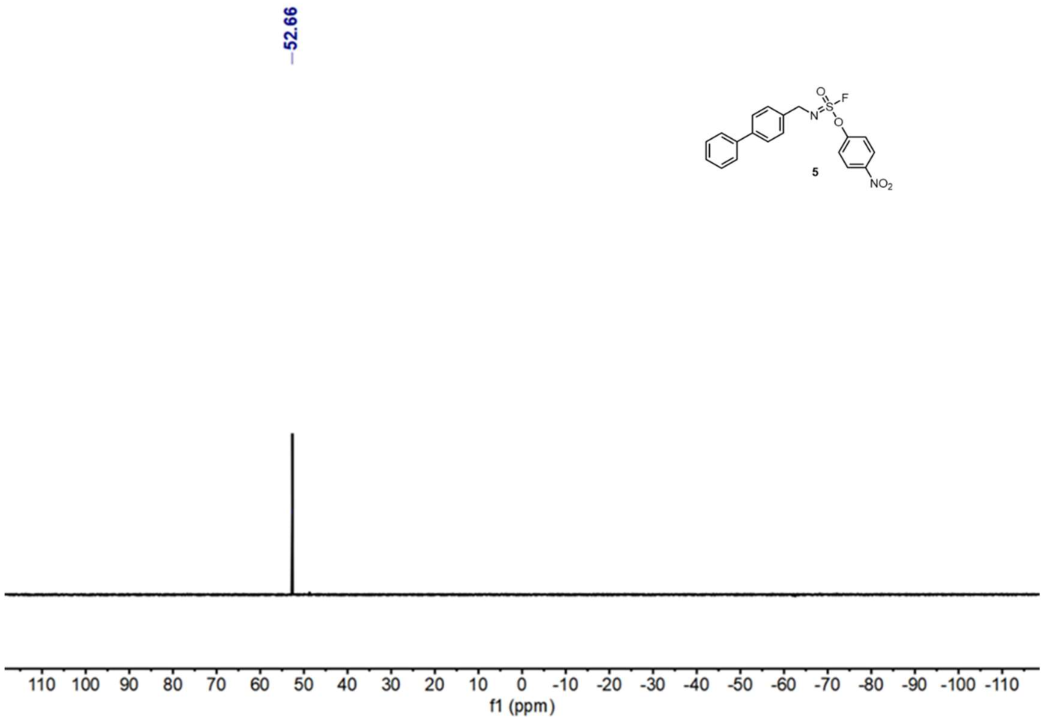

Figure S27. ^{19}F NMR (376 MHz) spectrum of compound **5** ($CDCl_3$).

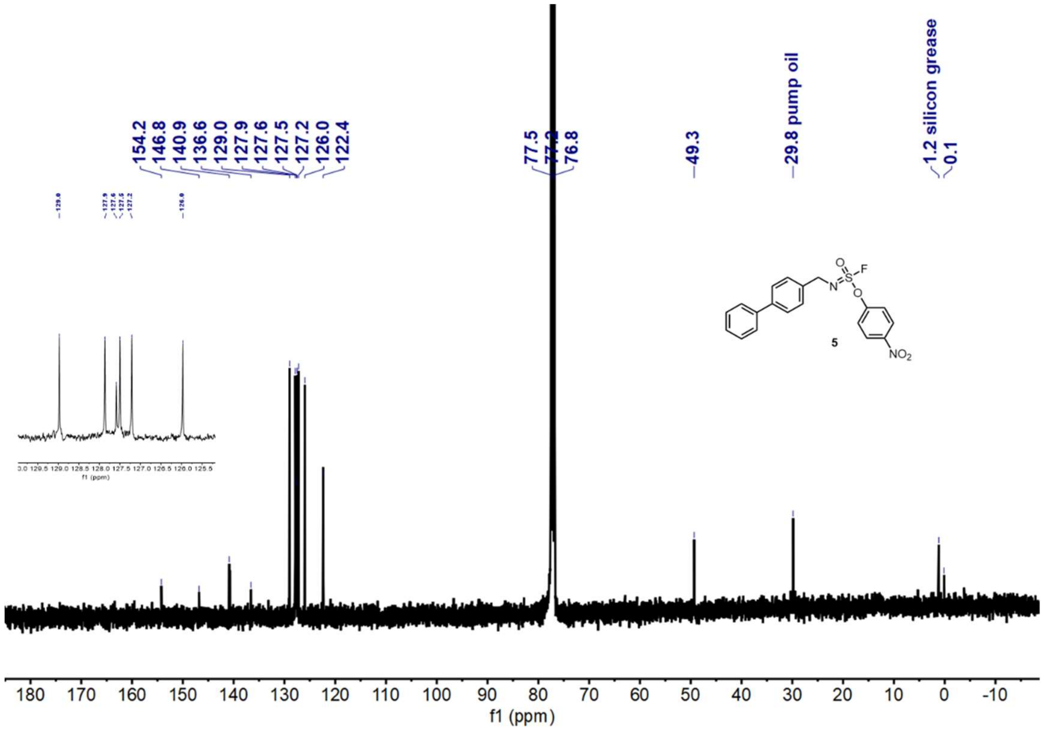

Figure S28. ^{13}C {^{1}H} NMR (101 MHz) spectrum of compound **5** ($CDCl_3$).

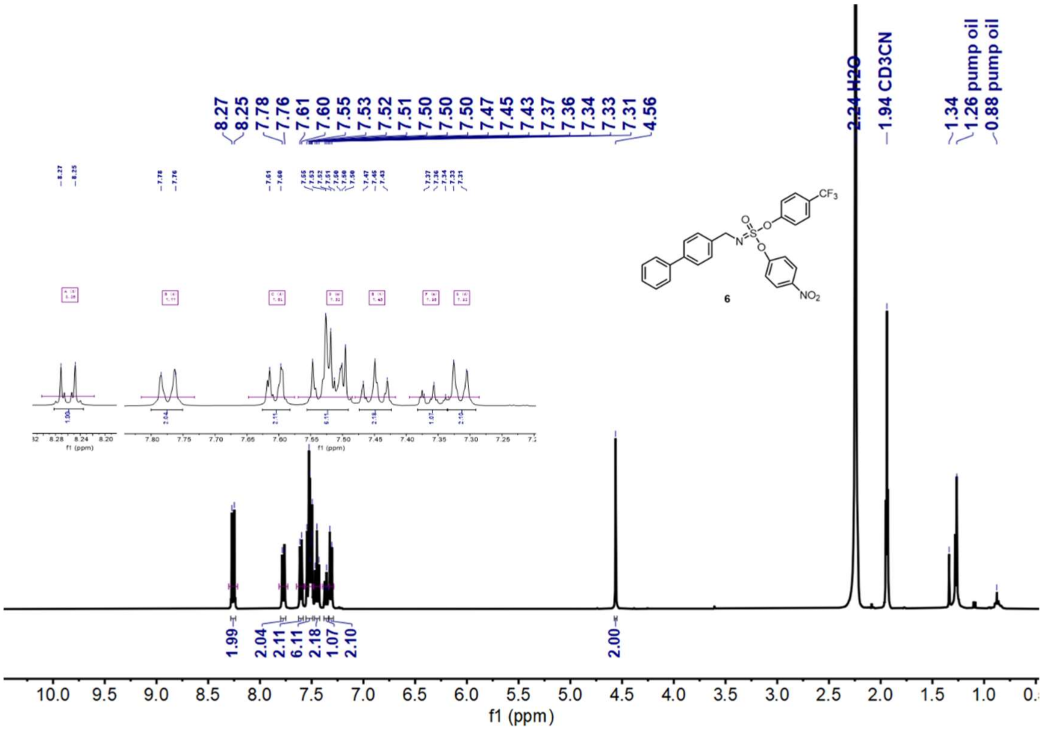

Figure S29. ^{1}H NMR (400 MHz) spectrum of compound **6** (CD_3CN).

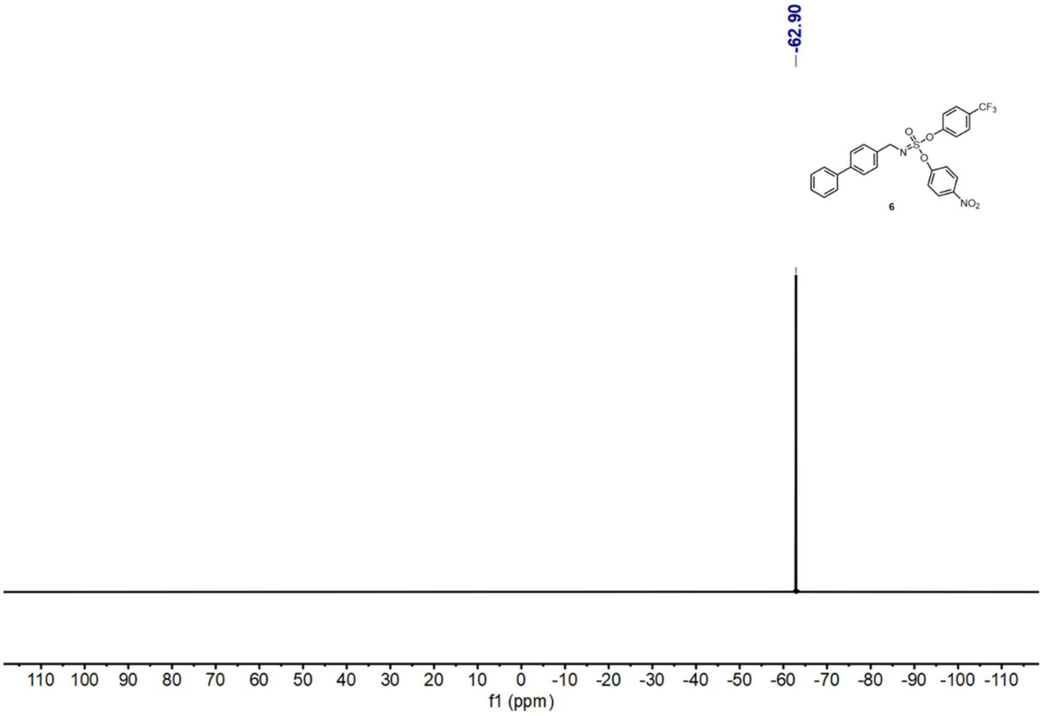

Figure S30. ^{19}F NMR (376 MHz) spectrum of compound **6** (CD_3CN).

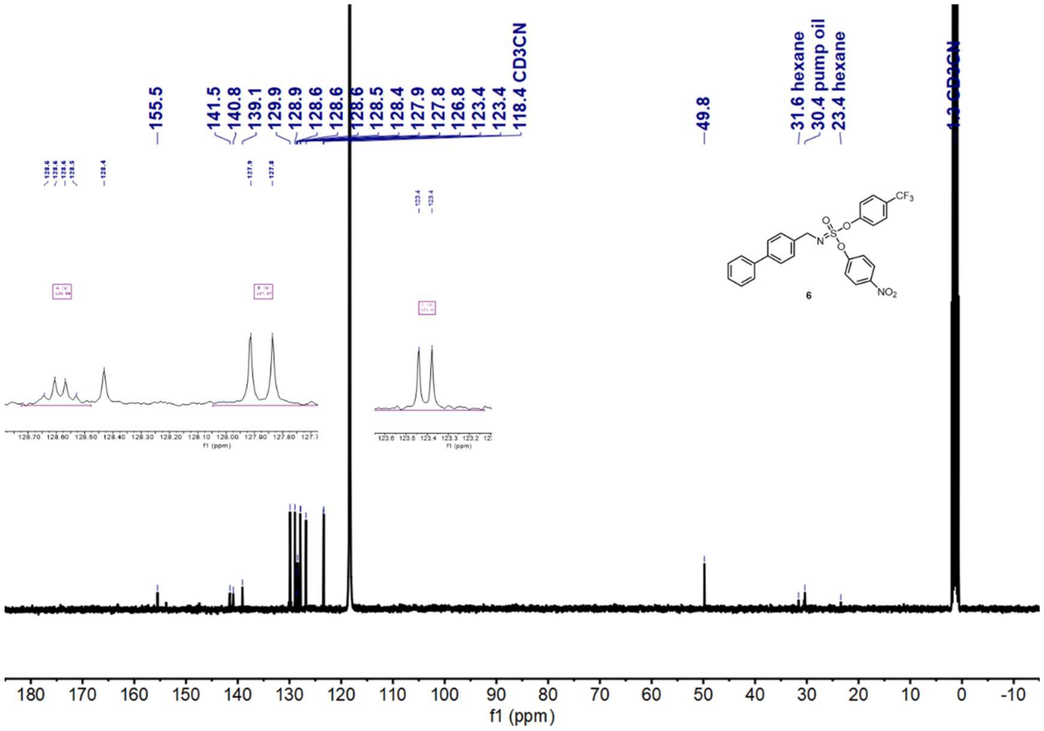

Figure S31. ^{13}C {^{1}H} NMR (101 MHz) spectrum of compound **6** (CD_3CN).

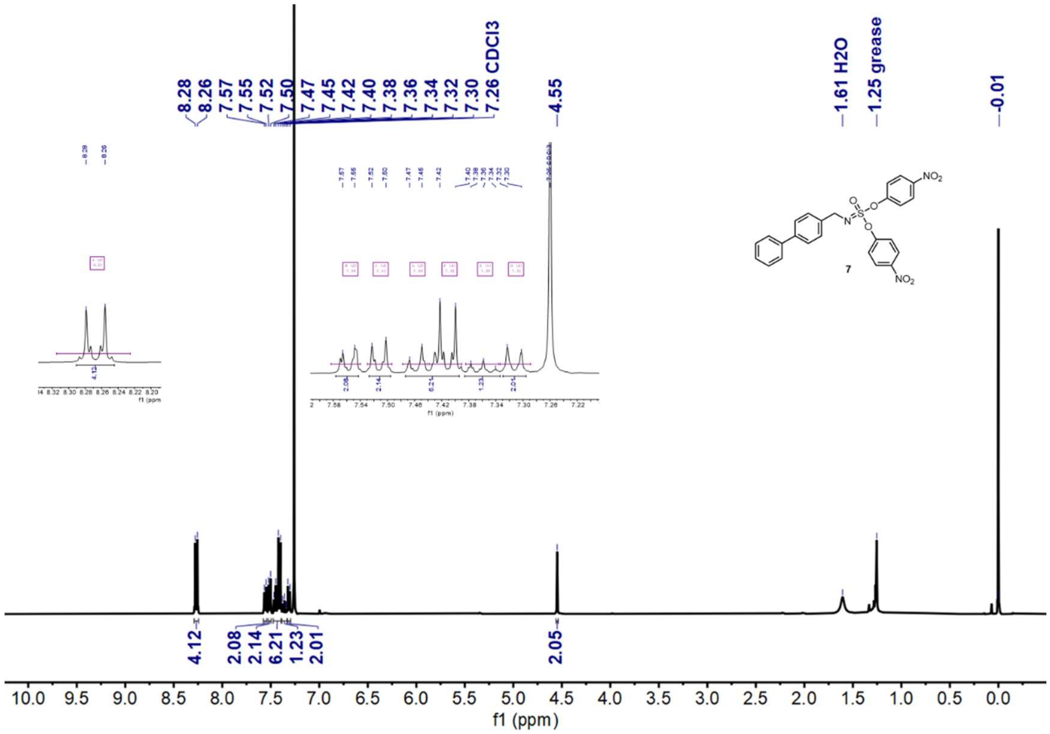

Figure S32. ^{1}H NMR (400 MHz) spectrum of compound **7** ($CDCl_3$).

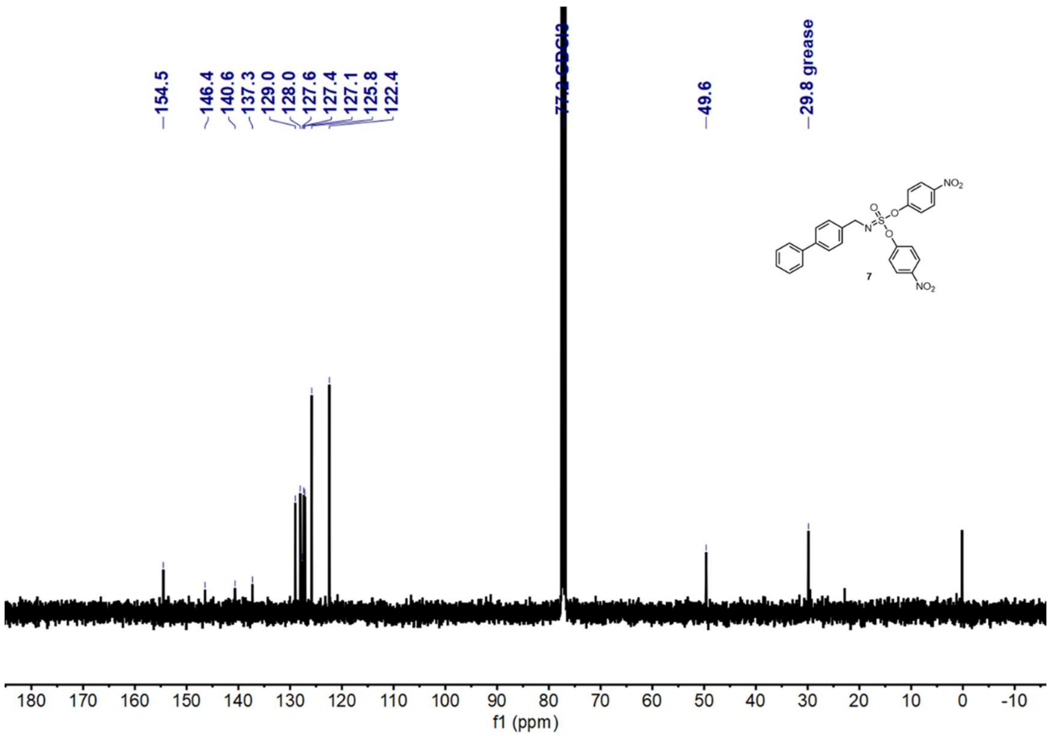

Figure S33. ^{13}C {^{1}H} NMR (101 MHz) spectrum of compound **7** ($CDCl_3$).

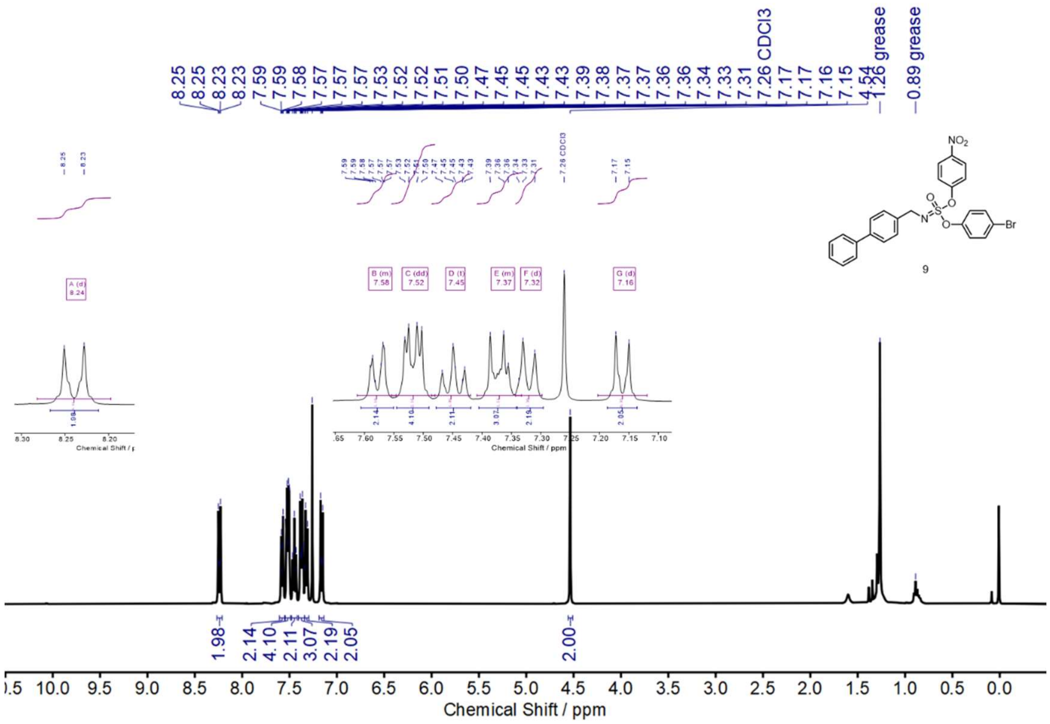

Figure S34. ^{1}H NMR (400 MHz) spectrum of compound **9** ($CDCl_3$).

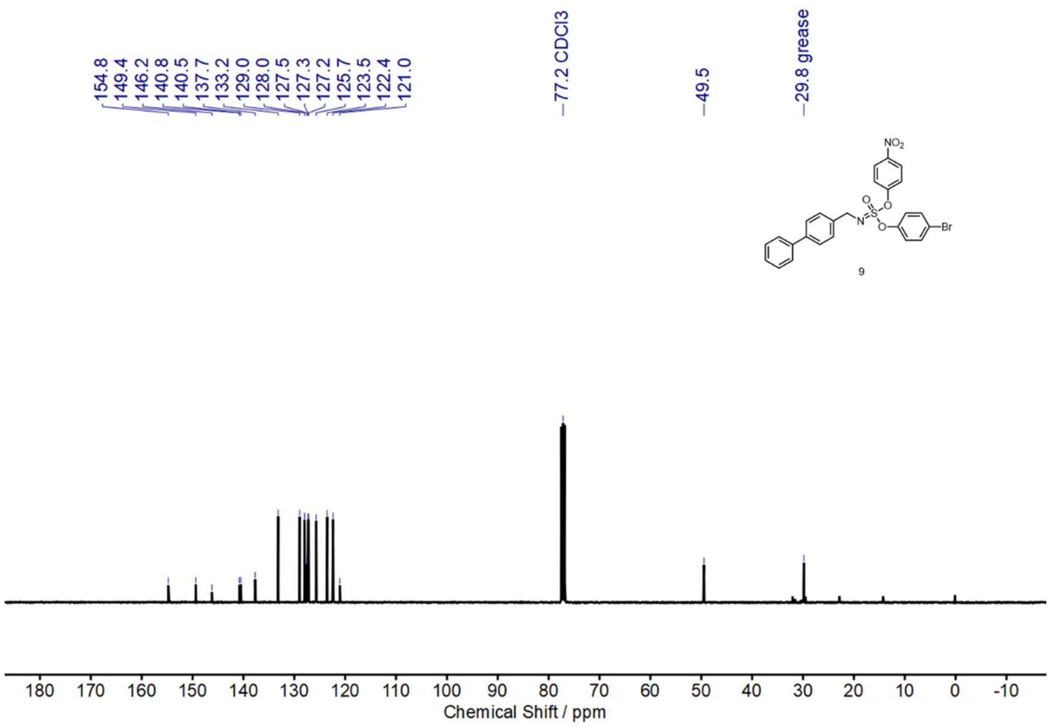

Figure S35. ^{13}C {^{1}H} NMR (101 MHz) spectrum of compound **9** ($CDCl_3$).

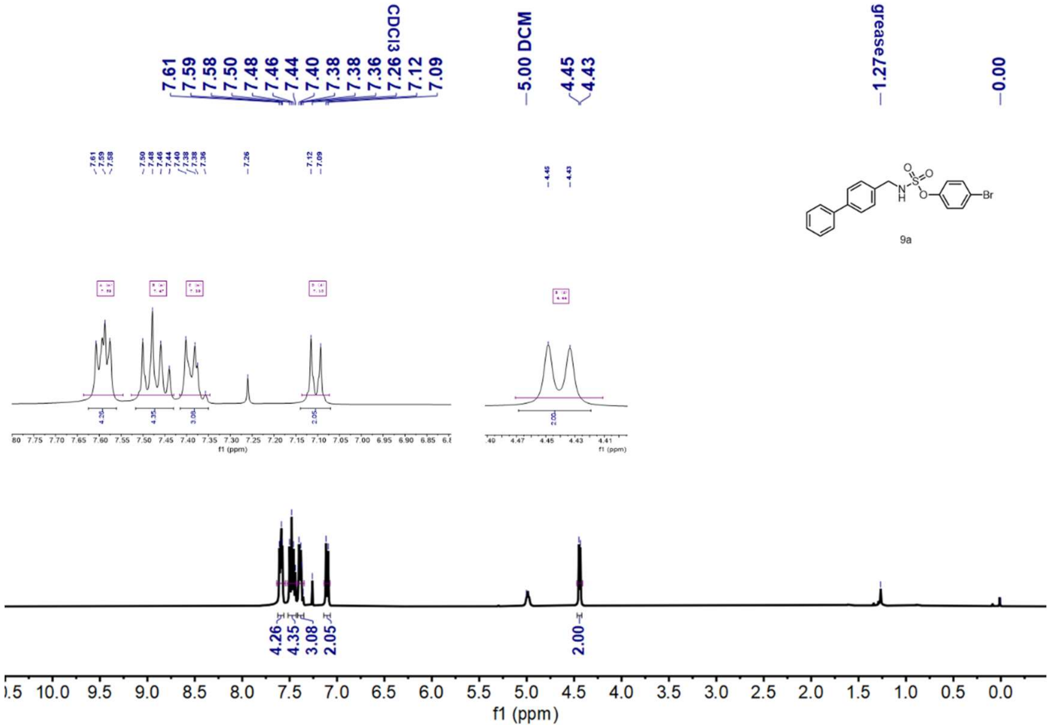

Figure S36. ^{1}H NMR (400 MHz) spectrum of compound **9a** ($CDCl_3$).

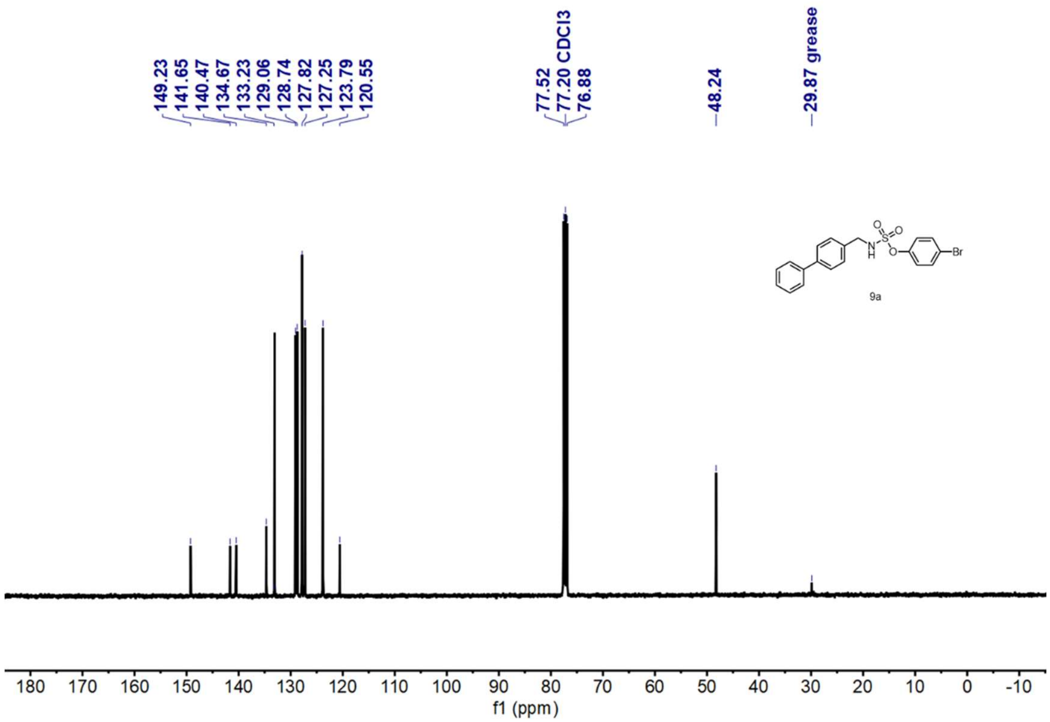

Figure S37. ^{13}C {^{1}H} NMR (101 MHz) spectrum of compound **9a** ($CDCl_3$).

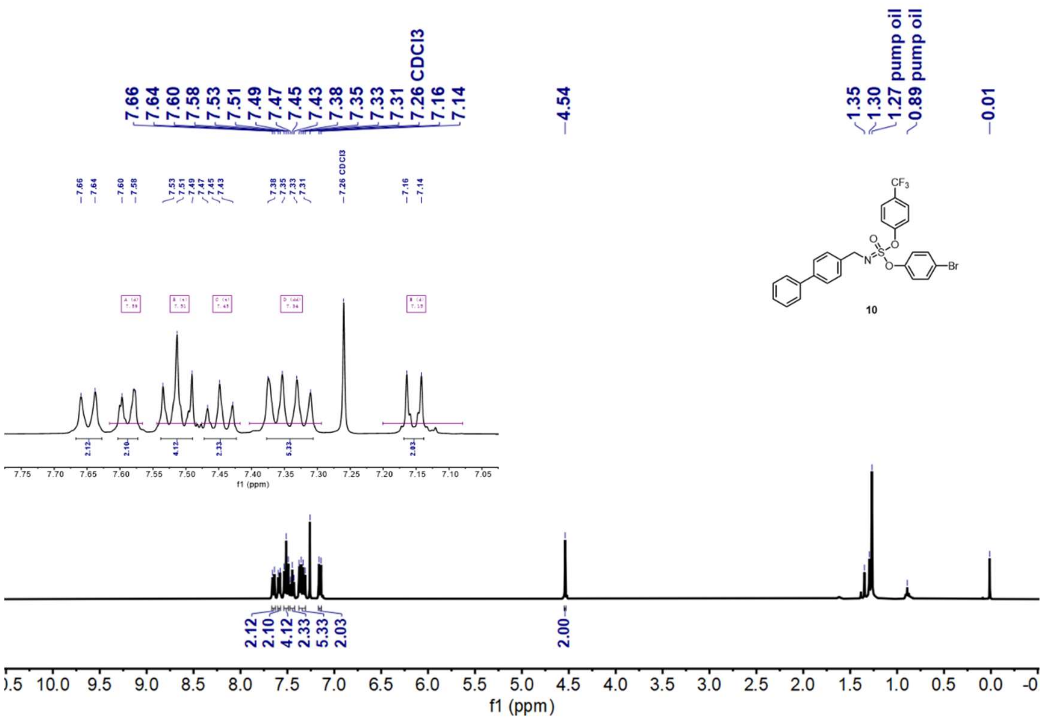

Figure S38. ^{1}H NMR (400 MHz) spectrum of compound **10** ($CDCl_3$).

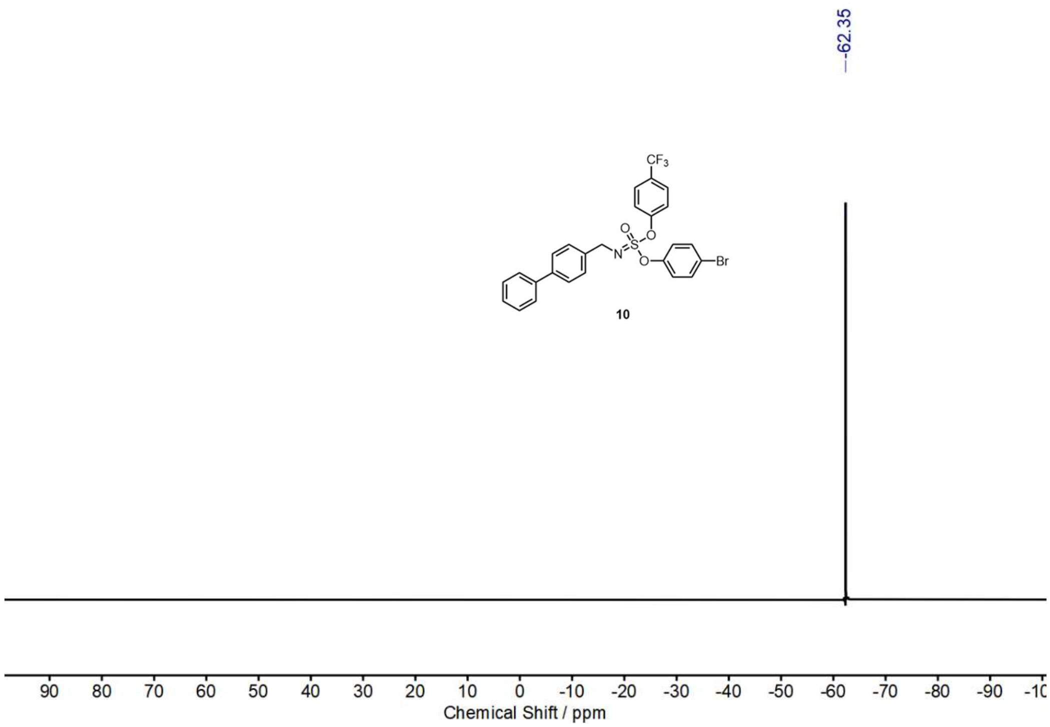

Figure S39. ^{19}F NMR (376 MHz) spectrum of compound **10** ($CDCl_3$).

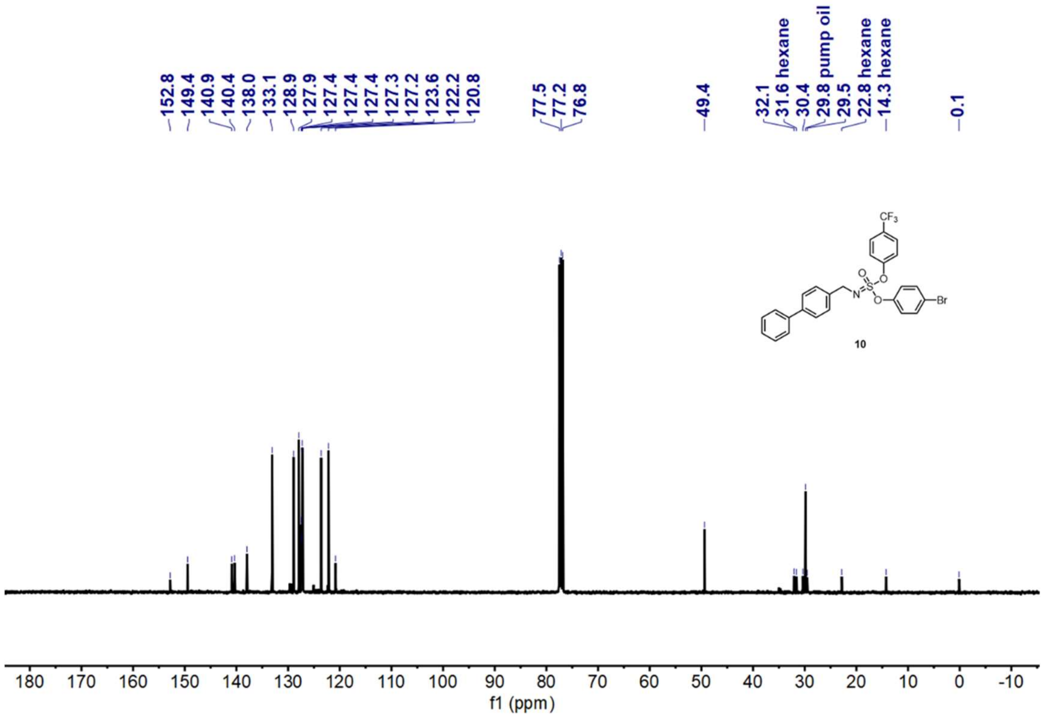

Figure S40. ^{13}C {^{1}H} NMR (101 MHz) spectrum of compound **10** ($CDCl_3$).

8. Chiral HPLC data

The two enantiomers of compound **3** were isolated into two compounds **(*R*)-3** and **(*S*)-3** by HPLC chiral resolution.

Enantiomeric excess (*ee*) value was calculated by using the equation in the following:

$$ee = \left(\frac{[R]-[S]}{[R]+[S]}\right) \times 100\% \qquad (7)$$

HPLC Condition: CHIRALPAK®IA column column, n-hexane/iPrOH = 95:5, flow rate: 0.5 mL/min, 40 ºC, UV detection wavelength: 254 nm, Inject volume: 5 μL, peak 1 =11.2 min, peak 2 = 12.4 min.

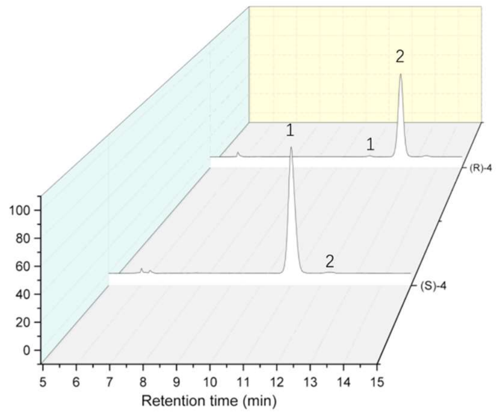

Figure S41. HPLC chromatograms of **(*S*)-3 and (*R*)-3.**

Table S12. Retention times and area (%) values for chromatograms of ***(S)*-3 and *(R)*-3.**

compound	Retention time (min)		Area (%)		*ee* (%)
	Peak 1	Peak 2	Peak 1	Peak 2	
**(*S*)-3**	11.013	12.295	99.057	0.943	98.1
**(*R*)-3**	11.316	12.557	99.115	0.885	98.2

HPLC Condition: CHIRALPAK®IA column column, n-hexane/iPrOH = 95:5, flow rate: 0.5 mL/min, 40 ºC, UV detection wavelength: 254 nm, Inject volume: 5 µL, peak 1 =22.0 min, peak 2 = 24.0 min.

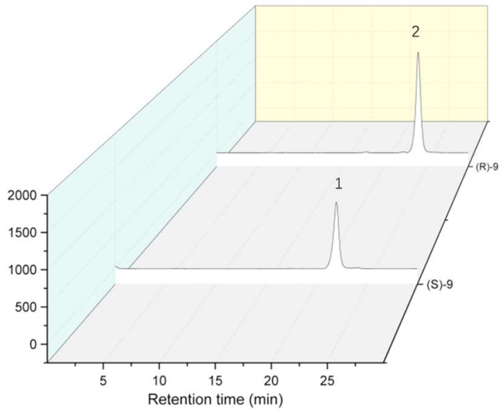

Figure S42. HPLC chromatograms of **(*S*)-10** and **(*R*)-10.**

Table S13. Retention times and area (%) values for chromatograms of ***(S)*-10 and *(R)*-10.**

compound	Retention time (min)		Area (%)		*ee* (%)
	Peak 1	Peak 2	Peak 1	Peak 2	
**(*S*)-10**	21.964	24.069	99.349	0.651	98.7
**(*R*)-10**	22.324	24.046	0.790	99.210	98.4

HPLC Condition: CHIRALPAK®IA column column, n-hexane/iPrOH = 95:5, flow rate: 0.3 mL/min, 35 ºC, UV detection wavelength: 254 nm, Inject volume: 5 µL, (*S*)-10 =26.1 min, (*R*)-10 = 27.8 min.

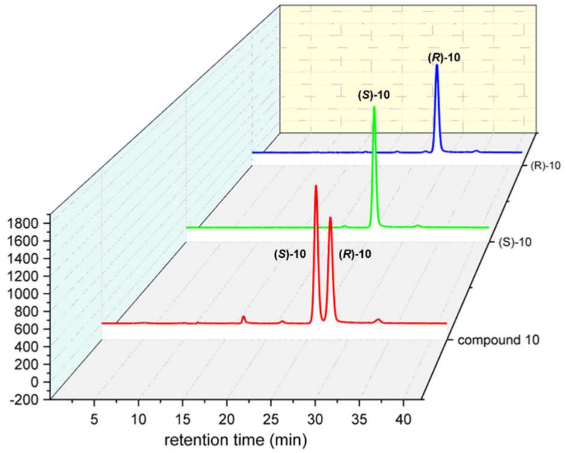

Figure S43. HPLC chromatograms of compound 10, **(*S*)-10** and **(*R*)-10.**

9. X-Ray crystallography

Single crystals of compound **(*S*)-3**, **(*R*)-10** and **9a** suitable for X-ray diffraction were selected and mounted in inert oil in cold gas stream and their X-ray diffraction intensity data was collected on a Rigaku XtaLAB FRX diffractometer equipped with the Hypix6000HE detector, using Cu Kα radiation (λ = 1.54184 Å) and Mo Kα radiation (λ = 0.71073 Å). Crystal was kept at the temperature listed in Table S11-S14 during data collection. By the use of Olex2, the structure was solved either (i) with the ShelXS[2] structure solution program using Direct Methods or (ii) with the ShelXT structure solution program using Direct Methods or Intrinsic Phasing, (iii) refined with the ShelXT refinement package using Least Squares minimization. The hydrogen atoms were set in calculated positions and refined as riding atoms with a common fixed isotropic thermal parameter. Some guest molecules were refined isotropically due to disorder that could not be modeled precisely. Distance restraints were also imposed on some disordered guest hexane molecules. Selected details of the data collection and structural refinement of each compound can be found within **Table S14–S16** and full details are available in the corresponding CIF files.

Table S14. Crystal data and structure refinement for **(*S*)-3**.

Crystallization Solvent	Acetonitrile / n-heptane
Empirical formula	$C_{20}H_{16.75}F_4NO_2S$
Formula weight	409.39
Temperature/K	100.00(10)
Crystal system	monoclic
Space group	$P2_1$
a/Å	8.0449(9)
b/Å	5.4032(6)
c/Å	40.253(5)
α/°	90
β/°	94.077(13)
γ/°	90
Volume/Å^3	1745.3(3)
Z	4
ρ_{calc}g/cm^3	1.558
μ/mm^{-1}	2.189
F(000)	840.0
Crystal size/mm^3	0.22 × 0.19 × 0.08
Radiation	CuKα (λ = 1.54184)
2Θ range for data collection/°	6.604 to 133.202

Index ranges	-8 ≤ h ≤ 9, -6 ≤ k ≤ 6, -47 ≤ l ≤ 47
Reflections collected	30284
Independent reflections	6019 [Rint = 0.2597, Rsigma = 0.1758]
Data/restraints/parameters	6019/510/489
Goodness-of-fit on F^2	1.730
Final R indexes [I>=2σ (I)]	R1 = 0.2129, wR2 = 0.5124
Final R indexes [all data]	R1 = 0.3007, wR2 = 0.5520
Largest diff. peak/hole / e $Å^{-3}$	0.94/-1.12
Flack parameter	-0.02(15)
CCDC number	2418873

Table S15. Crystal data and structure refinement for ***(R)*-10**.

Crystallization Solvent	Tetrahydrofuran
Empirical formula	$C_{26}H_{19}NO_3F_3SBr$
Formula weight	562.39
Temperature/K	100
Crystal system	monoclinic
Space group	$P2_1$
a/Å	14.1463(11)
b/Å	5.3290(2)
c/Å	17.1660(14)
α/°	90
β/°	112.928(9)
γ/°	90
Volume/$Å^3$	1191.83(16)
Z	2
$\rho_{calc}g/cm^3$	1.567
μ/mm^{-1}	3.641
F(000)	568.0
Crystal size/mm^3	0.19 × 0.02 × 0.02
Radiation	Cu Kα (λ = 1.54184)
2Θ range for data collection/°	5.59 to 136.612
Index ranges	-16 ≤ h ≤ 16, -6 ≤ k ≤ 3, -20 ≤ l ≤ 20
Reflections collected	14734
Independent reflections	2799 [R_{int} = 0.0710, R_{sigma} = 0.0402]
Data/restraints/parameters	2799/56/352

Goodness-of-fit on F^2	1.011
Final R indexes [I>=2σ (I)]	R_1 = 0.0725, wR_2 = 0.1888
Final R indexes [all data]	R_1 = 0.0941, wR_2 = 0.2161
Largest diff. peak/hole / e $Å^{-3}$	0.66/-0.86
Flack parameter	0.01(2)
CCDC number	2421104

Table S16. Crystal data and structure refinement for **9a**.

Crystallization Solvent	CH_2Cl_2 / *n*-hexane
Empirical formula	$C_{19}H_{16}BrNO_3S$
Formula weight	418.30
Temperature/K	293(2)
Crystal system	monoclinic
Space group	$P2_1/c$
a/Å	10.2218(2)
b/Å	38.3803(6)
c/Å	10.1453(2)
α/°	90
β/°	111.664(2)
γ/°	90
Volume/$Å^3$	3699.02(13)
Z	8
$\rho_{calc}g/cm^3$	1.502
μ/mm^{-1}	4.233
F(000)	1696.0
Crystal size/mm^3	0.6 × 0.15 × 0.03
Radiation	Cu Kα (λ = 1.54184)
2Θ range for data collection/°	9.216 to 149.608
Index ranges	$-12 \leq h \leq 12$, $-47 \leq k \leq 39$, $-11 \leq l \leq 12$
Reflections collected	19642
Independent reflections	6929 [R_{int} = 0.0354, R_{sigma} = 0.0296]
Data/restraints/parameters	6929/0/451
Goodness-of-fit on F^2	1.073
Final R indexes [I>=2σ (I)]	R_1 = 0.0694, wR_2 = 0.2095
Final R indexes [all data]	R_1 = 0.0790, wR_2 = 0.2166
Largest diff. peak/hole / e $Å^{-3}$	0.64/-0.95
CCDC number	2425236

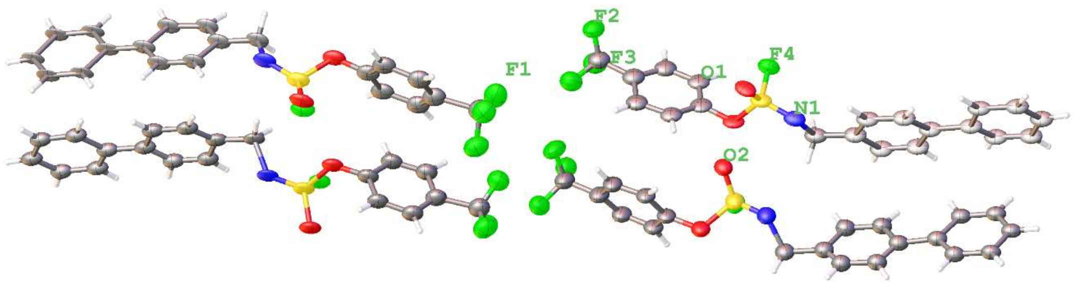

Figure S44. The X-ray crystal structure of compound **(S)-3**. Displacement ellipsoids are drawn at the 50% probability level.

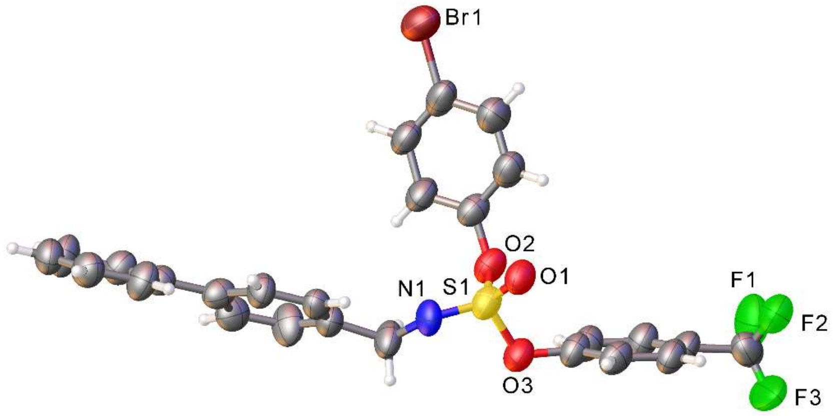

Figure S45. The X-ray crystal structure of compound **(*R*)-10**. Displacement ellipsoids are drawn at the 50% probability level

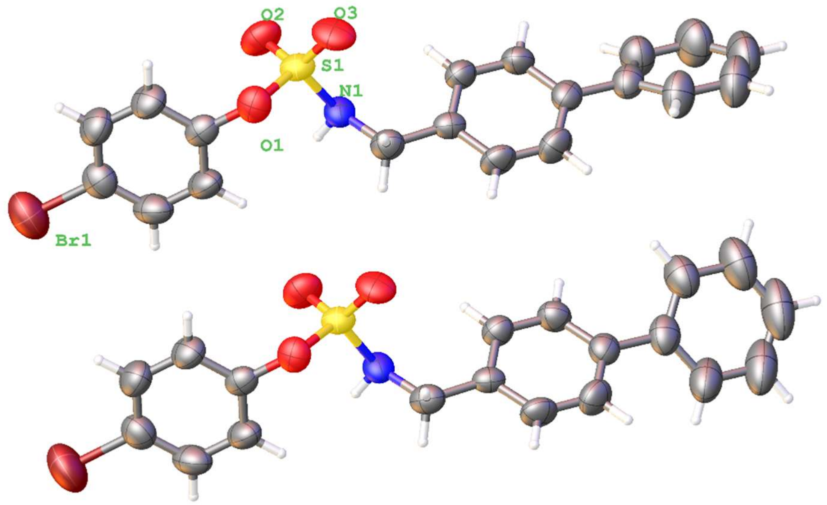

Figure S46. The X-ray crystal structure of compound **9a**. Displacement ellipsoids are drawn at the 50% probability level

10. DFT-computed theoretical values of activation enthalpies for all reactions

To evaluate the influence of basis set choice on theoretical calculations, we compared experimental activation enthalpies ($\Delta H^{\ddagger}$) with values calculated using three basis sets of increasing levels of theory: 6-311+G(d,p), def2-TZVP, and def2-QZVPP.[3] The results of these comparisons are summarized in **Table S17**. The 6-311+G(d,p) basis set shows the lowest average deviation (0.88 kcal) providing the most accurate reproduction of experimental data, while def2tzvp and def2qzvpp has the largest deviation (4.16 kcal and 4.55 kcal respectively). Interestingly, despite being a relatively smaller basis set, 6-311+G(d,p) performs remarkably well for these reactions, yielding $\Delta H^{\ddagger}$ values that closely match the experimental results.

Table S17. DFT-Calculated $\Delta H^{\ddagger}$ (kcal/mol)

Reactions	6-311+G(d,p)	def2-TZVP	def2-QZVPP
**I**	2.29	6.68	7.24
**II**	12.49	16.66	16.84
**IIIa**	8.37	13.02	12.85
**IIIb**	11.62	15.96	16.63
**IV**	14.19	18.45	19.17

10.1 Computational details

All calculations were done using Gaussian 16 software at the ωB97XD/6–311+G(d,p) level of theory. The SMD solvent model was used to represent acetonitrile. Frequency calculations were done for all stationary points to confirm them as either a minimum or a TS. Full Cartesian coordinates and energies of all computed structures are given below.

10.1.1 Electronic energies, entropy corrections, calculated free energies (all in Hartree) and optimized Cartesian Coordinates for all stationary points on the reaction profiles.

Reaction I

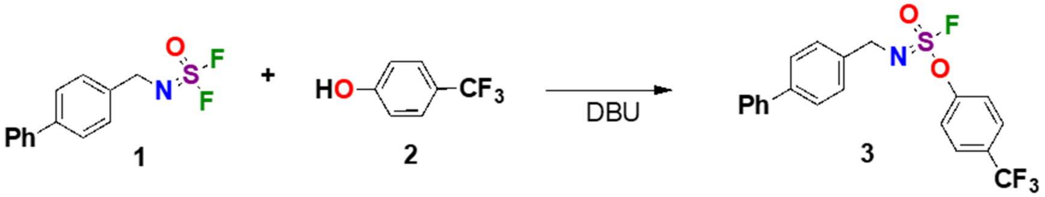

Reactant complex

Thermal correction to Energy: 0.340911

Thermal correction to Enthalpy: 0.341855

Thermal correction to Gibbs Free Energy: 0.255550

Sum of electronic and zero-point Energies: -1873.464435

Sum of electronic and thermal Energies: -1873.438653

Sum of electronic and thermal Enthalpies: -1873.437709

Sum of electronic and thermal Free Energies: -1873.524014

Center Number	X	Y	Z
C1	5.8190	-0.5721	0.3778
C2	4.4488	-0.4945	0.5988
C3	3.5832	-1.4640	0.0809
C4	4.1308	-2.5147	-0.6632
C5	5.5011	-2.5921	-0.8853
C6	6.3513	-1.6205	-0.3664
H7	6.4715	0.1949	0.7815
H8	4.0462	0.3394	1.1622
H9	3.4822	-3.2886	-1.0600
H10	5.9055	-3.4180	-1.4607
H11	7.4202	-1.6800	-0.5405
C12	2.1176	-1.3792	0.3128
C13	1.2101	-1.7408	-0.6852
C14	1.6055	-0.9279	1.5337
C15	-0.1610	-1.6682	-0.4693
H16	1.5743	-2.0672	-1.6535
C17	0.2371	-0.8491	1.7464
H18	2.2824	-0.6416	2.3314
C19	-0.6621	-1.2263	0.7499
H20	-0.8438	-1.9445	-1.2646
H21	-0.1372	-0.4925	2.7013
C22	-2.1393	-1.1717	1.0516
H23	-2.3787	-0.2278	1.5475
H24	-2.4031	-1.9904	1.7290

N25	-2.9161	-1.2624	-0.1846
S26	-4.3570	-1.4628	-0.3203
O27	-5.0253	-1.3653	-1.5754
F28	-4.8360	-2.8851	0.3113
F29	-5.2260	-0.6574	0.7604
C30	-0.2524	1.8566	-1.3170
C31	0.3850	2.3205	-0.1587
C32	-0.3994	2.6238	0.9632
C33	-1.7679	2.4639	0.9354
C34	-2.4650	1.9908	-0.2318
C35	-1.6205	1.6921	-1.3567
H36	0.3382	1.6134	-2.1952
H37	0.0764	2.9855	1.8701
H38	-2.3578	2.6924	1.8190
H39	-2.0937	1.3131	-2.2581
O40	-3.7220	1.8425	-0.2598
C41	1.8374	2.5832	-0.1491
F42	2.1657	3.8682	-0.5060
F43	2.5328	1.8042	-1.0071
F44	2.4055	2.4177	1.0693

TS1

Imaginary Frequency: -96.0632 cm^{-1}

Thermal correction to Energy: 0.340043

Thermal correction to Enthalpy: 0.340987

Thermal correction to Gibbs Free Energy: 0.258933

Sum of electronic and zero-point Energies: -1873.459389

Sum of electronic and thermal Energies: -1873.435010

Sum of electronic and thermal Enthalpies: -1873.434066

Sum of electronic and thermal Free Energies: -1873.516119

Center Number	X	Y	Z
C1	4.9243	-1.1563	-0.2037
C2	3.6035	-0.9988	0.1993
C3	2.7016	-2.0656	0.1330
C4	3.1596	-3.2970	-0.3470
C5	4.4810	-3.4557	-0.7494
C6	5.3687	-2.3859	-0.6803
H7	5.6049	-0.3130	-0.1526
H8	3.2633	-0.0327	0.5544
H9	2.4814	-4.1427	-0.3926
H10	4.8192	-4.4202	-1.1128
H11	6.3989	-2.5099	-0.9958
C12	1.2889	-1.8819	0.5528
C13	0.2434	-2.4772	-0.1568
C14	0.9659	-1.0821	1.6536
C15	-1.0808	-2.2624	0.2062
H16	0.4617	-3.0920	-1.0238
C17	-0.3566	-0.8677	2.0118
H18	1.7561	-0.6157	2.2325
C19	-1.3977	-1.4418	1.2842
H20	-1.8725	-2.7178	-0.3792
H21	-0.5837	-0.2228	2.8551
C22	-2.8251	-1.1539	1.6895
H23	-2.8798	-0.1699	2.1544
H24	-3.1450	-1.8903	2.4328
N25	-3.8102	-1.2642	0.6250
S26	-4.0268	-0.4048	-0.5768
O27	-3.3285	-0.2527	-1.8280
F28	-5.3326	-1.2752	-1.1356
F29	-5.0681	0.8424	-0.4033
C30	0.6081	1.4544	-0.9990
C31	1.1571	2.3965	-0.1282
C32	0.3431	2.9544	0.8647
C33	-0.9763	2.5728	0.9863
C34	-1.5598	1.6011	0.1231
C35	-0.7157	1.0697	-0.8886
H36	1.2226	1.0093	-1.7741

H37	0.7515	3.6920	1.5495
H38	-1.5982	3.0007	1.7660
H39	-1.1159	0.3302	-1.5679
O40	-2.7945	1.2601	0.3062
C41	2.5706	2.8316	-0.2372
F42	2.6999	4.1682	-0.4628
F43	3.2446	2.2232	-1.2315
F44	3.2891	2.6058	0.8969

Intermediate

Thermal correction to Energy: 0.340988

Thermal correction to Enthalpy: 0.341932

Thermal correction to Gibbs Free Energy: 0.258590

Sum of electronic and zero-point Energies: -1873.461144

Sum of electronic and thermal Energies: -1873.436207

Sum of electronic and thermal Enthalpies: -1873.435262

Sum of electronic and thermal Free Energies: -1873.518605

Center Number	X	Y	Z
C1	5.3075	-0.6932	-0.1872
C2	3.9715	-0.6329	0.1921
C3	3.1526	-1.7643	0.1160
C4	3.7107	-2.9596	-0.3498
C5	5.0468	-3.0206	-0.7293
C6	5.8509	-1.8875	-0.6507
H7	5.9227	0.1983	-0.1278
H8	3.5562	0.3070	0.5370
H9	3.0994	-3.8545	-0.4016
H10	5.4619	-3.9587	-1.0822
H11	6.8929	-1.9351	-0.9478
C12	1.7230	-1.6904	0.5131

C13	0.7404	-2.3830	-0.1990
C14	1.3188	-0.9087	1.6001
C15	-0.6005	-2.2829	0.1497
H16	1.0209	-2.9853	-1.0570
C17	-0.0213	-0.8104	1.9458
H18	2.0585	-0.3677	2.1808
C19	-1.0001	-1.4845	1.2177
H20	-1.3479	-2.8027	-0.4395
H21	-0.3129	-0.1793	2.7802
C22	-2.4570	-1.3260	1.5995
H23	-2.6109	-0.3384	2.0378
H24	-2.6920	-2.0553	2.3818
N25	-3.3941	-1.6102	0.5218
S26	-3.9430	-0.6080	-0.4818
O27	-3.5859	-0.3145	-1.8618
F28	-5.1388	-1.8155	-0.9887
F29	-5.3204	0.2248	-0.0543
C30	0.2292	1.4095	-1.0357
C31	0.6514	2.4026	-0.1604
C32	-0.2313	2.8877	0.8067
C33	-1.5103	2.3762	0.8949
C34	-1.9443	1.3641	0.0204
C35	-1.0578	0.8958	-0.9577
H36	0.9035	1.0207	-1.7895
H37	0.0837	3.6633	1.4969
H38	-2.1970	2.7382	1.6514
H39	-1.3617	0.1214	-1.6454
O40	-3.1990	0.9396	0.2210
C41	2.0300	2.9664	-0.2289
F42	2.0331	4.3071	-0.4324
F43	2.7737	2.4344	-1.2139
F44	2.7271	2.7792	0.9194

TS2

Imaginary Frequency: -141.5789 cm^{-1}

Thermal correction to Energy: 0.340260

Thermal correction to Enthalpy: 0.341204

Thermal correction to Gibbs Free Energy: 0.258496

Sum of electronic and zero-point Energies: -1873.460111

Sum of electronic and thermal Energies: -1873.435433

Sum of electronic and thermal Enthalpies: -1873.434489

Sum of electronic and thermal Free Energies: -1873.517197

Center Number	X	Y	Z
C1	5.5851	-0.4199	0.1664
C2	4.2214	-0.4400	0.4349
C3	3.4428	-1.5564	0.1117
C4	4.0715	-2.6538	-0.4870
C5	5.4352	-2.6339	-0.7567
C6	6.1982	-1.5165	-0.4317
H7	6.1682	0.4595	0.4185
H8	3.7548	0.4289	0.8844
H9	3.4929	-3.5385	-0.7310
H10	5.9036	-3.4975	-1.2166
H11	7.2620	-1.5006	-0.6427
C12	1.9840	-1.5742	0.3951
C13	1.0861	-2.1855	-0.4862
C14	1.4660	-0.9727	1.5451
C15	-0.2779	-2.1923	-0.2287
H16	1.4536	-2.6426	-1.3990
C17	0.1003	-0.9790	1.7998
H18	2.1342	-0.4956	2.2542
C19	-0.7894	-1.5851	0.9165
H20	-0.9546	-2.6500	-0.9417
H21	-0.2779	-0.4925	2.6936
C22	-2.2734	-1.5751	1.2176
H23	-2.4986	-0.7322	1.8761
H24	-2.5372	-2.4862	1.7649

N25	-3.0595	-1.5558	-0.0151
S26	-4.0689	-0.5034	-0.3727
O27	-4.3172	-0.0140	-1.7078
F28	-5.3977	-2.0195	-0.8126
F29	-5.3333	-0.1831	0.6345
C30	-0.1273	1.5054	-1.1534
C31	0.4518	2.2728	-0.1511
C32	-0.2843	2.6048	0.9860
C33	-1.5800	2.1452	1.1258
C34	-2.1560	1.3571	0.1263
C35	-1.4333	1.0527	-1.0256
H36	0.4344	1.2481	-2.0432
H37	0.1578	3.2071	1.7715
H38	-2.1556	2.3715	2.0154
H39	-1.8668	0.4521	-1.8136
O40	-3.4295	0.9476	0.3730
C41	1.8457	2.7906	-0.2992
F42	1.8752	4.0892	-0.6911
F43	2.5646	2.1111	-1.2089
F44	2.5381	2.7445	0.8586

Product complex

Thermal correction to Energy: 0.341730

Thermal correction to Enthalpy: 0.342674

Thermal correction to Gibbs Free Energy: 0.255600

Sum of electronic and zero-point Energies: -1873.481473

Sum of electronic and thermal Energies: -1873.455522

Sum of electronic and thermal Enthalpies: -1873.454578

Sum of electronic and thermal Free Energies: -1873.541652

Center Number	X	Y	Z

C1	5.5975	-0.7229	0.0541
C2	4.2352	-0.6546	0.3226
C3	3.3795	-1.6998	-0.0412
C4	3.9283	-2.8167	-0.6808
C5	5.2905	-2.8850	-0.9501
C6	6.1311	-1.8380	-0.5844
H7	6.2421	0.1018	0.3392
H8	3.8312	0.2273	0.8068
H9	3.2877	-3.6478	-0.9562
H10	5.6969	-3.7628	-1.4411
H11	7.1939	-1.8914	-0.7944
C12	1.9231	-1.6227	0.2432
C13	0.9841	-2.1219	-0.6648
C14	1.4506	-1.0390	1.4215
C15	-0.3769	-2.0378	-0.4054
H16	1.3180	-2.5637	-1.5978
C17	0.0885	-0.9475	1.6757
H18	2.1527	-0.6551	2.1535
C19	-0.8420	-1.4443	0.7662
H20	-1.0846	-2.4165	-1.1346
H21	-0.2539	-0.4784	2.5931
C22	-2.3122	-1.3653	1.0954
H23	-2.5026	-0.5026	1.7387
H24	-2.6119	-2.2681	1.6480
N25	-3.1593	-1.3327	-0.1064
S26	-3.9354	-0.2218	-0.6835
O27	-4.9312	-0.4805	-1.6776
F28	-3.1161	-3.9712	2.6319
F29	-4.6963	0.6048	0.5127
C30	0.4598	1.6096	-0.9455
C31	0.5033	2.3312	0.2402
C32	-0.6602	2.6053	0.9559
C33	-1.8837	2.1668	0.4761
C34	-1.9074	1.4496	-0.7103
C35	-0.7620	1.1597	-1.4282
H36	1.3661	1.3904	-1.4953
H37	-0.6174	3.1681	1.8811

H38	-2.8002	2.3837	1.0103
H39	-0.8257	0.5931	-2.3485
O40	-3.1335	1.0603	-1.2663
C41	1.8083	2.8382	0.7807
F42	1.7992	4.1799	0.9365
F43	2.8509	2.5510	-0.0122
F44	2.0893	2.3243	1.9962

Reaction II

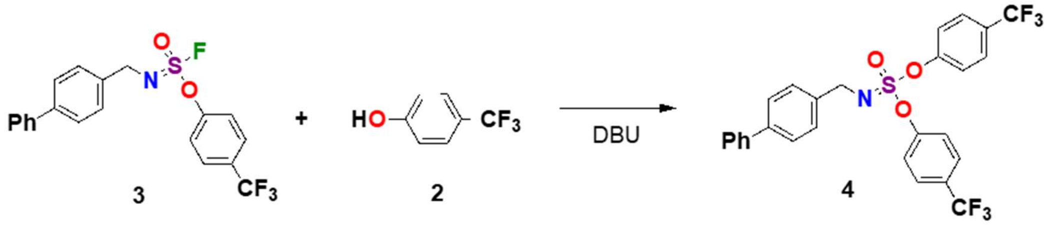

Reactant complex

Thermal correction to Energy: 0.446664

Thermal correction to Enthalpy: 0.447608

Thermal correction to Gibbs Free Energy: 0.338918

Sum of electronic and zero-point Energies: -2417.436126

Sum of electronic and thermal Energies: -2417.401665

Sum of electronic and thermal Enthalpies: -2417.400720

Sum of electronic and thermal Free Energies: -2417.509411

Center Number	X	Y	Z
C1	6.5187	-1.0377	-1.4069
C2	5.2531	-0.5209	-1.6596
C3	4.2106	-0.7006	-0.7443
C4	4.4710	-1.4120	0.4317
C5	5.7365	-1.9292	0.6845
C6	6.7656	-1.7444	-0.2339

H7	7.3122	-0.8936	-2.1325
H8	5.0685	0.0140	-2.5854
H9	3.6811	-1.5480	1.1631
H10	5.9203	-2.4723	1.6055
H11	7.7529	-2.1479	-0.0367
C12	2.8578	-0.1496	-1.0140
C13	1.7095	-0.8718	-0.6843
C14	2.6971	1.1091	-1.6042
C15	0.4431	-0.3523	-0.9259
H16	1.8002	-1.8588	-0.2427
C17	1.4330	1.6278	-1.8408
H18	3.5701	1.7015	-1.8572
C19	0.2904	0.9053	-1.4994
H20	-0.4328	-0.9300	-0.6572
H21	1.3329	2.6145	-2.2822
C22	-1.0643	1.5283	-1.7483
H23	-1.1978	1.6975	-2.8212
H24	-1.1110	2.5000	-1.2476
N25	-2.1121	0.6687	-1.2250
S26	-3.5420	0.5535	-1.5205
O27	-4.5641	1.5617	-1.4955
F28	-3.7492	-0.1182	-3.0211
C29	-1.7677	-2.4562	1.5044
C30	-1.4070	-3.5147	0.6775
C31	-1.9603	-3.6518	-0.5936
C32	-2.8667	-2.7084	-1.0536
C33	-3.1893	-1.6524	-0.2190
C34	-2.6711	-1.5034	1.0527
H35	-1.3340	-2.3539	2.4914
H36	-1.6820	-4.4832	-1.2301
H37	-3.3007	-2.7856	-2.0428
H38	-2.9379	-0.6333	1.6545
O39	-4.0982	-0.6764	-0.6720
C40	-0.4294	-4.5473	1.1568
F41	-1.0452	-5.6808	1.5623
F42	0.3081	-4.1229	2.1960
F43	0.4357	-4.9161	0.1918
C44	-1.1312	3.9771	1.1461
C45	0.1555	3.4265	1.2528
C46	0.2911	2.1306	1.7614
C47	-0.8152	1.4050	2.1551
C48	-2.1484	1.9310	2.0705
C49	-2.2428	3.2608	1.5315
H50	-1.2549	4.9818	0.7507
H51	1.2762	1.6817	1.8404
H52	-0.6881	0.3974	2.5401
H53	-3.2316	3.7001	1.4347
O54	-3.1692	1.2718	2.4392
C55	1.3282	4.2382	0.8661
F56	1.5235	5.3264	1.6722
F57	1.2320	4.7660	-0.3855
F58	2.4905	3.5567	0.8961

TS

Imaginary Frequency: -83.0349 cm^{-1}

Thermal correction to Energy: 0.445777

Thermal correction to Enthalpy: 0.446721

Thermal correction to Gibbs Free Energy: 0.339373

Sum of electronic and zero-point Energies: -2417.415092

Sum of electronic and thermal Energies: -2417.381756

Sum of electronic and thermal Enthalpies: -2417.380811

Sum of electronic and thermal Free Energies: -2417.488160

Center Number	X	Y	Z
C1	-9.7987	0.0155	0.8414
C2	-8.4135	-0.1011	0.8181
C3	-7.6948	0.1386	-0.3580
C4	-8.4022	0.4992	-1.5098
C5	-9.7874	0.6156	-1.4866
C6	-10.4915	0.3739	-0.3109
H7	-10.3384	-0.1816	1.7616
H8	-7.8866	-0.3977	1.7189
H9	-7.8633	0.7064	-2.4284
H10	-10.3171	0.9025	-2.3888
H11	-11.5721	0.4648	-0.2928
C12	-6.2144	0.0114	-0.3831
C13	-5.5604	-0.5475	-1.4828
C14	-5.4372	0.4493	0.6963
C15	-4.1742	-0.6673	-1.5024
H16	-6.1379	-0.9105	-2.3265
C17	-4.0557	0.3295	0.6718
H18	-5.9176	0.9056	1.5555
C19	-3.4044	-0.2308	-0.4283
H20	-3.6873	-1.1135	-2.3642
H21	-3.4710	0.6828	1.5159
C22	-1.8982	-0.3486	-0.4464
H23	-1.4602	0.6266	-0.2236
H24	-1.5642	-0.6439	-1.4432
N25	-1.5129	-1.3550	0.5415
S26	-0.1544	-1.4613	1.2023
O27	0.4694	-0.6371	2.2222
F28	-0.6826	-2.6977	2.2343
C29	3.9722	-2.6746	-1.3026
C30	4.8630	-2.1113	-0.3945
C31	4.4237	-1.7078	0.8642
C32	3.0919	-1.8549	1.2162
C33	2.2016	-2.3837	0.2874
C34	2.6353	-2.8080	-0.9614
H35	4.3098	-2.9966	-2.2798
H36	5.1197	-1.2810	1.5777
H37	2.7434	-1.5434	2.1915
H38	1.9209	-3.2220	-1.6620
O39	0.8828	-2.6282	0.5954
C40	3.3689	2.1065	-0.8591
C41	2.9531	3.1442	-0.0188

C42	1.7876	2.9912	0.7308
C43	1.0393	1.8288	0.6426
C44	1.4381	0.7680	-0.2017
C45	2.6293	0.9447	-0.9485
H46	4.2781	2.2086	-1.4428
H47	1.4543	3.7879	1.3866
H48	0.1371	1.7242	1.2319
H49	2.9525	0.1368	-1.5960
O50	0.7589	-0.3358	-0.3460
C51	3.7921	4.3648	0.1113
F52	3.1284	5.4129	0.6385
F53	4.2937	4.7872	-1.0727
F54	4.8792	4.1801	0.9107
C55	6.3083	-1.9368	-0.7412
F56	6.7232	-0.6615	-0.5737
F57	7.1186	-2.6919	0.0365
F58	6.5884	-2.2664	-2.0131

Product complex

Thermal correction to Energy: 0.447871

Thermal correction to Enthalpy: 0.448816

Thermal correction to Gibbs Free Energy: 0.335922

Sum of electronic and zero-point Energies: -2417.446075

Sum of electronic and thermal Energies: -2417.411388

Sum of electronic and thermal Enthalpies: -2417.410444

Sum of electronic and thermal Free Energies: -2417.523338

Center Number	X	Y	Z
C1	-6.9099	0.9661	-1.2045
C2	-5.8565	0.0756	-1.3765
C3	-4.5525	0.5398	-1.5786
C4	-4.3345	1.9213	-1.6068
C5	-5.3875	2.8122	-1.4355
C6	-6.6797	2.3381	-1.2317
H7	-7.9144	0.5859	-1.0526
H8	-6.0525	-0.9913	-1.3637
H9	-3.3283	2.3039	-1.7408
H10	-5.1954	3.8797	-1.4508
H11	-7.5012	3.0329	-1.0943
C12	-3.4172	-0.4066	-1.7224
C13	-2.3437	-0.1281	-2.5703
C14	-3.3739	-1.5823	-0.9647

C15	-1.2472	-0.9811	-2.6352
H16	-2.3551	0.7680	-3.1817
C17	-2.2844	-2.4353	-1.0384
H18	-4.1853	-1.8152	-0.2838
C19	-1.2007	-2.1385	-1.8649
H20	-0.4133	-0.7341	-3.2852
H21	-2.2596	-3.3278	-0.4212
C22	0.0091	-3.0426	-1.8880
H23	0.7413	-2.6753	-2.6121
H24	-0.2814	-4.0488	-2.1947
N25	0.6211	-3.2141	-0.5563
S26	1.4989	-2.1852	0.0768
O27	2.0759	-2.5084	1.3563
F28	7.6850	-2.5998	1.9254
C29	4.1432	1.5730	-1.2152
C30	5.2177	1.4315	-0.3382
C31	5.4371	0.2356	0.3340
C32	4.5703	-0.8331	0.1398
C33	3.5015	-0.6674	-0.7221
C34	3.2718	0.5146	-1.4090
H35	3.9812	2.5057	-1.7428
H36	6.2779	0.1225	1.0063
H37	4.7288	-1.7752	0.6489
H38	2.4245	0.5989	-2.0777
O39	2.6423	-1.7430	-0.9769
C40	6.1570	2.5885	-0.1603
F41	5.4994	3.7503	0.0314
F42	6.9388	2.7771	-1.2470
F43	6.9849	2.4358	0.8841
C44	-2.0449	-0.8362	2.4340
C45	-2.6811	0.3397	2.0469
C46	-2.1154	1.1593	1.0777
C47	-0.9252	0.7897	0.4736
C48	-0.3131	-0.3953	0.8526
C49	-0.8454	-1.2076	1.8462
H50	-2.4812	-1.4739	3.1941
H51	-2.6052	2.0753	0.7743
H52	-0.4790	1.4005	-0.3008
H53	-0.3566	-2.1222	2.1562
O54	0.8669	-0.6704	0.1792
C55	-4.0048	0.6842	2.6574
F56	-4.4440	1.9023	2.3026
F57	-3.9700	0.6627	4.0072
F58	-4.9715	-0.1911	2.2985

Reaction IIIa

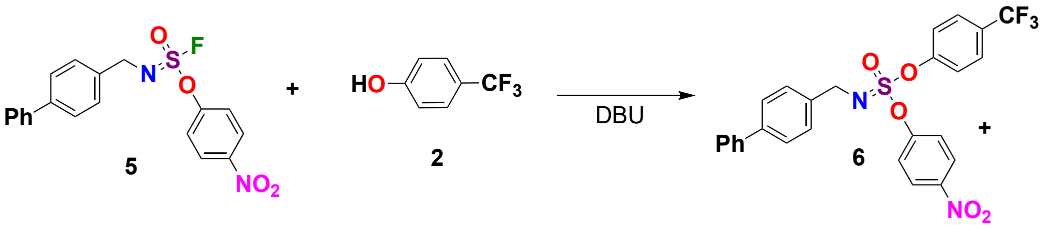

Reactant complex

Thermal correction to Energy: 0.443880

Thermal correction to Enthalpy: 0.444824

Thermal correction to Gibbs Free Energy: 0.340877

Sum of electronic and zero-point Energies: -2284.871587

Sum of electronic and thermal Energies: -2284.838401

Sum of electronic and thermal Enthalpies: -2284.837457

Sum of electronic and thermal Free Energies: -2284.941405

Center Number	X	Y	Z
C1	-6.3185	1.0728	-1.1937
C2	-5.0967	0.4529	-0.9600
C3	-5.0365	-0.8312	-0.4094
C4	-6.2378	-1.4793	-0.1015
C5	-7.4606	-0.8602	-0.3352
C6	-7.5065	0.4189	-0.8818
H7	-6.3403	2.0732	-1.6126
H8	-4.1781	0.9786	-1.1924
H9	-6.2187	-2.4832	0.3093
H10	-8.3807	-1.3825	-0.0951
H11	-8.4606	0.9020	-1.0629
C12	-3.7260	-1.4841	-0.1580
C13	-3.5274	-2.2975	0.9633
C14	-2.6503	-1.2937	-1.0268
C15	-2.2982	-2.8938	1.2007
H16	-4.3355	-2.4489	1.6712
C17	-1.4174	-1.8889	-0.7847
H18	-2.7724	-0.6772	-1.9111
C19	-1.2256	-2.6934	0.3314
H20	-2.1642	-3.5085	2.0862
H21	-0.5942	-1.7164	-1.4681
C22	0.1029	-3.3259	0.6692
H23	-0.0233	-4.4078	0.7657
H24	0.4362	-2.9296	1.6313
N25	1.1020	-3.0811	-0.3740
S26	2.4606	-2.5703	-0.1039
O27	3.1892	-2.5824	1.1330
F28	3.4044	-3.4078	-1.1405
C29	4.9008	1.1991	0.7499
C30	5.8956	1.2599	-0.2178
C31	5.8496	0.5173	-1.3895
C32	4.7683	-0.3247	-1.5989
C33	3.7800	-0.3828	-0.6301
C34	3.8174	0.3626	0.5387
H35	4.9693	1.7994	1.6469
H36	6.6420	0.5930	-2.1216
H37	4.6917	-0.9237	-2.4974
H38	2.9940	0.2696	1.2424
O39	2.6568	-1.1745	-0.8837
C40	-0.6712	2.0920	-0.2836
C41	-1.8579	2.2636	0.4442

C42	-2.0663	1.4769	1.5839
C43	-1.1239	0.5542	1.9884
C44	0.1067	0.3517	1.2791
C45	0.2745	1.1700	0.1089
H46	-0.4933	2.6925	-1.1715
H47	-2.9812	1.5891	2.1570
H48	-1.3080	-0.0564	2.8677
H49	1.1765	1.0391	-0.4792
O50	0.9832	-0.4878	1.6548
C51	-2.8114	3.3215	0.0497
F52	-4.0048	3.2322	0.6716
F53	-3.0776	3.3401	-1.2839
F54	-2.3611	4.5868	0.3183
N55	7.0440	2.1478	0.0071
O56	7.1128	2.7387	1.0694
O57	7.8729	2.2495	-0.8791

TS

Imaginary Frequency: -82.9495 cm^{-1}

Thermal correction to Energy: 0.442976

Thermal correction to Enthalpy: 0.443920

Thermal correction to Gibbs Free Energy: 0.338661

Sum of electronic and zero-point Energies: -2284.857346

Sum of electronic and thermal Energies: -2284.825059

Sum of electronic and thermal Enthalpies: -2284.824115

Sum of electronic and thermal Free Energies: -2284.929374

Center Number	X	Y	Z
C1	-9.5474	0.1553	0.8355
C2	-8.1637	0.0212	0.8217
C3	-7.4339	0.2510	-0.3495
C4	-8.1286	0.6199	-1.5064
C5	-9.5124	0.7539	-1.4928
C6	-10.2276	0.5219	-0.3218
H7	-10.0958	-0.0343	1.7520
H8	-7.6467	-0.2814	1.7264
H9	-7.5809	0.8196	-2.4214
H10	-10.0323	1.0469	-2.3987
H11	-11.3071	0.6265	-0.3112
C12	-5.9552	0.1041	-0.3645
C13	-5.3016	-0.4650	-1.4593
C14	-5.1795	0.5326	0.7196
C15	-3.9173	-0.6044	-1.4692

H16	-5.8784	-0.8208	-2.3066
C17	-3.7996	0.3934	0.7047
H18	-5.6594	0.9966	1.5750
C19	-3.1491	-0.1778	-0.3900
H20	-3.4308	-1.0587	-2.3270
H21	-3.2158	0.7395	1.5525
C22	-1.6452	-0.3217	-0.3964
H23	-1.1909	0.6434	-0.1617
H24	-1.3068	-0.6175	-1.3912
N25	-1.2882	-1.3416	0.5889
S26	0.0721	-1.5109	1.2273
O27	0.7543	-0.7217	2.2367
F28	-0.4804	-2.7324	2.2538
C29	4.0741	-2.9122	-1.3963
C30	4.9950	-2.3602	-0.5124
C31	4.6237	-1.9052	0.7459
C32	3.2980	-1.9994	1.1318
C33	2.3645	-2.5187	0.2389
C34	2.7484	-2.9886	-1.0147
H35	4.3886	-3.2623	-2.3701
H36	5.3618	-1.4911	1.4196
H37	2.9875	-1.6540	2.1080
H38	2.0001	-3.3928	-1.6844
O39	1.0531	-2.7153	0.5835
C40	3.6600	1.9781	-0.9023
C41	3.2790	3.0235	-0.0553
C42	2.1261	2.8912	0.7174
C43	1.3554	1.7427	0.6450
C44	1.7180	0.6749	-0.2069
C45	2.8983	0.8295	-0.9760
H46	4.5590	2.0639	-1.5041
H47	1.8205	3.6941	1.3793
H48	0.4633	1.6552	1.2523
H49	3.1932	0.0157	-1.6300
O50	1.0177	-0.4167	-0.3379
C51	4.1411	4.2293	0.0599
F52	3.4964	5.2965	0.5727
F53	4.6521	4.6263	-1.1284
F54	5.2231	4.0350	0.8642
N55	6.3931	-2.2534	-0.9208
O56	6.7139	-2.7065	-2.0070
O57	7.1783	-1.7140	-0.1592

Product complex

Thermal correction to Energy: 0.444776

Thermal correction to Enthalpy: 0.445721

Thermal correction to Gibbs Free Energy: 0.337810

Sum of electronic and zero-point Energies: -2284.879405

Sum of electronic and thermal Energies: -2284.846075

Sum of electronic and thermal Enthalpies: -2284.845131

Sum of electronic and thermal Free Energies: -2284.953042

Center Number	X	Y	Z
C1	-7.9788	0.4531	-0.9240
C2	-6.6379	0.5858	-0.5816
C3	-6.0984	1.8395	-0.2742
C4	-6.9395	2.9565	-0.3188
C5	-8.2805	2.8239	-0.6615
C6	-8.8057	1.5716	-0.9653
H7	-8.3765	-0.5269	-1.1650
H8	-6.0000	-0.2916	-0.5684
H9	-6.5465	3.9360	-0.0673
H10	-8.9181	3.7012	-0.6834
H11	-9.8519	1.4680	-1.2318
C12	-4.6649	1.9790	0.0919
C13	-3.9120	3.0698	-0.3530
C14	-4.0334	1.0223	0.8931
C15	-2.5713	3.1933	-0.0107
H16	-4.3722	3.8216	-0.9857
C17	-2.6931	1.1465	1.2363
H18	-4.5994	0.1767	1.2703
C19	-1.9462	2.2340	0.7838
H20	-2.0015	4.0438	-0.3730
H21	-2.2421	0.3813	1.8670
C22	-0.4766	2.3498	1.1092
H23	-0.2764	1.9541	2.1094
H24	-0.1726	3.3969	1.0956
N25	0.3540	1.6797	0.0865
S26	0.7877	0.2588	0.2620
O27	1.1543	-0.3274	1.5327
F28	-2.1866	-1.5236	3.0485
C29	0.6201	-4.1547	-1.3639
C30	0.0475	-4.8256	-0.2916
C31	-0.6134	-4.1663	0.7360
C32	-0.7090	-2.7829	0.6972
C33	-0.1328	-2.1178	-0.3745
C34	0.5257	-2.7734	-1.4051
H35	1.1238	-4.6988	-2.1511
H36	-1.0489	-4.7228	1.5550
H37	-1.2262	-2.2389	1.4936
H38	0.9522	-2.2107	-2.2255
O39	-0.2811	-0.7326	-0.4626
C40	4.7167	2.4090	-0.9104
C41	5.6866	1.6904	-0.2174
C42	5.4159	0.4197	0.2814
C43	4.1589	-0.1373	0.0967
C44	3.2037	0.5971	-0.5859
C45	3.4570	1.8588	-1.0977
H46	4.9351	3.3938	-1.3046
H47	6.1770	-0.1386	0.8123
H48	3.9226	-1.1250	0.4725
H49	2.6835	2.3953	-1.6321
O50	1.9564	0.0104	-0.8263
C51	7.0645	2.2648	-0.0571
F52	7.6633	1.8563	1.0755
F53	7.0654	3.6089	-0.0355
F54	7.8829	1.9011	-1.0706
N55	0.1427	-6.2902	-0.2465
O56	0.7718	-6.8521	-1.1254

O57	-0.4119	-6.8742	0.6667

Reaction IIIb

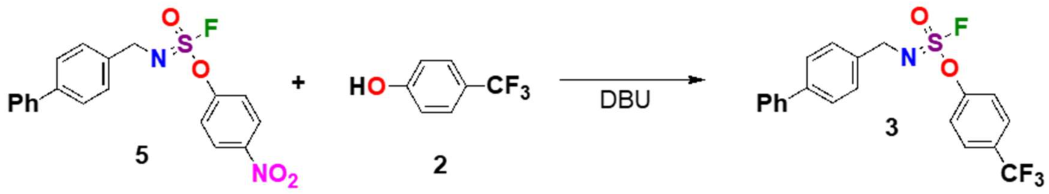

Reactant complex

Thermal correction to Energy: 0.443763

Thermal correction to Enthalpy: 0.444707

Thermal correction to Gibbs Free Energy: 0.333330

Sum of electronic and zero-point Energies: -2284.865778

Sum of electronic and thermal Energies: -2284.831954

Sum of electronic and thermal Enthalpies: -2284.831010

Sum of electronic and thermal Free Energies: -2284.942387

Center Number	X	Y	Z
C1	-9.4060	-0.3195	0.8064
C2	-8.0707	0.0649	0.7628
C3	-7.1610	-0.6007	-0.0657
C4	-7.6261	-1.6622	-0.8493
C5	-8.9614	-2.0468	-0.8057
C6	-9.8570	-1.3771	0.0225
H7	-10.0943	0.2049	1.4606
H8	-7.7281	0.8799	1.3917
H9	-6.9413	-2.1818	-1.5113
H10	-9.3038	-2.8683	-1.4260
H11	-10.8986	-1.6772	0.0567
C12	-5.7334	-0.1905	-0.1124
C13	-4.7144	-1.1441	-0.1959
C14	-5.3702	1.1585	-0.0687
C15	-3.3795	-0.7625	-0.2370
H16	-4.9650	-2.1996	-0.2118
C17	-4.0347	1.5382	-0.1056
H18	-6.1381	1.9230	-0.0132
C19	-3.0228	0.5835	-0.1931
H20	-2.6086	-1.5239	-0.2913
H21	-3.7761	2.5920	-0.0685
C22	-1.5861	1.0360	-0.2904

H23	-1.4674	2.0127	0.1881
H24	-1.3129	1.1434	-1.3459
N25	-0.7028	0.0747	0.3888
S26	0.5942	-0.3643	-0.1683
O27	1.0279	-0.2805	-1.5376
F28	1.8311	0.3097	0.6674
C29	3.5820	-3.7922	-0.9066
C30	4.3861	-3.8203	0.2236
C31	4.0353	-3.1916	1.4103
C32	2.8347	-2.5033	1.4655
C33	2.0356	-2.4744	0.3346
C34	2.3777	-3.1083	-0.8484
H35	3.8840	-4.2992	-1.8129
H36	4.6863	-3.2360	2.2726
H37	2.5219	-1.9954	2.3688
H38	1.7169	-3.0747	-1.7049
O39	0.7913	-1.8356	0.4337
C40	2.9389	3.5850	1.2267
C41	3.2224	3.3698	-0.1299
C42	2.1612	3.3488	-1.0428
C43	0.8589	3.5165	-0.6191
C44	0.5206	3.7229	0.7626
C45	1.6423	3.7522	1.6620
H46	3.7507	3.6130	1.9480
H47	2.3604	3.1909	-2.0982
H48	0.0491	3.4899	-1.3422
H49	1.4413	3.9062	2.7185
O50	-0.6766	3.8638	1.1586
C51	4.6218	3.2354	-0.5842
F52	4.7410	2.5976	-1.7702
F53	5.4051	2.5573	0.2911
F54	5.2632	4.4327	-0.7614
N55	5.6619	-4.5490	0.1632
O56	5.9872	-5.0464	-0.8987
O57	6.3293	-4.6183	1.1784

TS

Imaginary Frequency: -29.6014 cm^{-1}

Thermal correction to Energy: 0.442782

Thermal correction to Enthalpy: 0.443726

Thermal correction to Gibbs Free Energy: 0.337939

Sum of electronic and zero-point Energies: -2284.846718

Sum of electronic and thermal Energies: -2284.814358

Sum of electronic and thermal Enthalpies: -2284.813413

Sum of electronic and thermal Free Energies: -2284.919201

Center Number	X	Y	Z
C1	-9.5072	0.4780	-0.4490
C2	-8.1505	0.2827	-0.6812
C3	-7.3552	-0.3952	0.2490
C4	-7.9569	-0.8702	1.4193
C5	-9.3136	-0.6745	1.6519
C6	-10.0946	0.0003	0.7185
H7	-10.1052	1.0118	-1.1799
H8	-7.7012	0.6751	-1.5874
H9	-7.3626	-1.4122	2.1475
H10	-9.7625	-1.0567	2.5624
H11	-11.1529	0.1530	0.8998
C12	-5.9056	-0.6071	-0.0006
C13	-4.9749	-0.5038	1.0401
C14	-5.4346	-0.9178	-1.2780
C15	-3.6223	-0.7045	0.8063
H16	-5.3114	-0.2503	2.0400
C17	-4.0770	-1.1171	-1.5080
H18	-6.1344	-1.0213	-2.1008
C19	-3.1537	-1.0150	-0.4713
H20	-2.9167	-0.6134	1.6267
H21	-3.7342	-1.3632	-2.5085
C22	-1.6778	-1.2316	-0.7124
H23	-1.5295	-1.6685	-1.7023
H24	-1.2889	-1.9390	0.0234
N25	-1.0221	0.0736	-0.6133
S26	0.4760	0.2746	-0.4147
O27	1.3351	0.1724	0.7623
F28	1.4510	0.6308	-1.7678
C29	2.7522	3.9683	1.3752
C30	3.2239	4.7182	0.3040
C31	2.7208	4.5621	-0.9824
C32	1.7270	3.6244	-1.1976
C33	1.2425	2.8569	-0.1360
C34	1.7539	3.0364	1.1519
H35	3.1551	4.1180	2.3680
H36	3.1023	5.1621	-1.7977
H37	1.3152	3.4753	-2.1878
H38	1.3640	2.4441	1.9681
O39	0.2048	2.0143	-0.3667
C40	4.4101	-2.4586	-0.9248
C41	4.4004	-3.4252	0.0767
C42	3.2075	-3.7355	0.7328
C43	2.0393	-3.0802	0.3887
C44	2.0306	-2.1065	-0.6244
C45	3.2370	-1.8054	-1.2734
H46	5.3315	-2.2133	-1.4397
H47	3.1945	-4.4855	1.5170
H48	1.1094	-3.3098	0.8970
H49	3.2428	-1.0558	-2.0547
O50	0.8784	-1.5367	-0.9717
C51	5.6512	-4.1314	0.4828
F52	6.7127	-3.8129	-0.2786
F53	5.5298	-5.4792	0.4224
F54	6.0082	-3.8600	1.7625
N55	4.2764	5.7045	0.5379
O56	4.7132	5.8270	1.6702
O57	4.6732	6.3624	-0.4095

Product complex

Thermal correction to Energy: 0.444144

Thermal correction to Enthalpy: 0.445088

Thermal correction to Gibbs Free Energy: 0.338864

Sum of electronic and zero-point Energies: -2284.882953

Sum of electronic and thermal Energies: -2284.849530

Sum of electronic and thermal Enthalpies: -2284.848586

Sum of electronic and thermal Free Energies: -2284.954810

Center Number	X	Y	Z
C1	-7.1382	1.7637	-1.3012
C2	-5.9070	1.1798	-1.0243
C3	-5.6925	-0.1812	-1.2673
C4	-6.7435	-0.9414	-1.7907
C5	-7.9745	-0.3568	-2.0667
C6	-8.1765	0.9986	-1.8238
H7	-7.2882	2.8192	-1.0998
H8	-5.1130	1.7834	-0.5953
H9	-6.5929	-1.9958	-1.9982
H10	-8.7757	-0.9611	-2.4786
H11	-9.1368	1.4548	-2.0389
C12	-4.3774	-0.8003	-0.9630
C13	-4.2958	-2.0693	-0.3808
C14	-3.1876	-0.1194	-1.2262
C15	-3.0666	-2.6254	-0.0583
H16	-5.2037	-2.6188	-0.1551
C17	-1.9570	-0.6778	-0.9035
H18	-3.2187	0.8613	-1.6896
C19	-1.8821	-1.9304	-0.3045
H20	-3.0273	-3.6048	0.4090
H21	-1.0504	-0.1221	-1.1139
C22	-0.5757	-2.5342	0.1516
H23	-0.5926	-3.6123	-0.0218
H24	-0.4701	-2.3789	1.2315
N25	0.5664	-1.9966	-0.5988
S26	1.7318	-1.3499	0.0442
O27	2.1794	-1.5178	1.4013
F28	1.6529	0.2855	-0.1198
C29	-1.0080	1.4003	1.8335
C30	-2.3324	1.8085	1.5835
C31	-3.4073	1.0016	2.0095
C32	-3.1682	-0.1784	2.6559
C33	-1.8274	-0.6568	2.9140
C34	-0.7617	0.2198	2.4786
H35	-0.1893	2.0281	1.5038
H36	-4.4223	1.3221	1.8077
H37	-3.9966	-0.8052	2.9707
H38	0.2586	-0.0948	2.6719

O39	-1.6018	-1.7597	3.4688
C40	6.3013	-0.7977	0.1436
C41	6.5722	0.3263	-0.6302
C42	5.6197	0.8400	-1.5055
C43	4.3829	0.2220	-1.6141
C44	4.1355	-0.8945	-0.8357
C45	5.0681	-1.4250	0.0377
H46	7.0460	-1.1954	0.8218
H47	5.8355	1.7148	-2.1062
H48	3.6245	0.5970	-2.2897
H49	4.8414	-2.3104	0.6178
O50	2.9071	-1.5590	-1.0138
C51	7.8940	1.0238	-0.4818
F52	8.2750	1.6504	-1.6083
F53	8.8865	0.1796	-0.1505
F54	7.8589	1.9674	0.4851
N55	-2.5814	2.9963	0.8660
O56	-1.6285	3.6748	0.4656
O57	-3.7511	3.3364	0.6450

Reaction IV

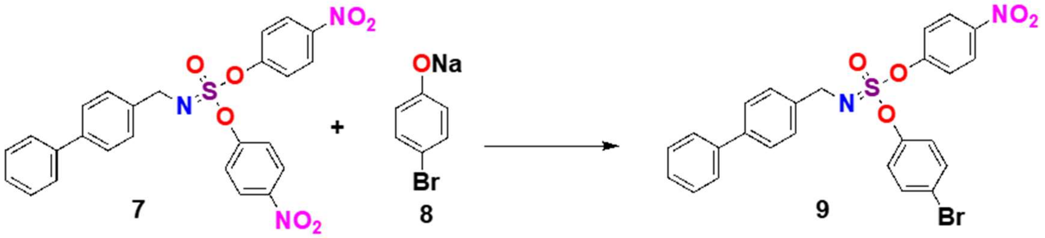

Reactant complex

Thermal correction to Energy: 0.530630

Thermal correction to Enthalpy: 0.531574

Thermal correction to Gibbs Free Energy: 0.413479

Sum of electronic and zero-point Energies: -4932.806972

Sum of electronic and thermal Energies: -4932.768421

Sum of electronic and thermal Enthalpies: -4932.767477

Sum of electronic and thermal Free Energies: -4932.885573

Center Number	X	Y	Z
C1	-4.9794	6.9992	0.4581
C2	-3.7124	6.4729	0.6817
C3	-2.7702	6.4126	-0.3496
C4	-3.1314	6.8973	-1.6116
C5	-4.3982	7.4228	-1.8357
C6	-5.3283	7.4750	-0.8016
H7	-5.6990	7.0278	1.2693
H8	-3.4614	6.0866	1.6635
H9	-2.4113	6.8778	-2.4227
H10	-4.6573	7.7980	-2.8201
H11	-6.3176	7.8838	-0.9766
C12	-1.4200	5.8403	-0.1122
C13	-0.7841	5.0755	-1.0950
C14	-0.7544	6.0454	1.0995
C15	0.4761	4.5386	-0.8744
H16	-1.2870	4.8813	-2.0364
C17	0.5058	5.5038	1.3188
H18	-1.2160	6.6488	1.8739
C19	1.1363	4.7451	0.3356
H20	0.9498	3.9444	-1.6494
H21	1.0103	5.6813	2.2639
C22	2.4980	4.1474	0.5789
H23	3.0408	4.7475	1.3088
H24	3.0831	4.1397	-0.3428
N25	2.4324	2.7911	1.1449

S26	2.4956	1.4860	0.4544
O27	3.4632	0.4707	0.7788
C28	-1.9294	2.5735	1.3524
C29	-2.5722	2.4203	0.1331
C30	-2.0120	1.7270	-0.9292
C31	-0.7652	1.1487	-0.7564
C32	-0.1200	1.2944	0.4638
C33	-0.6774	2.0040	1.5178
H34	-2.3947	3.1295	2.1549
H35	-2.5415	1.6316	-1.8676
H36	-0.3028	0.5844	-1.5563
H37	-0.1408	2.1033	2.4522
O38	1.0936	0.6449	0.6498
C39	4.0642	0.3764	-4.1059
C40	3.6271	-0.9401	-4.0466
C41	2.8170	-1.4059	-3.0207
C42	2.4262	-0.5259	-2.0253
C43	2.8694	0.7898	-2.0851
C44	3.6838	1.2574	-3.1065
H45	4.7011	0.7092	-4.9147
H46	2.4898	-2.4364	-3.0006
H47	1.7870	-0.8674	-1.2222
H48	4.0394	2.2893	-3.1136
O49	2.4659	1.7433	-1.1556
N50	-3.9023	3.0155	-0.0373
O51	-4.5409	3.2781	0.9648
O52	-4.3020	3.2079	-1.1709
N53	4.0354	-1.8724	-5.1021
O54	3.6556	-3.0276	-5.0273
O55	4.7351	-1.4485	-6.0044
C56	5.6266	5.8150	0.0502
C57	5.9088	4.7500	0.9008
C58	5.9530	3.4511	0.4045
C59	5.7121	3.2152	-0.9397
C60	5.3971	4.2656	-1.8626
C61	5.3783	5.5799	-1.2928
H62	5.5917	6.8294	0.4347
H63	6.1721	2.6187	1.0655
H64	5.7514	2.1962	-1.3132
H65	5.1498	6.4167	-1.9472
O66	5.1466	4.0463	-3.0957
Br67	6.2164	5.0712	2.7662

TS

Imaginary Frequency: -142.1850 cm^{-1}

Thermal correction to Energy: 0.529498

Thermal correction to Enthalpy: 0.530442

Thermal correction to Gibbs Free Energy: 0.414076

Sum of electronic and zero-point Energies: -4932.783383

Sum of electronic and thermal Energies: -4932.745824

Sum of electronic and thermal Enthalpies: -4932.744880

Sum of electronic and thermal Free Energies: -4932.861246

Center Number	X	Y	Z
C1	-5.7097	6.3979	0.1452
C2	-4.3711	6.1260	0.4021
C3	-3.4413	6.0576	-0.6400
C4	-3.8910	6.2702	-1.9480
C5	-5.2297	6.5410	-2.2056
C6	-6.1453	6.6056	-1.1596
H7	-6.4162	6.4367	0.9675
H8	-4.0503	5.9425	1.4215
H9	-3.1856	6.2385	-2.7717
H10	-5.5567	6.7074	-3.2265
H11	-7.1904	6.8152	-1.3601
C12	-2.0120	5.7584	-0.3656
C13	-1.2662	4.9610	-1.2415
C14	-1.3763	6.2553	0.7740
C15	0.0646	4.6725	-0.9833
H16	-1.7394	4.5413	-2.1231
C17	-0.0414	5.9595	1.0321
H18	-1.9227	6.8886	1.4650
C19	0.6961	5.1643	0.1602
H20	0.6157	4.0327	-1.6646
H21	0.4302	6.3532	1.9275
C22	2.1261	4.7838	0.4678
H23	2.5084	5.4222	1.2689
H24	2.7587	4.9317	-0.4106
N25	2.1926	3.4034	0.9472
S26	2.7800	2.2383	0.1861
O27	3.7470	1.2653	0.6692
C28	-1.8108	2.5527	1.5725
C29	-2.5443	2.1637	0.4565
C30	-1.9783	1.4030	-0.5624
C31	-0.6546	1.0229	-0.4540
C32	0.1188	1.4076	0.6559
C33	-0.4837	2.1784	1.6654
H34	-2.2752	3.1484	2.3477
H35	-2.5727	1.1103	-1.4184
H36	-0.1937	0.4217	-1.2282
H37	0.1114	2.4797	2.5172
O38	1.3855	1.0194	0.7580
C39	4.1038	-0.3274	-3.3498
C40	3.2365	-0.4471	-4.4275
C41	2.0239	0.2341	-4.4661
C42	1.6799	1.0419	-3.4019
C43	2.5477	1.1715	-2.3120
C44	3.7637	0.4873	-2.2836
H45	5.0428	-0.8646	-3.3417
H46	1.3599	0.1301	-5.3138
H47	0.7419	1.5832	-3.3984
H48	4.4363	0.5862	-1.4453
O49	2.0916	2.0061	-1.3463
N50	-3.9391	2.5605	0.3550
O51	-4.4994	2.9714	1.3591
O52	-4.4946	2.4594	-0.7287
N53	3.6040	-1.3083	-5.5445

O54	2.8224	-1.4127	-6.4759
O55	4.6768	-1.8881	-5.5008
C56	7.6094	3.8208	-0.8382
C57	7.7966	4.2429	0.4722
C58	6.7327	4.2939	1.3616
C59	5.4617	3.9256	0.9372
C60	5.2473	3.4945	-0.3790
C61	6.3412	3.4415	-1.2540
H62	8.4403	3.7780	-1.5326
H63	6.8805	4.6232	2.3835
H64	4.6357	3.9730	1.6360
H65	6.1804	3.1025	-2.2715
O66	4.0490	3.1647	-0.8759
Br67	9.5414	4.7591	1.0577

Product complex

Thermal correction to Energy: 0.530611

Thermal correction to Enthalpy: 0.531555

Thermal correction to Gibbs Free Energy: 0.412499

Sum of electronic and zero-point Energies: -4932.821654

Sum of electronic and thermal Energies: -4932.783038

Sum of electronic and thermal Enthalpies: -4932.782094

Sum of electronic and thermal Free Energies: -4932.901149

Center Number	X	Y	Z
C1	-1.2899	10.2380	0.7471
C2	-1.3358	8.8872	0.4230
C3	-1.2395	7.9087	1.4174
C4	-1.1017	8.3201	2.7468
C5	-1.0531	9.6708	3.0715
C6	-1.1450	10.6356	2.0727
H7	-1.3571	10.9817	-0.0398
H8	-1.4237	8.5904	-0.6168
H9	-1.0413	7.5785	3.5364
H10	-0.9486	9.9701	4.1089
H11	-1.1055	11.6895	2.3259
C12	-1.2556	6.4673	1.0615
C13	-0.4326	5.5563	1.7291
C14	-2.0735	5.9909	0.0322
C15	-0.4185	4.2151	1.3692
H16	0.2255	5.9016	2.5192
C17	-2.0592	4.6495	-0.3237
H18	-2.7373	6.6730	-0.4884
C19	-1.2270	3.7470	0.3358
H20	0.2415	3.5272	1.8890

H21	-2.7025	4.2977	-1.1247
C22	-1.1589	2.3070	-0.1045
H23	-2.1460	1.9561	-0.4093
H24	-0.8186	1.6737	0.7188
N25	-0.2908	2.1623	-1.2900
S26	1.1385	1.7479	-1.1598
O27	1.7401	1.2900	0.0741
C28	-1.9843	-0.9756	0.0269
C29	-2.2054	-0.9573	-1.3640
C30	-1.3028	-1.6100	-2.2260
C31	-0.2091	-2.2532	-1.7169
C32	0.0816	-2.2736	-0.3010
C33	-0.8888	-1.6122	0.5420
H34	-2.6866	-0.4726	0.6804
H35	-1.4814	-1.5884	-3.2942
H36	0.4931	-2.7449	-2.3826
H37	-0.7134	-1.6157	1.6132
O38	1.1155	-2.8181	0.1627
C39	4.6211	0.1034	-3.9080
C40	5.0623	-0.9593	-3.1309
C41	4.3141	-1.4837	-2.0870
C42	3.0789	-0.9222	-1.8016
C43	2.6450	0.1463	-2.5716
C44	3.3881	0.6646	-3.6219
H45	5.2271	0.4829	-4.7192
H46	4.6853	-2.3190	-1.5087
H47	2.4638	-1.3268	-1.0048
H48	3.0045	1.4936	-4.2022
O49	1.3757	0.6794	-2.3435
N50	-3.3107	-0.2557	-1.8943
O51	-4.0794	0.3318	-1.1237
O52	-3.4847	-0.2363	-3.1174
N53	6.3727	-1.5527	-3.4273
O54	6.7214	-2.5245	-2.7823
O55	7.0481	-1.0427	-4.3028
C56	1.5119	6.3907	-1.0608
C57	2.4278	6.5761	-0.0330
C58	3.2262	5.5382	0.4294
C59	3.1126	4.2846	-0.1579
C60	2.2035	4.1048	-1.1879
C61	1.3978	5.1358	-1.6453
H62	0.8896	7.2070	-1.4052
H63	3.9359	5.6961	1.2317
H64	3.7299	3.4595	0.1758
H65	0.6939	4.9607	-2.4495
O66	2.1377	2.8661	-1.8283
Br67	2.6041	8.2993	0.7590

Reaction I with DBU-H⁺

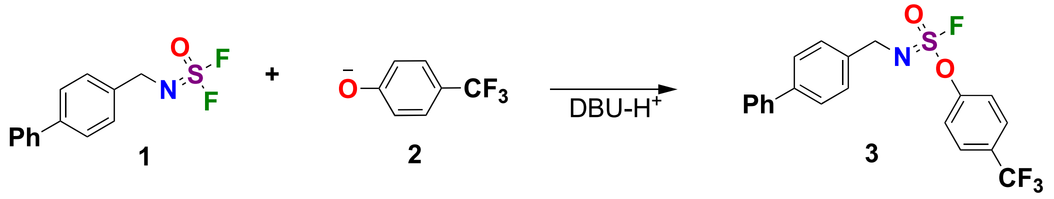

Reactant complex

Thermal correction to Energy: 0.615742

Thermal correction to Enthalpy: 0.616686

Thermal correction to Gibbs Free Energy: 0.503843

Sum of electronic and zero-point Energies: -2335.806169

Sum of electronic and thermal Energies: -2335.768635

Sum of electronic and thermal Enthalpies: -2335.767691

Sum of electronic and thermal Free Energies: -2335.880534

Center Number	X	Y	Z
C1	-1.300859	-3.504153	0.365426
C2	-2.119009	-2.946079	-0.626446
C3	-1.580137	-2.740199	-1.903785
C4	-0.267735	-3.059165	-2.179373
C5	0.613492	-3.61957	-1.188848
C6	0.011541	-3.83189	0.100271
H7	-1.701308	-3.673088	1.360651
H8	-2.199714	-2.311428	-2.686081
H9	0.136076	-2.878951	-3.171924
H10	0.633133	-4.25225	0.885637
N11	1.310765	2.929816	-0.140813
N12	2.866744	2.040472	1.304164
C13	0.75945	3.102159	-1.492604
C14	1.556779	4.075162	-2.355834
C15	2.429713	2.267546	0.075425
C16	2.812297	3.455185	-2.966081

C17	0.526569	3.488671	0.969097
C18	3.264477	1.770347	-1.065719
C19	3.813597	2.898651	-1.955359
C20	1.399108	3.744826	2.183494
C21	2.19102	2.49383	2.513524
H22	-0.259548	3.461065	-1.352401
H23	0.687132	2.124733	-1.976268
H24	1.809608	4.957066	-1.756493
H25	0.90203	4.414507	-3.163828
H26	3.317168	4.203082	-3.584922
H27	2.508002	2.643342	-3.638147
H28	-0.281724	2.790374	1.204392
H29	0.082922	4.419548	0.614239
H30	4.093829	1.201229	-0.644524
H31	2.665103	1.071254	-1.657804
H32	4.200257	3.702654	-1.319567
H33	4.668916	2.490859	-2.49995
H34	0.767987	4.021933	3.028362
H35	2.083754	4.573276	1.98166
H36	2.952507	2.685658	3.268815
H37	1.530281	1.702135	2.878194
O38	1.82373	-3.900431	-1.431889
H39	3.744746	1.552429	1.397787
C40	-5.820755	1.880405	0.780108
C41	-4.625504	1.228484	1.060408
C42	-3.448292	1.573938	0.388897
C43	-3.500679	2.590716	-0.570333
C44	-4.695998	3.24284	-0.851002
C45	-5.861041	2.890176	-0.17674
H46	-6.724958	1.593134	1.306099
H47	-4.610641	0.431294	1.79573
H48	-2.5947	2.88491	-1.090579
H49	-4.715468	4.033148	-1.593902
H50	-6.793902	3.398382	-0.395547
C51	-2.168658	0.882577	0.685655
C52	-1.262508	0.5872	-0.331998
C53	-1.827628	0.527408	1.995745
C54	-0.0492	-0.031714	-0.056243
H55	-1.508191	0.830021	-1.359959
C56	-0.618262	-0.091269	2.270454
H57	-2.50479	0.756492	2.811635
C58	0.284576	-0.377683	1.245314

H59	0.635266	-0.25365	-0.866285
H60	-0.369305	-0.352632	3.294705
C61	1.570699	-1.080256	1.60004
H62	1.340151	-2.076617	1.987863
H63	2.090743	-0.524258	2.385975
N64	2.431746	-1.194048	0.422267
S65	3.705525	-1.910225	0.335561
O66	4.46527	-1.985662	-0.865572
F67	4.749334	-1.382573	1.470937
F68	3.675973	-3.368051	1.002617
C69	-3.535401	-2.633932	-0.348956
F70	-4.393855	-3.669176	-0.621246
F71	-4.010336	-1.598243	-1.079888
F72	-3.772279	-2.327095	0.947646

TS

Imaginary Frequency: -178.6866 cm^{-1}

Thermal correction to Energy: 0.614532

Thermal correction to Enthalpy: 0.615476

Thermal correction to Gibbs Free Energy: 0.504627

Sum of electronic and zero-point Energies: -2335.79955

Sum of electronic and thermal Energies: -2335.762894

Sum of electronic and thermal Enthalpies: -2335.76195

Sum of electronic and thermal Free Energies: -2335.872799

Center Number	X	Y	Z
C1	-0.499028	-3.328514	-0.376193
C2	0.219185	-3.183125	0.812561
C3	-0.39251	-2.569904	1.908942
C4	-1.678839	-2.076238	1.805376
C5	-2.415861	-2.159761	0.593213
C6	-1.785033	-2.83602	-0.486028
H7	-0.039625	-3.814782	-1.230526
H8	0.147728	-2.461194	2.843642
H9	-2.141871	-1.581996	2.653372

H10	-2.320812	-2.940281	-1.422606
N11	0.156267	2.418079	1.317867
N12	-1.480123	3.063137	-0.164568
C13	0.638345	1.544399	2.397183
C14	0.15485	1.952513	3.784778
C15	-1.097943	2.389292	0.906851
C16	-1.275768	1.508411	4.07999
C17	1.165572	3.267627	0.670111
C18	-2.136023	1.607176	1.650922
C19	-2.326019	2.047548	3.110753
C20	0.523993	4.398965	-0.110524
C21	-0.575297	3.843403	-0.996833
H22	1.726159	1.587157	2.345004
H23	0.348674	0.512935	2.175337
H24	0.255171	3.038582	3.891687
H25	0.826963	1.495416	4.517066
H26	-1.54397	1.812366	5.096387
H27	-1.311337	0.412059	4.058769
H28	1.774896	2.636804	0.016409
H29	1.807745	3.661906	1.459335
H30	-3.075591	1.707641	1.109338
H31	-1.866826	0.548085	1.601103
H32	-2.363389	3.141512	3.160202
H33	-3.308519	1.686764	3.426436
H34	1.284273	4.892926	-0.716247
H35	0.101852	5.137241	0.576856
H36	-1.153786	4.636933	-1.468517
H37	-0.158244	3.201558	-1.779281
O38	-3.586822	-1.620126	0.492609
H39	-2.44978	2.992195	-0.433062
C40	6.319855	0.270595	-1.723415
C41	4.970886	0.186625	-2.049251
C42	3.986547	0.496572	-1.105202
C43	4.390987	0.893	0.174494
C44	5.739677	0.976185	0.500643
C45	6.709884	0.665496	-0.447294
H46	7.068312	0.019444	-2.467339
H47	4.680163	-0.139382	-3.042409
H48	3.643957	1.15253	0.917684
H49	6.033031	1.290532	1.496623
H50	7.762328	0.730213	-0.193376
C51	2.543531	0.411886	-1.444242

C52	1.617077	-0.026839	-0.498188
C53	2.06547	0.797178	-2.701752
C54	0.260776	-0.073302	-0.788602
H55	1.955178	-0.341944	0.482724
C56	0.709826	0.739787	-2.994306
H57	2.755318	1.167927	-3.452656
C58	-0.210206	0.299614	-2.041276
H59	-0.438264	-0.408027	-0.033395
H60	0.360592	1.054121	-3.973539
C61	-1.670283	0.207853	-2.418444
H62	-1.818966	-0.690984	-3.026976
H63	-1.941648	1.068766	-3.036625
N64	-2.510932	0.169976	-1.222649
S65	-3.953102	-0.188601	-1.218404
O66	-4.960143	0.335939	-0.344416
F67	-4.486846	0.514636	-2.690789
F68	-4.434154	-1.598777	-1.893764
C69	1.608207	-3.69878	0.914756
F70	1.674284	-5.00857	1.287873
F71	2.35203	-3.031975	1.82474
F72	2.283355	-3.629106	-0.253748

Product complex

Thermal correction to Energy: 0.615918

Thermal correction to Enthalpy: 0.616862

Thermal correction to Gibbs Free Energy: 0.505562

Sum of electronic and zero-point Energies: -2335.836011

Sum of electronic and thermal Energies: -2335.799071

Sum of electronic and thermal Enthalpies: -2335.798127

Sum of electronic and thermal Free Energies: -2335.909427

Center Number	X	Y	Z
C1	0.90436	-3.614279	1.181541
C2	0.136949	-2.91007	0.255788
C3	0.736185	-2.194088	-0.771621
C4	2.120986	-2.172996	-0.873626

C5	2.86599	-2.87518	0.054438
C6	2.285782	-3.60648	1.079576
H7	0.428899	-4.172145	1.97968
H8	0.138524	-1.644958	-1.487609
H9	2.615667	-1.614244	-1.657929
H10	2.897831	-4.15473	1.784907
N11	3.04003	3.910624	1.650334
N12	3.086839	2.763158	3.65746
C13	3.61593	4.202681	0.332837
C14	4.812063	5.150019	0.378676
C15	3.654855	3.128871	2.530641
C16	6.115791	4.46457	0.782367
C17	1.711311	4.470355	1.922187
C18	5.05153	2.645567	2.267816
C19	6.085995	3.775888	2.145892
C20	1.426666	4.51232	3.411714
C21	1.733972	3.156363	4.019881
H22	2.809771	4.643719	-0.253093
H23	3.887643	3.263917	-0.158463
H24	4.581917	5.977258	1.059793
H25	4.934235	5.581968	-0.619306
H26	6.925304	5.201083	0.775789
H27	6.365732	3.715754	0.020153
H28	0.96495	3.862422	1.401054
H29	1.686681	5.476189	1.499176
H30	5.320811	1.984464	3.091391
H31	5.045665	2.036001	1.358206
H32	5.913853	4.511329	2.939625
H33	7.069379	3.337293	2.335102
H34	0.380002	4.774354	3.57197
H35	2.04631	5.277692	3.888089
H36	1.674513	3.181309	5.108552
H37	1.027286	2.40259	3.657216
O38	4.259583	-2.879841	-0.107873
H39	3.617548	2.127069	4.321943
C40	-3.570272	1.843312	-0.478408
C41	-2.44053	1.250127	0.073008
C42	-1.196703	1.888549	0.019294
C43	-1.118328	3.138793	-0.604299
C44	-2.247764	3.731988	-1.156612
C45	-3.479104	3.086928	-1.095967
H46	-4.523481	1.327659	-0.431205

H47	-2.524758	0.273141	0.53555
H48	-0.169102	3.662015	-0.646112
H49	-2.165492	4.704483	-1.630147
H50	-4.360437	3.549428	-1.526789
C51	0.010185	1.25439	0.609821
C52	1.265893	1.402664	0.014811
C53	-0.075466	0.492222	1.778267
C54	2.392969	0.810628	0.565974
H55	1.366518	1.973979	-0.901878
C56	1.052292	-0.104297	2.325228
H57	-1.030844	0.369802	2.276388
C58	2.300352	0.048816	1.726567
H59	3.352475	0.932023	0.076178
H60	0.958954	-0.694667	3.231626
C61	3.508846	-0.583037	2.374222
H62	3.227995	-1.52843	2.844431
H63	3.87773	0.068745	3.171409
N64	4.625816	-0.752077	1.438077
S65	5.194159	-1.986348	0.867067
O66	6.458379	-1.950226	0.199335
F67	4.290608	1.199963	5.294614
F68	5.332583	-3.125923	2.036269
C69	-1.357264	-2.94584	0.394122
F70	-1.839575	-4.204246	0.301033
F71	-1.987048	-2.222307	-0.542773
F72	-1.767978	-2.477412	1.59056

Reaction II with DBU-H⁺

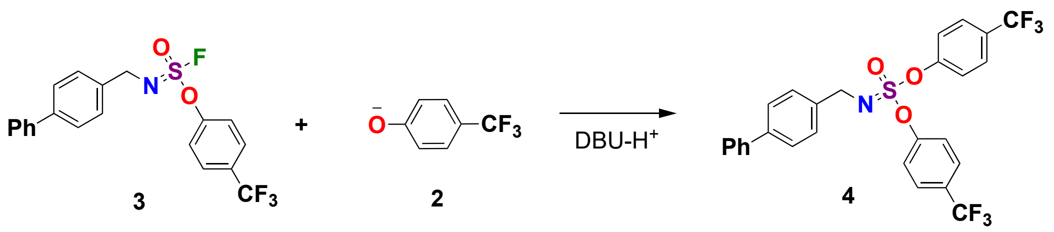

Reactant complex

Thermal correction to Energy: 0.721719

Thermal correction to Enthalpy: 0.722663

Thermal correction to Gibbs Free Energy: 0.588526

Sum of electronic and zero-point Energies: -2879.766994

Sum of electronic and thermal Energies: -2879.720901

Sum of electronic and thermal Enthalpies: -2879.719957

Sum of electronic and thermal Free Energies: -2879.854094

Center Number	X	Y	Z
C1	-9.010119	0.958842	-2.111005
C2	-7.655003	0.7599	-1.872221
C3	-7.207256	-0.382406	-1.200209
C4	-8.15428	-1.319445	-0.773631
C5	-9.509413	-1.120189	-1.01167
C6	-9.942947	0.019921	-1.681477
H7	-9.337998	1.854513	-2.627739
H8	-6.939483	1.508485	-2.195956
H9	-7.82769	-2.219984	-0.26406
H10	-10.227693	-1.861282	-0.677649
H11	-11.000111	0.175785	-1.867078
C12	-5.759308	-0.592177	-0.94153
C13	-5.317489	-1.118774	0.273744
C14	-4.800445	-0.253878	-1.902884
C15	-3.961536	-1.290737	0.524276
H16	-6.035739	-1.366167	1.048088
C17	-3.447446	-0.431912	-1.653535
H18	-5.116417	0.14242	-2.862166
C19	-3.012782	-0.948622	-0.434143
H20	-3.639022	-1.677199	1.485937
H21	-2.721033	-0.161747	-2.412782
C22	-1.549118	-1.179485	-0.162587
H23	-1.230583	-2.090785	-0.680111
H24	-1.389795	-1.328143	0.908469
N25	-0.767424	-0.018777	-0.632137
S26	0.714541	-0.080418	-0.644305
O27	1.557128	-0.778125	0.288029
F28	1.25574	-0.672894	-2.100494
C29	4.552372	2.235596	0.214458
C30	5.166145	2.441521	-1.016402
C31	4.455473	2.29806	-2.205736
C32	3.117285	1.936823	-2.163135
C33	2.534814	1.73006	-0.924711

C34	3.211323	1.878081	0.271711
H35	5.11182	2.350033	1.134777
H36	4.937543	2.46082	-3.161878
H37	2.537749	1.817244	-3.06976
H38	2.708881	1.717041	1.227768
O39	1.158867	1.41934	-0.895404
C40	6.633966	2.750555	-1.070418
F41	7.374727	1.626123	-1.209009
F42	7.077558	3.360568	0.040997
F43	6.957708	3.544004	-2.106997
C44	-1.903147	0.903093	2.787935
C45	-2.311019	2.047448	2.09446
C46	-1.335709	2.968485	1.682974
C47	-0.00448	2.766047	1.970216
C48	0.456136	1.617851	2.702495
C49	-0.570024	0.688418	3.07889
H50	-2.641193	0.173598	3.105209
H51	-1.633583	3.858161	1.134553
H52	0.735765	3.491941	1.646454
H53	-0.272633	-0.205855	3.619365
O54	1.681259	1.447651	2.99291
C55	-3.73498	2.31565	1.796717
F56	-4.13324	3.56051	2.188873
F57	-4.580815	1.454425	2.396558
F58	-4.04047	2.272142	0.468658
H59	5.868115	-1.651686	-1.1156
H60	5.914435	-3.109273	1.187188
C61	4.878129	-1.343906	-0.766803
H62	5.661186	-0.687443	1.125415
C63	4.914265	-2.685389	1.319978
C64	4.829196	-1.282906	0.747872
H65	4.639965	-0.380642	-1.215245
N66	3.882792	-2.303313	-1.229499
N67	3.940331	-3.584508	0.6809
H68	4.687632	-2.673462	2.386547
C69	3.454958	-3.333125	-0.520351
H70	4.26091	-4.845439	2.269942
H71	3.716379	-5.66921	0.820172
H72	3.416921	-2.124857	-2.105899
C73	3.556462	-4.783565	1.441389
H74	2.764202	-5.212872	-1.217848
H75	3.897096	-0.810243	1.062584

C76	2.379286	-4.19272	-1.117888
H77	2.188529	-3.826322	-2.127653
C78	2.124425	-4.741789	1.967721
H79	2.055327	-5.453799	2.795376
C80	1.068657	-4.197323	-0.313253
H81	1.249309	-6.136804	0.591692
H82	1.929648	-3.746741	2.383461
C83	1.077569	-5.102939	0.916155
H84	0.823851	-3.173418	-0.019405
H85	0.277624	-4.530656	-0.989941
H86	0.084885	-5.078346	1.375841

TS

Imaginary Frequency: -194.581 cm^{-1}

Thermal correction to Energy: 0.720124

Thermal correction to Enthalpy: 0.721068

Thermal correction to Gibbs Free Energy: 0.58678

Sum of electronic and zero-point Energies: -2879.756162

Sum of electronic and thermal Energies: -2879.710786

Sum of electronic and thermal Enthalpies: -2879.709842

Sum of electronic and thermal Free Energies: -2879.84413

Center Number	X	Y	Z
C1	9.024771	-0.352409	-1.619724
C2	7.67174	-0.124287	-1.397666
C3	7.163002	1.177695	-1.342011
C4	8.0506	2.245248	-1.517417
C5	9.404335	2.017764	-1.737904
C6	9.897798	0.717717	-1.789652
H7	9.398469	-1.370335	-1.651961
H8	7.006892	-0.967167	-1.249107
H9	7.678708	3.264243	-1.497935
H10	10.074154	2.85973	-1.876748

H11	10.953836	0.54014	-1.961722
C12	5.716179	1.419343	-1.103423
C13	5.284249	2.503048	-0.329277
C14	4.745191	0.573537	-1.642374
C15	3.933883	2.727689	-0.110053
H16	6.012251	3.167583	0.123914
C17	3.390572	0.799149	-1.41965
H18	5.044465	-0.269705	-2.256072
C19	2.968673	1.876804	-0.650805
H20	3.624577	3.567331	0.505887
H21	2.656163	0.123784	-1.842007
C22	1.513395	2.157183	-0.352272
H23	1.257393	3.151197	-0.734474
H24	1.378743	2.181945	0.733268
N25	0.634002	1.168972	-0.975623
S26	-0.610544	0.712129	-0.284805
O27	-1.499631	1.335113	0.667067
F28	-1.752171	1.047668	-1.697494
C29	-3.870667	-2.144971	0.962489
C30	-4.368852	-2.902719	-0.089705
C31	-3.690291	-2.965193	-1.305338
C32	-2.522228	-2.241262	-1.477151
C33	-2.044971	-1.480251	-0.419292
C34	-2.689745	-1.431365	0.806342
H35	-4.39216	-2.106173	1.910272
H36	-4.078502	-3.560806	-2.123273
H37	-1.984253	-2.256586	-2.416507
H38	-2.27905	-0.852424	1.621974
O39	-0.825291	-0.848234	-0.629578
C40	-5.64424	-3.675354	0.063953
F41	-5.428931	-5.008267	0.129401
F42	-6.479516	-3.483793	-0.977952
F43	-6.326193	-3.344141	1.172017
C44	3.932053	-0.601657	2.13668
C45	4.184173	-1.701629	1.313636
C46	3.141504	-2.25171	0.568294
C47	1.872352	-1.703428	0.622239
C48	1.594658	-0.57353	1.428429
C49	2.666541	-0.050858	2.19191
H50	4.7325	-0.166683	2.725306
H51	3.323272	-3.113454	-0.065525
H52	1.07512	-2.132757	0.02926

H53	2.478018	0.819817	2.811515
O54	0.41512	-0.013453	1.490035
C55	5.531496	-2.329276	1.286085
F56	5.658791	-3.355853	2.173925
F57	6.52294	-1.467608	1.591141
F58	5.839732	-2.862432	0.083019
H59	-5.753373	0.688383	-2.274927
H60	-7.722544	1.824188	-0.823386
C61	-5.314452	0.422291	-1.308527
H62	-7.125533	-0.523937	-0.625904
C63	-7.09367	1.51908	0.018549
C64	-6.395893	0.202827	-0.267902
H65	-4.697482	-0.463631	-1.44504
N66	-4.446039	1.500143	-0.855893
N67	-6.122414	2.590729	0.287573
H68	-7.727458	1.430285	0.902112
C69	-4.864016	2.50559	-0.109964
H70	-7.710895	3.670127	1.015846
H71	-6.367403	4.667458	0.489501
H72	-3.457597	1.453605	-1.093889
C73	-6.624879	3.754191	1.032935
H74	-4.181514	4.513809	-0.061
H75	-5.947147	-0.187925	0.649271
C76	-3.857385	3.535248	0.307356
H77	-2.918967	3.296238	-0.190461
C78	-6.120651	3.819407	2.472059
H79	-6.801216	4.465946	3.033995
C80	-3.637668	3.584817	1.828754
H81	-4.695541	5.406251	2.241554
H82	-6.190385	2.81949	2.915497
C83	-4.700598	4.368313	2.597048
H84	-3.561879	2.562993	2.214063
H85	-2.665248	4.053783	1.998737
H86	-4.420533	4.397476	3.654474

Product complex

Thermal correction to Energy: 0.721758

Thermal correction to Enthalpy: 0.722702

Thermal correction to Gibbs Free Energy: 0.59111

Sum of electronic and zero-point Energies: -2879.796952

Sum of electronic and thermal Energies: -2879.751449

Sum of electronic and thermal Enthalpies: -2879.750505

Sum of electronic and thermal Free Energies: -2879.882097

Center Number	X	Y	Z
C1	8.494543	0.965286	-1.143544
C2	7.143315	0.921406	-0.820718
C3	6.52141	2.002258	-0.187108
C4	7.292862	3.13122	0.110366
C5	8.644684	3.175367	-0.211394
C6	9.252011	2.092066	-0.838908
H7	8.957991	0.112527	-1.628126
H8	6.570093	0.030865	-1.04998
H9	6.830097	3.991419	0.582486
H10	9.223005	4.062662	0.02326
H11	10.306719	2.126226	-1.089347
C12	5.076879	1.954966	0.159139
C13	4.599432	2.544337	1.335497
C14	4.153742	1.323141	-0.676905
C15	3.24926	2.512652	1.650899
H16	5.292658	3.017181	2.022998
C17	2.800499	1.289664	-0.358124
H18	4.488851	0.860938	-1.599569
C19	2.331722	1.888901	0.805608
H20	2.905335	2.966973	2.575574
H21	2.104732	0.787372	-1.019909
C22	0.867431	1.897658	1.185904
H23	0.470428	2.909148	1.056872
H24	0.775547	1.660938	2.25104
N25	0.081383	1.002313	0.331401
S26	-0.812045	-0.055929	0.88324
O27	-1.998139	0.14667	1.68102
F28	-6.014313	0.478366	-3.299893
C29	-3.875065	-3.300437	0.180652
C30	-4.791295	-2.877737	-0.781695
C31	-4.505263	-1.807941	-1.61874
C32	-3.296478	-1.13815	-1.48187
C33	-2.405026	-1.563498	-0.516691

C34	-2.662965	-2.644134	0.313566
H35	-4.10285	-4.140154	0.827056
H36	-5.218634	-1.450718	-2.353176
H37	-3.057765	-0.288489	-2.109007
H38	-1.939417	-2.961589	1.053925
O39	-1.182304	-0.883614	-0.446429
C40	-6.104202	-3.596747	-0.882867
F41	-6.767769	-3.600399	0.293743
F42	-5.948258	-4.894261	-1.225846
F43	-6.9308	-3.056237	-1.789902
C44	3.601989	-1.314062	2.187918
C45	3.972163	-2.006936	1.040424
C46	3.014765	-2.435084	0.125231
C47	1.673913	-2.163692	0.351741
C48	1.321867	-1.459303	1.492881
C49	2.263293	-1.032937	2.415041
H50	4.348246	-0.981325	2.898466
H51	3.307411	-2.979658	-0.764276
H52	0.917733	-2.492203	-0.349885
H53	1.948987	-0.479437	3.291393
O54	-0.01707	-1.192188	1.775849
C55	5.413261	-2.351349	0.808083
F56	5.715979	-3.588403	1.266698
F57	6.255604	-1.505739	1.423582
F58	5.737085	-2.348205	-0.498259
H59	-7.297674	0.906858	0.422141
H60	-5.89487	2.155301	2.345655
C61	-6.358813	0.411917	0.151402
H62	-5.972144	-0.252599	2.170558
C63	-5.058411	1.657864	1.844477
C64	-5.457618	0.273985	1.365592
H65	-6.601804	-0.555976	-0.28555
N66	-5.6661	1.197124	-0.858655
N67	-4.611537	2.503308	0.728392
H68	-4.227259	1.592235	2.549475
C69	-4.859793	2.182764	-0.536806
H70	-4.070211	3.877921	2.154407
H71	-4.3028	4.573035	0.561663
H72	-5.784917	0.928478	-1.879267
C73	-3.873839	3.719453	1.093763
H74	-4.481072	3.969218	-1.629853
H75	-4.565671	-0.298252	1.095109

C76	-4.172136	2.918816	-1.649745
H77	-4.534932	2.495699	-2.586538
C78	-2.371439	3.627142	0.848366
H79	-1.886518	4.410159	1.439621
C80	-2.638644	2.815911	-1.582715
H81	-2.220548	4.817247	-0.932245
H82	-2.019019	2.667188	1.235553
C83	-1.974408	3.795647	-0.616381
H84	-2.355609	1.789118	-1.329201
H85	-2.253861	3.004028	-2.588485
H86	-0.888291	3.692184	-0.704591

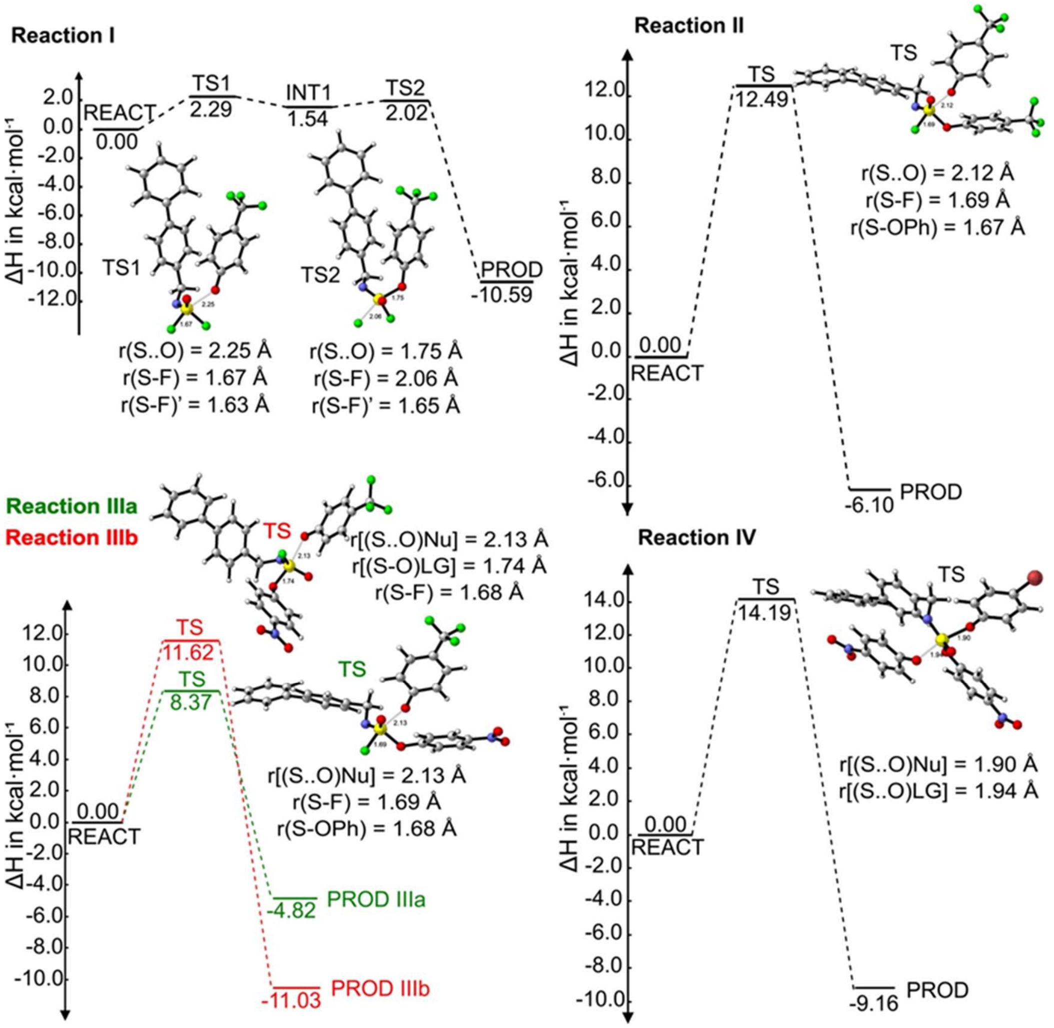

Figure S47. Reaction profile for reactions **I**, **II**, **IIIA**, **IIIB**, and **IV** calculated at the ωB97XD/6-311+G(d,p) level of theory. (Identical to Figure 2 in the paper - enlarged image for interested readers).

11. References:

(1) Dong, J.; Ke, S.; Li, S. Preparation methods and systems of SOF_2 and SOF_4 gases. Chinese Patent CN 108128758 B, Jun 08, 2018.

(2) Dolomanov, O.V.; Bourhis, L.J.; Gildea, R.J.; Howard, J.A.K.; Puschmann, H., OLEX2: A complete structure solution, refinement and analysis program (2009). J. Appl. Cryst., **42**, 339-341.

(3) Frisch, M. J.; Trucks, G. W.; Schlegel, H. B.; Scuseria, G. E.; Robb, M. A.; Cheeseman, J. R.; Scalmani, G.; Barone, V.; Petersson, G. A.; Nakatsuji, H.; Li, X.; Caricato, M.; Marenich, A. V.; Bloino, J.; Janesko, B. G.; Gomperts, R.; Mennucci, B.; Hratchian, H. P.; Ortiz, J. V.; Izmaylov, A. F.; Sonnenberg, J. L.; Williams-Young, D.; Ding, F.; Lipparini, F.; Egidi, F.; Goings, J.; Peng, B.; Petrone, A.; Henderson, T.; Ranasinghe, D.; Zakrzewski, V. G.; Gao, J.; Rega, N.; Zheng, G.; Liang, W.; Hada, M.; Ehara, M.; Toyota, K.; Fukuda, R.; Hasegawa, J.; Ishida, M.; Nakajima, T.; Honda, Y.; Kitao, O.; Nakai, H.; Vreven, T.; Throssell, K.; Montgomery, J. A., Jr.; Peralta, J. E.; Ogliaro, F.; Bearpark, M. J.; Heyd, J. J.; Brothers, E. N.; Kudin, K. N.; Staroverov, V. N.; Keith, T. A.; Kobayashi, R.; Normand, J.; Raghavachari, K.; Rendell, A. P.; Burant, J. C.; Iyengar, S. S.; Tomasi, J.; Cossi, M.; Millam, J. M.; Klene, M.; Adamo, C.; Cammi, R.; Ochterski, J. W.; Martin, R. L.; Morokuma, K.; Farkas, O.; Foresman, J. B.; Fox, D. J. Gaussian 16, Revision C.01; Gaussian, Inc., Wallingford CT, 2016. https://gaussian.com/citation/
